# Resolving sensitivity, specificity and signal contamination in Xenium spatial transcriptomics

**DOI:** 10.1038/s41592-026-03089-8

**Published:** 2026-04-30

**Authors:** Mariia Bilous, Daria Buszta, Jonathan Bac, Senbai Kang, Yixing Dong, Stephanie Tissot, Sylvie Andre, Marina Alexandre Gaveta, Christel Voize, Solange Peters, Krisztian Homicsko, Raphael Gottardo

**Affiliations:** 1 Biomedical Data Science Center, Lausanne University Hospital, University of Lausanne, Lausanne, Switzerland; 2 Department of Oncology, Lausanne University Hospital, Swiss Cancer Center Leman, Lausanne, Switzerland; 3 Ludwig Institute for Cancer Research, Lausanne, Switzerland; 4 Agora Translational Research Center, Lausanne, Switzerland; 5 Swiss Institute of Bioinformatics, Lausanne, Switzerland; 6 Faculty of Biology and Medicine, University of Lausanne, Lausanne, Switzerland; 7 School of Life Sciences, Ecole Polytechnique Fédérale de Lausanne, Lausanne, Switzerland

**Keywords:** Gene expression, Software, Cancer imaging

## Abstract

Spatial transcriptomics enables high-resolution gene expression mapping in intact tissues. Xenium is widely adopted for its reliability, accessibility and data quality, yet the properties and limitations of Xenium-derived data remain poorly characterized. Here we present one of the most comprehensive Xenium datasets so far, encompassing over 40 breast and lung tumor sections profiled using diverse gene panels. Leveraging this resource, we systematically dissect technical noise—including transcript spillover—along with assay specificity, panel performance and segmentation strategies. We demonstrate that single-nucleus RNA sequencing enables precise quantification of transcript contamination. Building on these insights, we introduce SPLIT (Spatial Purification of Layered Intracellular Transcripts), a method that improves signal purity by resolving mixed transcriptomic signals. SPLIT enhances background correction and cell-type resolution and enables the revelation of T-cell exhaustion signatures associated with malignant cell colocalization—signals that would otherwise remain obscured. Together, our findings provide a critical benchmark for Xenium performance and introduce a scalable strategy for signal refinement.

## Abstract (web summary)

This study investigates technical noise in Xenium spatial transcriptomics data, including transcript spillover, and introduces SPLIT to resolve mixed signals and enhance cell-type specificity.

## Main

Recent technological advances have led to the development of spatially resolved transcriptomics^1^, which enables the spatial quantification of gene expression within tissues. These technologies provide researchers and clinicians with an unprecedented ability to characterize patient samples, including archived formalin-fixed, paraffin-embedded (FFPE) specimens^2^, with high spatial resolution.

Imaging-based spatial transcriptomics technologies such as Xenium^3^, MERSCOPE^4^ and CosMx^5^ offer high-resolution transcript quantification at the cellular—and even subcellular—level. These platforms, as well as their earlier iterations, have already generated valuable biological insights and continue to hold considerable promise for advancing our understanding of complex tissues^6–8^. However, our understanding of the data characteristics and sources of variability is still maturing as more datasets become available. Key experimental design questions remain, particularly around panel selection (for example, targeted versus 5K Xenium panels). Larger panels increase multiplexing but often reduce sensitivity, increase cost and lengthen processing time, and comparative data remain limited.

Janesick et al.^3^ were the first to present data generated on the Xenium platform and to perform an in-depth comparison with Visium and Chromium, single-nucleus RNA sequencing (snRNA-seq), using adjacent tissue sections from two samples. Their study demonstrated that the Xenium platform provides higher resolution and sensitivity, while also highlighting the potential for integrating multiple platforms. Cook et al.^9^ performed a direct comparison of Xenium and CosMx from a single FFPE block of one patient. Their findings indicated better segmentation in CosMx but higher sensitivity in Xenium. Ren et al.^10^ conducted an in-depth comparison of Xenium 5K, CosMx 6K and Visium HD but again analyzed only three samples. More recently, Wang et al.^11^ compared Xenium (multiple targeted panels, but no 5K) with MERSCOPE and CosMx using 13 samples but limited to tissue microarrays. While they reported similar findings, their analysis was limited, without touching on critical factors such as segmentation and transcript contamination as we discuss below. One of the most comprehensive analyses so far is that of Marco Salas et al.^12^, who performed a systematic quality assessment of the Xenium platform by analyzing 25 primarily public samples across multiple tissues and benchmarking its performance against other spatial transcriptomics technologies. Their results consistently confirmed the high sensitivity of Xenium and provided valuable recommendations for analytical strategies. Overall, existing studies remain limited in scale—typically including only a few patients (or relying on tissue microarrays)—and none has directly compared targeted Xenium panels with the newer 5K panel in terms of transcript sensitivity and specificity, factors that are essential for robust downstream analyses.

Multiple studies have demonstrated that individual transcripts may be incorrectly assigned to cells, either due to inaccurate segmentation or transcript spillover into adjacent cells^13,14^—leading to background contamination^12^ that can affect downstream analyses. Transcript misassignments can result in the formation of artificial transcriptomic doublets (or multiplets), where gene expression signatures from different cell types become mixed and ambiguous. As we demonstrate later in this study, these mixed profiles do not correspond to actual physical doublets as defined in the single-cell sequencing field, but instead arise from signal-level mixing within spatial data. While some of this signal mixing can be attributed to cells overlapping along the *z* axis^12,15^, causing spillover during *x*–*y* plane segmentation—this alone does not fully account for the extent of the contamination, as we explore in this study. Indeed, Marco Salas et al.^12^ reported contamination associated with cellular expansion in the standard Xenium segmentation, suggesting that transcript spillover within the *x*–*y* plane contributes substantially to background signal. However, aside from providing an automated method to adjust the expansion parameter, they did not propose a mechanism to correct this contamination. Mitchel et al.^16^ demonstrated that transcript misassignment can generate artificial cell–cell correlations (or communication signals), resulting in incorrect biological interpretations. To address this challenge, they proposed a correction method that leverages weighted non-negative matrix factorization and conditional random fields to reduce signal contamination. Ergen and Yosef^14^ framed this problem as transcript diffusion (or spillover) and introduced a probabilistic approach to correct for it. Wu, Beechem and Danaher^17^ proposed a comparable method for CosMx data, reassigning transcripts to their most likely cell of origin. These findings suggest that transcript spillover is a widespread challenge across spatial transcriptomic platforms.

Probabilistic segmentation methods that leverage transcript-level data have emerged as powerful tools for improving cell-level gene expression quantification, either by reducing signal spillover or enhancing morphological segmentation. One of the first such methods, Baysor, introduced by Petukhov et al.^18^, modeled spatial transcript distributions for cell segmentation. Building on this, Jones et al.^19^ developed ProSeg, a three-dimensional segmentation method that leverages the spatial coordinates of each transcript. In addition, several other algorithms have been developed that incorporate transcript-level information, including Segger^20^, BIDCell^21^ and FastReseg^17^, reflecting a broader shift toward data-driven segmentation strategies for resolving transcript misassignment. As we discuss later, our data suggest that transcript misassignment is probably driven, at least in part, by transcript spillover. Because transcript spillover and segmentation-related artifacts are often addressed using similar computational strategies—and spillover appears to be the dominant source of signal distortion in our data—we focus on ‘transcript spillover’ throughout this study.

Despite recent progress, existing datasets remain small and limited in scope, lacking the generalizability needed for broader application. Computational methods that leverage transcript-level data have shown clear improvements in signal purity; however, many still depend on heuristic corrections or complex, non-interpretable models, and have been validated on only a limited number of samples. These limitations highlight the need for larger, more representative studies and the development of simpler, more interpretable approaches for correcting spatial transcriptomic data.

Here, we present one of the most comprehensive Xenium datasets generated so far, spanning 41 tissue sections from breast and lung tumors across 27 donors. This resource was designed to investigate key challenges in spatial transcriptomics, including transcript spillover, assay specificity, panel performance (targeted versus 5K), segmentation and cell-type annotation. We also demonstrate how a snRNA-seq reference can be used to streamline cell-type annotation, evaluate data quality and mitigate transcript spillover. Finally, we introduce SPLIT (Spatial Purification of Layered Intracellular Transcripts), a simple yet effective computational method that integrates snRNA-seq with robust cell type decomposition (RCTD)^22^ deconvolution to enhance signal purity. SPLIT resolves mixed signals caused by transcript spillover, improves cell-type separation and is compatible with any segmentation strategy.

## Results

### Data and experimental overview

We generated spatial transcriptomics data from 19 sections derived from FFPE blocks of breast cancer tissue and 22 sections from FFPE blocks of non-small-cell lung cancer (NSCLC), corresponding to 17 and 10 donors, respectively. Xenium profiling was performed on all samples using the targeted Breast panel for breast cancer samples, and the targeted Lung panel, a custom immuno-oncology panel (Custom IO) and the 5K PRIME panel for NSCLC samples. In addition, matched snRNA-seq and immunohistochemistry (IHC) data were generated for a selected subset of samples (see Methods and Supplementary Tables 1 and 2 for details).

Segmentation was performed on all Xenium data, resulting in single-cell resolution for subsequent analysis. For samples profiled with targeted panels, we used the default segmentation approach, which applies a 5-µm radius expansion around each nucleus to approximate cell boundaries. By contrast, for samples profiled with the 5K panel, we leveraged the availability of multimodal staining with a 5-µm radius expansion, which combines nuclear, cytoplasmic and membrane stains to enhance segmentation accuracy.

As described in the next section, snRNA-seq data are used to annotate the Xenium datasets and serve as a reference for evaluating data quality, including cell-type gene expression specificity and resolution. IHC is used to further validate the annotation—particularly to assess the potential misannotation of specific cell types due to transcript spillover (see Methods for details).

**Fig. 1:**
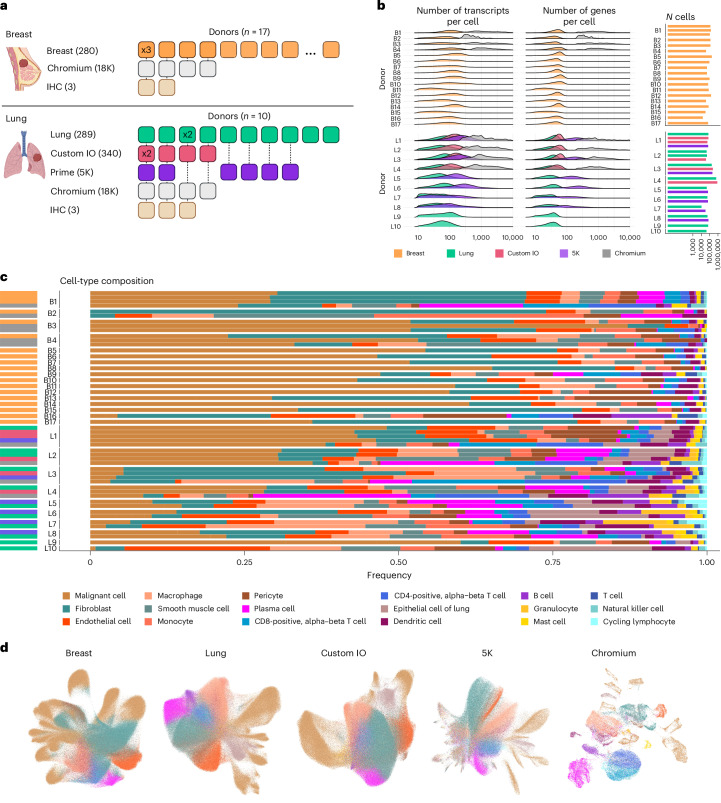
Experimental design and key metrics. **a**, Experiment names correspond to the profiling technology and panel used, with the number in parentheses indicating the panel size. Each sample is represented as a tile, and replicate experiments are marked with ‘x2’ or ‘x3’ within the tile to refer to the number of replicates. Columns group samples originating from the same donor. **b**, Density distributions of the number of transcripts per cell and the number of detected genes per cell for each sample, along with the number of cells per sample, color-coded by panel and assay type. **c**, Cell-type composition of all Xenium and Chromium samples, grouped by donor and colored by cell type. The colored strip alongside each row indicates the corresponding panel or assay. **d**, Integrated UMAPs grouped by panel and assay. Cells are colored by cell type as assigned by RCTD using matched Chromium reference at Level 2.1 annotation.

Figure 1a,b summarizes the experimental design, including the number of cells per sample, the number of transcripts per cell and the number of genes per panel. Despite substantial variability in cell capture across samples—probably due to differences in tissue size and preservation quality—all samples produced high-quality data, with robust transcript and gene detection at the single-cell level.

### snRNA-seq data enable robust annotation of Xenium datasets

While Xenium data inherently provide single-cell resolution, we leveraged partially matched and annotated snRNA-seq datasets (Methods) to transfer cell-type annotations to each Xenium sample. For this, we applied RCTD^22^, a framework originally developed for spatial transcriptomics deconvolution, which we and others^23^ have found to be highly effective for annotating Xenium data (Methods). Importantly, RCTD includes a doublet mode that helps account for mixed signals arising from segmentation errors, overlapping cells or transcript spillover that we discuss later.

Figure 1c shows the distribution of annotated cell populations across individual samples. The data demonstrate strong reproducibility among technical replicates while revealing substantial variability between patients and between technologies—namely, Chromium and Xenium. This variability is expected: Chromium relies on dissociated single nuclei and typically requires larger input numbers, a process known to introduce biases in cell-type representation^24^. While snRNA-seq has been shown to mitigate some of these biases, it does not eliminate them entirely^25^. By contrast, Xenium profiles intact tissue sections, preserving spatial context and reducing dissociation-related artifacts.

**Extended Data Fig. 1:**
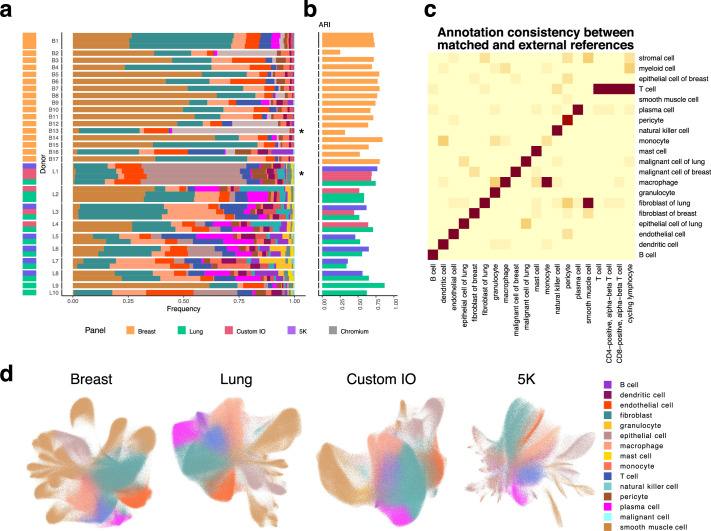
Annotation of Xenium data using external references. **a**, Cell-type composition for all Xenium samples using external references, grouped by donor and colored by cell type. The side color bar indicates the panel. **b**, Adjusted Rand Index (ARI) comparing RCTD annotations from matched vs. external references. Color indicates the panel; * denotes samples where tumor cells were not detected. **c**, Confusion matrix comparing cell-type annotations from external (rows) and matched (columns) reference data. **d**, UMAPs of all cells grouped by panel, colored by RCTD-assigned cell types using external Chromium data.

We explored the robustness of RCTD using nonmatched scRNA-seq references (Extended Data Fig. 1). Overall, we found generally consistent performance, with malignant cell annotation being more challenging in some samples (Extended Data Fig. 1). Given the substantial interpatient heterogeneity in malignant cell transcriptional profiles, our findings highlight the value of a matched reference for cancer research.

Similarly, the annotation remains robust across different Xenium panels, demonstrating that targeted gene panels effectively capture key information for defining major cell types—an aspect we explore further below.

### Xenium data show great consistency across patients and technical replicates

**Extended Data Fig. 2:**
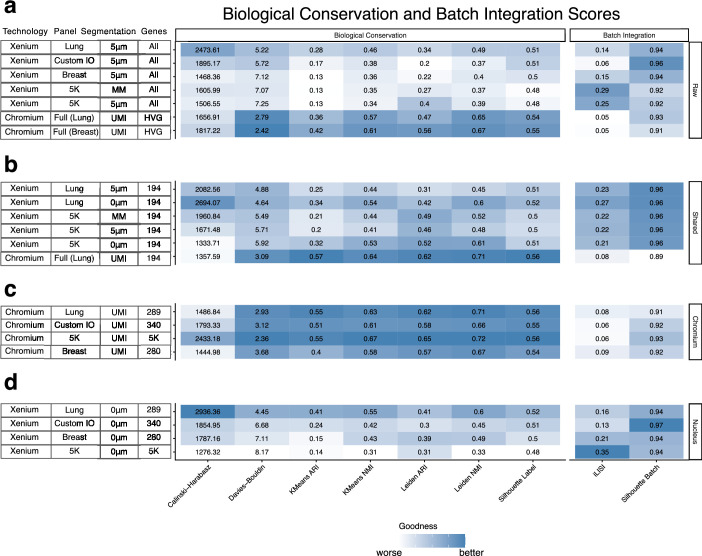
Biological conservation and batch integration scores. **a**, Default data, including all Xenium datasets processed with default segmentations and Chromium datasets processed with the standard pipeline. Corresponding UMAPs are shown in Fig. 1d. **b**, NSCLC datasets restricted to the 194 genes shared between 5 K and Lung panels. For Xenium data, samples from six shared donors are included; for Chromium, all NSCLC samples are used. Corresponding UMAPs are shown in Fig. 2a. **c**, Chromium data restricted to the genes present in the Xenium panel. Corresponding UMAPs are shown in Extended Data Fig. 3h. **d**, Xenium data processed using nuclear segmentation. Color represents the goodness score, with darker shades indicating higher (that is, better) values; color gradient is scaled per column.

**Extended Data Fig. 3:**
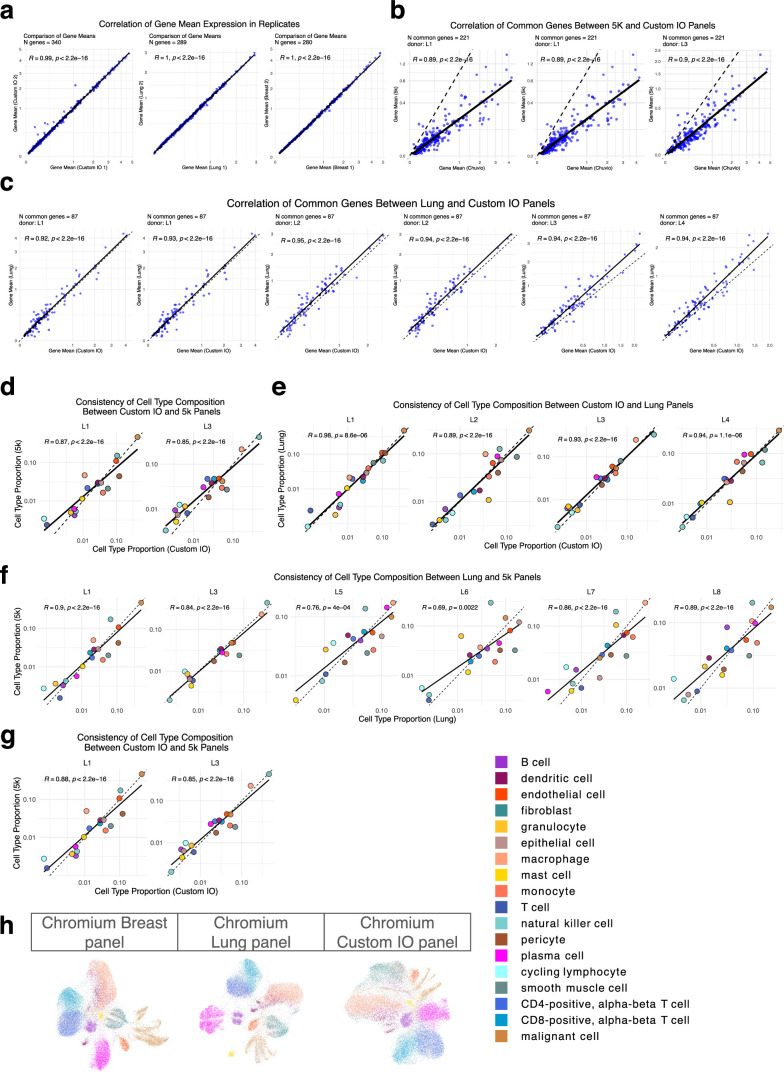
Cross-panel consistency across xenium and chromium. **a**, Spearman correlation of mean gene counts across technical replicates in targeted panels. Each dot represents a gene. **b–c**, Correlation of gene expression between Custom IO and (**b**) Prime 5k and (**c**) Lung panels in matched samples. **d–g**, Scatterplots of RCTD-inferred cell-type compositions across matched samples profiled with different panels: (**d**) Custom IO vs. Prime 5k (with multimodal segmentation); (**e**) Custom IO vs. Lung panel; (**f**) Lung vs. Prime 5k (without multimodal segmentation); (**g**) Custom IO vs. Prime 5k (without multimodal segmentation). **h**, UMAPs of matched Chromium data using only genes in the corresponding targeted panels. R, Spearman correlation coefficient; p-values were computed using a two-sided t test and were not adjusted for multiple comparisons, given the small number (up to n = 6) of independent donor-level tests. The dashed line indicates the identity (y = x). A solid line indicating generalized linear model (GLM) fit is shown for visualization only.

To evaluate the quality and consistency of Xenium single-cell data, we performed Uniform Manifold Approximation and Projection (UMAP)-based joint analyses across all samples, leveraging the transferred cell-type annotations. The data were stratified by panel and assay (Fig. 1d). Supplementary Figs. 1 and 2 present the same dataset faceted by sample and heatmaps of cell–cell distances, revealing that nonmalignant cell populations align consistently across individuals, whereas malignant cells cluster in a patient-specific manner, as anticipated. Batch integration metrics^26^ (iLISI and Silhouette Batch; Extended Data Fig. 2) further confirm strong concordance of cell types across samples, without requiring batch correction or data integration, aside from Seurat log normalization. This indicates that both Xenium and Chromium platforms introduce little technical variation. The discrepancy between the two metrics reflects their differing sensitivities—iLISI captures local neighborhood mixing and is more sensitive, whereas Silhouette Batch evaluates global separation—an expected and previously reported phenomenon^27^. Overall, data from technical replicates derived from adjacent tissue sections show near-perfect concordance (Extended Data Fig. 3a), confirming the high reproducibility of the platform.

### Targeted gene panels outperform the 5K panel in sensitivity

As expected, Fig. 1b shows that the 5K panel detects a higher total number of transcripts and genes. However, this increase does not translate into improved cell-type separation (Fig. 1d) as quantified by multiple cell-type separation metrics, including Calinski–Harabasz, Davies–Bouldin, Leiden adjusted Rand index (ARI), Silhouette score and so on (Extended Data Fig. 2a). To investigate this further, we focused on genes shared across panels (*n* = 194) to assess whether the 5K panel exhibits reduced sensitivity when controlling for gene content.

**Fig. 2:**
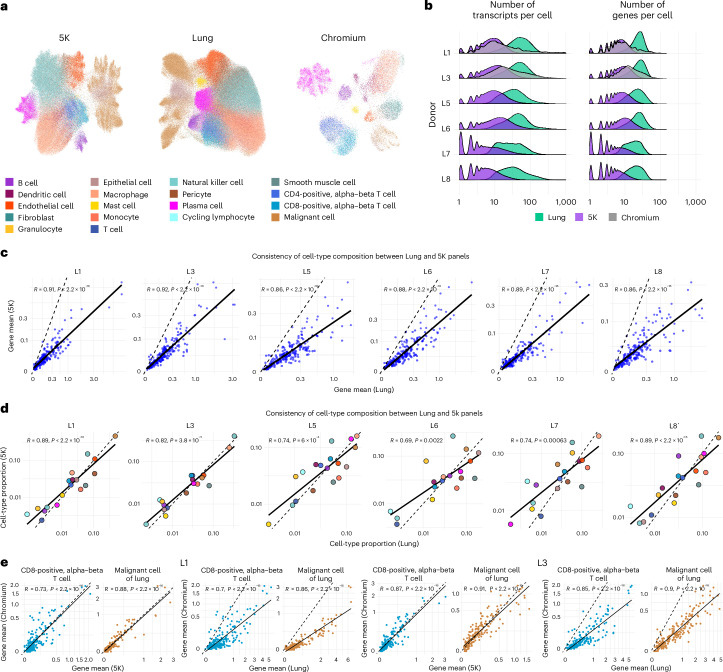
Comparison of Xenium prime (5K) and targeted (Lung) panels. **a**, UMAP visualization of lung cancer samples based on 194 genes common to the Xenium 5K panel, Xenium Lung panel and Chromium data. Xenium UMAPs are restricted to samples profiled with both Lung and 5K panels. Cells are colored according to their annotated cell types at Level 2.1. **b**, Distribution of transcript counts per cell in the Xenium 5K, Xenium Lung and Chromium datasets, restricted to the common genes. **c**, Scatterplot showing average expression of common genes in matched samples from the Xenium 5K and Xenium Lung panels. **d**, Scatterplot showing cell-type composition in matched samples from the Xenium 5K and Xenium Lung panels. **e**, Scatterplots comparing average expression of the 194 common genes between Chromium and Xenium Lung, and between Chromium and Xenium 5K, in CD8^+^ T cells and malignant cells. Analyses are based on matched samples from two donors (L1 and L3) profiled with all three assays. Extended comparisons across all cell types, and a direct comparison between Xenium Lung and Xenium 5K panels, are shown in Extended Data Figs. 3 and 4. *R*, Spearman’s rank correlation coefficient; *P* values were computed using a two-sided *t*-test and were not adjusted for multiple comparisons, given the small number (*n* = 6) of independent donor-level tests. The dashed line indicates the identity (*y* = *x*). A solid line indicating generalized linear model (GLM) fit is shown for visualization only.

Figure 2a and Extended Data Fig. 2b show that the 5K and Lung panels yield a comparable cell-type separation when analysis is restricted to shared genes and matched samples. However, approximately 60% of cells in the 5K dataset fail quality control (QC) owing to low transcript counts, indicating reduced sensitivity compared with the Lung panel. Consistently, for the same gene set, the 5K panel detects considerably fewer transcripts (Fig. 2b). This observed reduction in sensitivity is consistent with known limitations of the 5K panel and probably reflects protocol modifications, such as the reduced probe count to limit optical overcrowding.

We also assessed gene expression consistency between adjacent tissue sections using shared genes across panels. As shown in Fig. 2c and Extended Data Fig. 3b,c, the 5K and targeted panels exhibit a reasonable correlation, although the 5K panel consistently shows lower sensitivity. Notably, the 5K sensitivity is comparable to Chromium (Fig. 2b), underscoring a key trade-off between gene breadth and detection depth. While the 5K panel covers a broader set of genes, these results highlight important limitations in sensitivity.

### Targeted and 5K panels show high concordance in cell-type composition

Despite the reduced sensitivity of the 5K panel, we observe good consistency in cell-type composition between adjacent sections profiled with different panels (Fig. 2d), suggesting that the broader gene coverage of the 5K panel might help compensate for its reduced sensitivity. Similar concordance is observed between the 5K and Custom IO panels (Extended Data Fig. 3d). Notably, the two targeted panels—Custom IO and Lung— exhibit the highest consistency despite their limited gene overlap (*n* = 87) (Extended Data Fig. 3e). This suggests that Xenium is robust to variations in gene panel design—at least within the context of targeted panels that share the same underlying chemistry. To test whether better agreement between targeted panels is explained by segmentation differences, we reanalyzed 5K data using the same 5-μm expansion method but found no notable improvement in correlation (Extended Data Fig. 3f,g).

**Extended Data Fig. 4:**
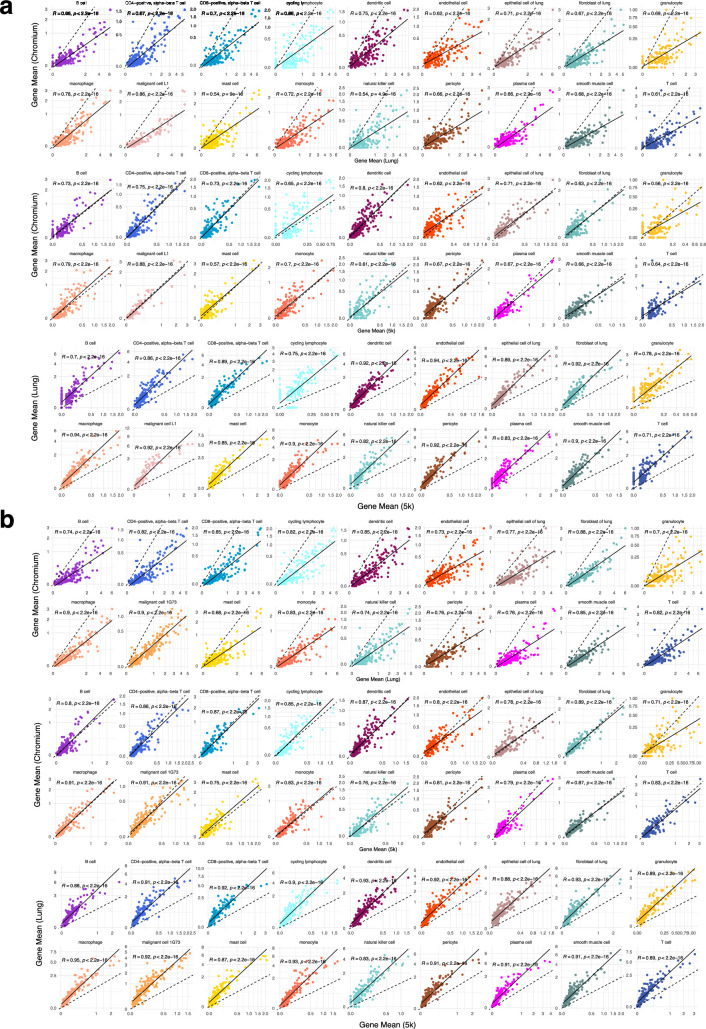
Cell-type profile similarity across panels and assays. Scatterplots compare the average expression of 194 shared genes between Chromium, Xenium Lung, and Xenium 5k per cell type for two donors—L1 (**a**) and L3 (**b**)—profiled with all three technologies. Solid lines indicate trendlines (GLM); dashed lines denote identity (y = x). R denotes Spearman correlation. p-values were computed using a two-sided t test.

To further evaluate the similarity of cell-type profiles between the Xenium platform (5K and targeted panels) and the Chromium reference, we computed correlations across matched donor samples. Both Xenium panels exhibit good agreement with Chromium-derived profiles, with the 5K panel resembling the Chromium data more in terms of sensitivity (Fig. 2e and Extended Data Fig. 4).

### Targeted gene panels provide extensive information for defining major cell types

To assess how gene set size influences cell-type resolution, we reanalyzed the Chromium data using only the genes included in the targeted panels. As shown in Extended Data Fig. 3h and analytically supported in Extended Data Fig. 2c, Chromium data achieve a clear separation of cell types despite the reduced gene sets. Moreover, this separation persisted even when the analysis was further constrained to a subset of 194 genes shared between the targeted Lung and 5K panels (Fig. 2a). These findings suggest that the diminished resolution observed in the Xenium data is not solely due to a limited number of genes analyzed.

Notably, after restricting to shared genes, the Lung and 5K panels still failed to achieve the same level of cell-type separation as observed with Chromium data (Extended Data Fig. 2b,c). This supports the idea that transcript spillover between neighboring cells may blur cell-type boundaries, limiting resolution in spatial transcriptomics.

### Xenium data exhibit substantial transcript spillover

We applied RCTD in doublet mode to annotate Xenium data, modeling each cell as a mixture of a primary and secondary cell type with weights *w*_1_ and *w*_2_, and classifying cells as singlets or doublets (Supplementary Fig. 3a and Methods). Importantly, RCTD ‘doublets’ in Xenium rarely represent true biological doublets but instead reflect signal mixing (Supplementary Note 1); therefore, we refer to cells with evidence secondary as ‘contaminated’ and those without as ‘pure’.

Some studies attribute such admixtures to domain-specific background noise^12^, others ascribe them primarily to vertical (*z* axis) cell overlap^15^, and additional reports show frequent signal mixing within the same *z* plane^16^. As both our data (Supplementary Note 2) and prior works^12^ reported reduced signal admixture when restricting profile to nuclear transcripts, we further investigated the contribution of horizontal transcript spillover to signal contamination.

**Fig. 3:**
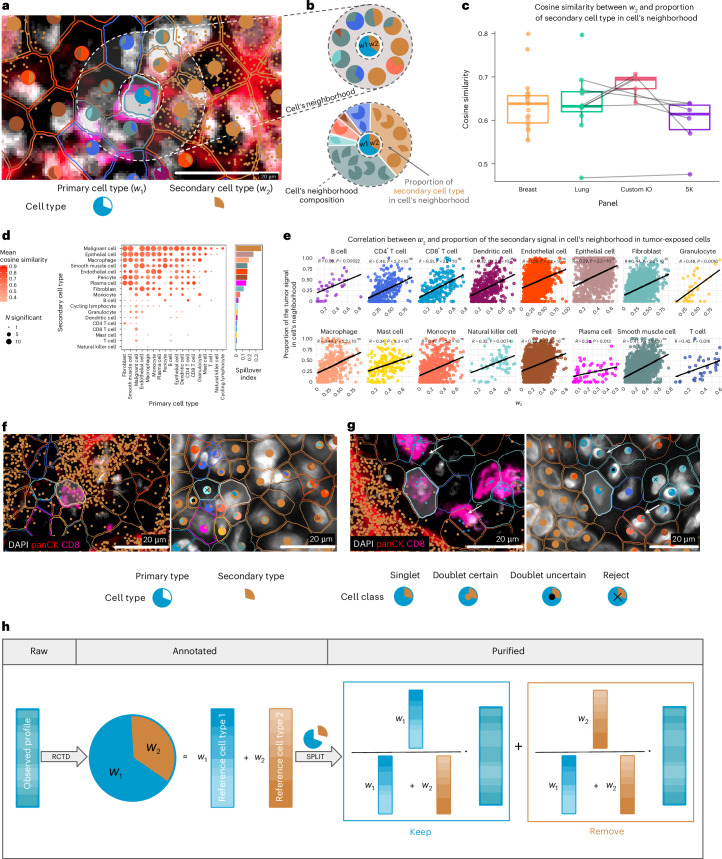
Transcript spillover in Xenium data and the SPLIT purification approach. **a**, Section of a Xenium sample showing cell segmentation overlaid with RCTD-derived cell-type annotations. Border colors indicate the primary cell type assigned to each cell. Pie charts represent RCTD-computed cell-type compositions, where sector size corresponds to the proportion of each cell type and color denotes identity. Small dots represent transcripts specific to tumor cells (brown: *MUC1*, *F3*, *FASN*, *KRT7* and *EPCAM*) and CD8^+^ T cells (blue: *CD8A*). **b**, Schematic illustrating the definition of a cell’s neighborhood (top) and its neighborhood composition (bottom). The local abundance of contaminating cell type is defined as the proportion of the cell’s secondary cell type (brown) within its spatial neighborhood. Colors represent distinct cell types. **c**, Cosine similarity between a cell’s secondary cell-type weight (*w*_2_) and the average proportion of the same cell type in its neighborhood, across all cells and samples. Each dot represents one Xenium sample; lines connect samples from the same donor. Box plots show the median and interquartile range (IQR), with whiskers extending to the nearest value outside 1.5× IQR. Breast: *n* = 19 samples (17 biological and 3 technical replicates); Lung: *n* = 11 samples (10 biological and 2 technical replicates); Custom IO: *n* = 6 samples (5 biological and 2 technical replicates); 5K: *n* = 6 biological replicates. **d**, Cell-type spillover index. Each dot represents the average cosine similarity between a cell’s secondary cell-type weight (*w*_2_) and the proportion of that same cell type in its spatial neighborhood, computed for a given cell-type pair across all Xenium samples profiled with the Lung panel (see Extended Data Fig. 5a for other panels). Dot size indicates the number of samples in which the cosine similarity was statistically significant (*P* value <0.01, one-sided permutation test, *n* = 1,000), and color denotes the average correlation value. The adjacent bar plot summarizes these values per secondary (that is, contaminating) cell type, representing the overall cell-type spillover index. **e**, Spearman correlation between *w*_2_ and the proportion of malignant signal in spatial neighborhood of tumor-exposed cells (cells where the secondary cell type is ‘malignant cell’), faceted by primary cell type. *P* values were computed using a two-sided *t*-test. **f**, IHC and morphology image showing a cell identified as a CD8^+^ T cell by IHC but annotated as a tumor cell by RCTD due to high levels of tumor-specific transcripts. The border color indicates the RCTD-assigned primary cell type. Dots represent cell-type-specific transcripts (tumor in brown, CD8^+^ T cell in blue). **g**, IHC and morphology image of a cell confidently annotated as a CD8^+^ T cell by both IHC and RCTD (singlet), displaying residual tumor-specific transcripts. Arrows indicate cells where the primary and secondary labels appear to be swapped. The border color represents the RCTD-assigned primary cell type; dots show tumor (brown) and CD8^+^ T cell (blue) transcripts. **h**, Schematic of the RCTD cell-type annotation in doublet mode. Each cell is modeled as a mixture of two cell types (primary and secondary) with proportions *w*_1_ and *w*_2_. SPLIT decomposes this mixture into purified primary and secondary components based on reference profiles and assigned weights. By default, the primary profile is retained and used in downstream analysis. Panels **a**, **f** and **g** correspond to samples from the same donor (Lung L1).

For this, we compared contamination levels—estimated by the RCTD-assigned weight of the secondary cell type in each cell (*w*_2_)—with the local relative abundance of that same cell type in the surrounding two-dimensional neighborhood—estimated as normalized sum of the RCTD weights corresponding to this cell type (Fig. 3a,b). These metrics show strong cosine similarity (Fig. 3c), indicating that elevated local concentrations of a given cell type align with increased contamination from that type, consistent with transcript spillover. This relationship is reproducible across publicly available Xenium datasets from multiple tissues and across alternative segmentation strategies (Supplementary Note 3), supporting the generality of transcript spillover.

**Extended Data Fig. 5:**
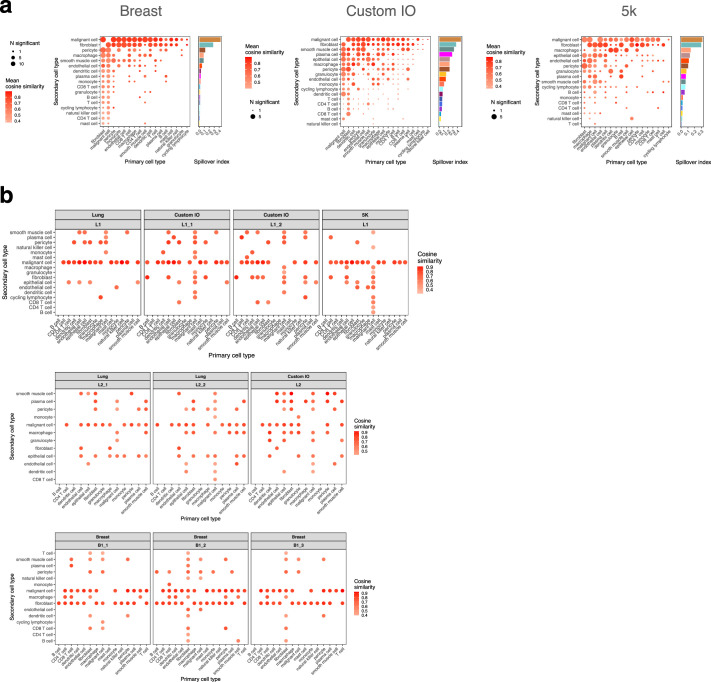
Cell-type spillover index across panels and distribution of contamination level in doublets and singlets. **a**, Each dot represents the average Cosine similarity between a cell’s secondary cell-type weight (w_2_) and the proportion of that same cell type in its spatial neighborhood, computed for a given cell-type pair across all Xenium samples profiled with the Breast, Custom IO and 5 K panels. Dot size indicates the number of samples in which the cosine similarity was statistically significant, and color denotes the average value. The adjacent bar plot summarizes these values per secondary (that is, contaminating) cell type, representing the overall cell-type spillover index. **b**, Cosine similarity between cell’s secondary cell-type weight (w_2_) and the proportion of the secondary cell type in each cell’s neighborhood, computed for all pairs of primary and secondary cell types across replicates. Only statistically significant values are shown (p-value < 0.01, one-sided permutation test, n = 1000).

**Extended Data Fig. 6:**
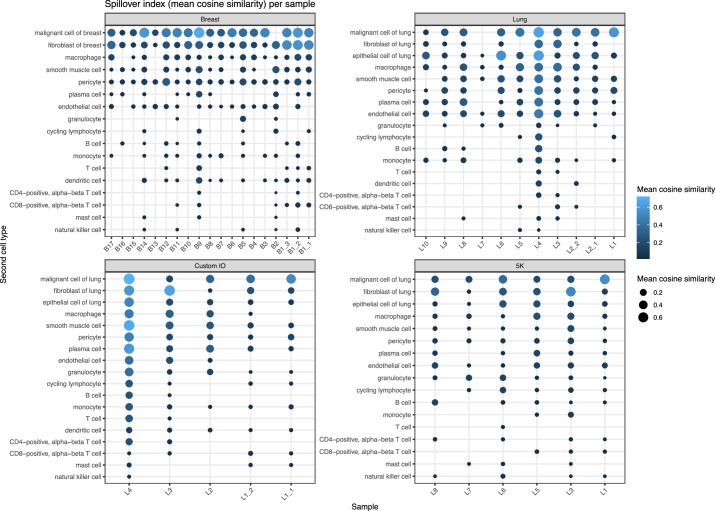
Cell-type spillover index per sample. The spillover index is defined as the mean cosine similarity between each cell’s secondary cell-type weight (w_2_) and the proportion of that same cell type in its spatial neighborhood, averaged across all primary cell types. When averaging, non-significant observations (p-value >= 0.01, one-sided permutation test, n = 1000) are set to zero.

To assess cell-type-specific contamination propensity, we defined a spillover index. For each pair of cell types, we calculated the cosine similarity between the contamination level of the primary (that is, affected) cell type (*w*_2_) and the local abundance of the secondary (that is, contaminating) cell type in its neighborhood. Averaging these pairwise scores across all primary cell types yields a spillover index that summarizes the tendency of each cell type to contribute contaminating signal (Fig. 3d, Extended Data Fig. 5 and 6, and Methods). More abundant cell types show higher spillover indices, with malignant cells ranking highest. Accordingly, we used malignant cells to illustrate tumor-driven contamination of neighboring cells. Figure 3e shows a clear association between malignant cell abundance and local contamination.

To further illustrate this effect and provide orthogonal validation, we performed multiplexed IHC on a subset of samples following Xenium analysis (Methods). As protein staining is less prone to spillover artifacts, IHC offers an independent modality to validate spatial transcriptomic signals and cell-type annotations. In particular, the IHC data allowed us to confidently identify CD8^+^ T cells (stained in pink) and malignant cells (stained in red). Consistent with our hypothesis of physical transcript spillover, Fig. 3a,f,g shows tumor-specific transcripts (small brown dots) extending into adjacent CD8^+^ T cells, supporting the presence of spillover contamination. In some instances, this contamination is sufficiently strong that RCTD confuses the primary and secondary cell types (Fig. 3f).

### Signal decomposition reduces spillover and enhances cell-type purity

To address spatial spillover and the more general issue of cell-level contamination, we introduce a computational strategy that alleviates these effects by decomposing mixed expression profiles (Methods). Our approach leverages RCTD and cellular decomposition to separate mixed signals into cell-type-specific components, thereby minimizing contributions from secondary cell types. Specifically, RCTD is used to model each cell’s expression profile as a weighted combination of reference cell-type profiles (Fig. 3h). These weights are then used to proportionally assign transcripts to their most likely sources—primary or secondary—based on cell-type decomposition (Methods).

**Extended Data Fig. 7:**
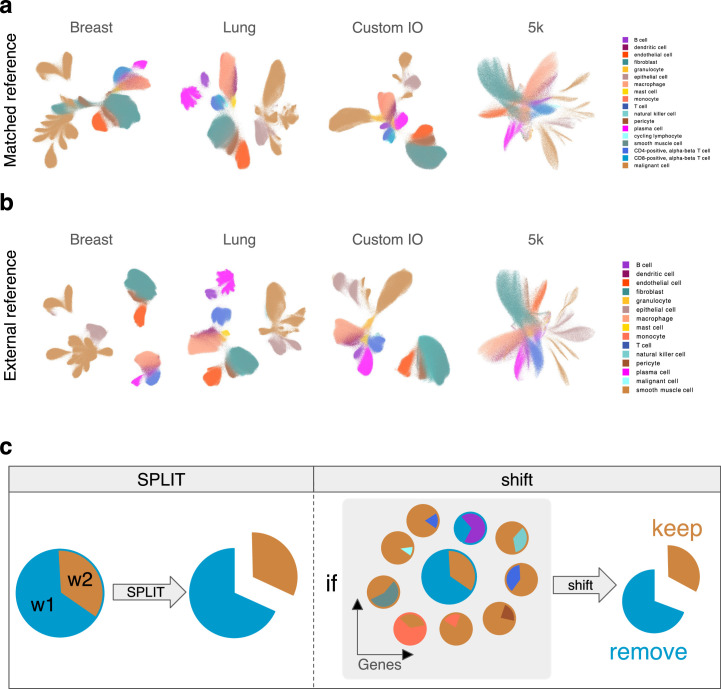
SPLIT. **a-b**, Integrated UMAPs of SPLIT-corrected cells using matched (**a**) and external (**b**) scRNA-seq data as reference, grouped by panel. **c**, Schematic of the SPLIT-shift procedure. SPLIT decomposes mixed transcriptomic profiles into purified primary and secondary components using reference profiles and assigned weights. By default, the primary profile is retained for downstream analysis. In SPLIT-shift, if there is evidence of a primary–secondary label swap—assessed based on the transcriptomic neighborhood being composed predominantly of the secondary cell type—the secondary profile is retained instead.

Using this strategy, we introduce SPLIT, a framework that separates mixed signals by splitting each cell according to its inferred cellular composition. UMAP embeddings of SPLIT-corrected cells (Extended Data Fig. 7a,b) show improved clustering and separation compared to the original mixed signals, consistent across all panels and for both matched and external references. Quantitative validation of these improvements is provided in the following section.

**Extended Data Fig. 8:**
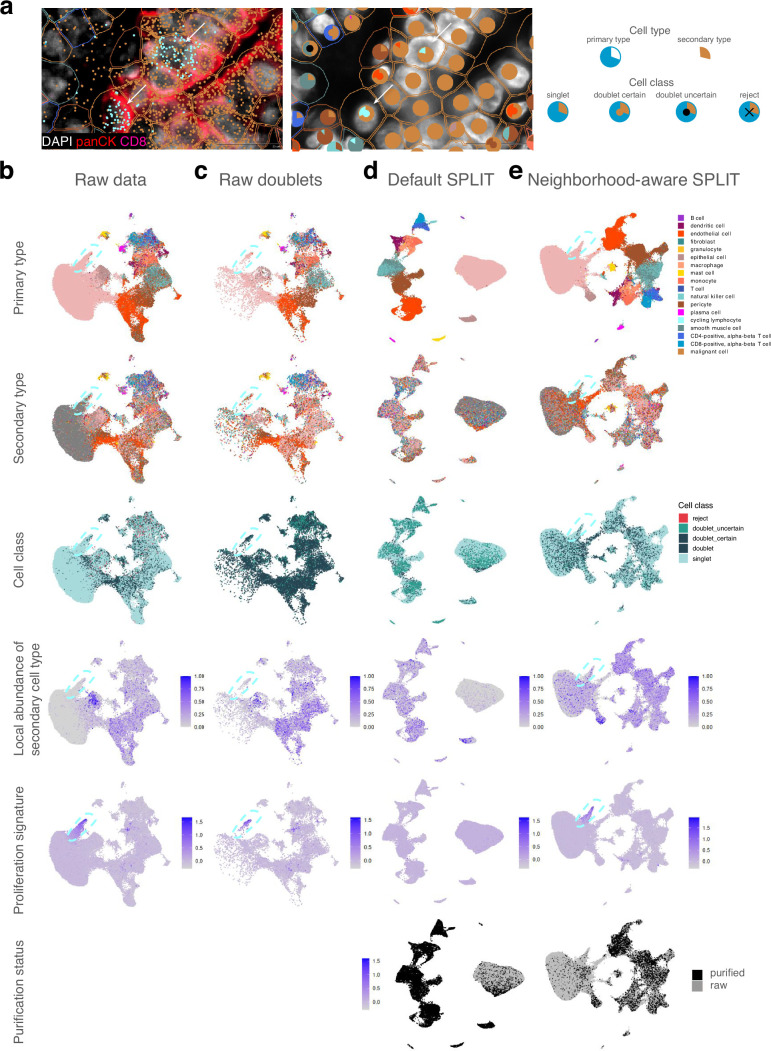
Doublets and specific phenotypes. **a**, IHC and morphology image highlighting cells identified as malignant (primary) with cycling lymphocytes (secondary). Cells are indicated by white arrows; contour color reflects primary RCTD annotation. Dots represent cell type–specific transcripts (tumor: brown; cycling lymphocytes: cyan). **b–e**, UMAPs of the same Xenium sample: (**b**) computed on raw data; (**c**) subset to doublets; (**d**) computed on data purified using the default SPLIT; (**e**) computed on data purified with the neighborhood-aware SPLIT. All maps are colored by the primary cell type (first row), secondary cell type (second row), cell class (third row), local abundance of the secondary cell type (fourth row), proliferation signature (fifth row), and purification status (sixth row).

By default, SPLIT decomposes all contaminated cells by preserving the expression profile of the primary cell type (Fig. 3h). In addition, SPLIT can leverage spatial information to assess the abundance of secondary signals in the local neighborhood (that is, local spillover potential), enabling selective decomposition only when contamination is likely and preventing overcorrection of phenotypes that may be underrepresented or absent in the reference. For instance, cycling tumor cells—missing from our reference but present in some matching Xenium samples—were often classified as malignant cells contaminated by cycling lymphocytes. SPLIT avoids decomposing such cells, preserving distinct and biologically relevant populations (Extended Data Fig. 8).

Beyond profile purification, SPLIT also enables phenotype refinement by shifting the primary phenotype assignment to one of the secondary phenotypes, based on transcriptional neighborhood homogeneity. We refer to this process as SPLIT-shift (Extended Data Fig. 7c).

### SPLIT compares favorably to other correction methods

Next, we evaluated SPLIT’s performance relative to other correction methods, namely ResolVI^14^ and ovrlpy^15^ as well as to various segmentation approaches. Some of these segmentation methods (that is, ProSeg) also directly model transcript spillover. Because SPLIT and other signal enhancement techniques can be combined with any segmentation method, we assessed all combinations in our comparison.

**Fig. 4:**
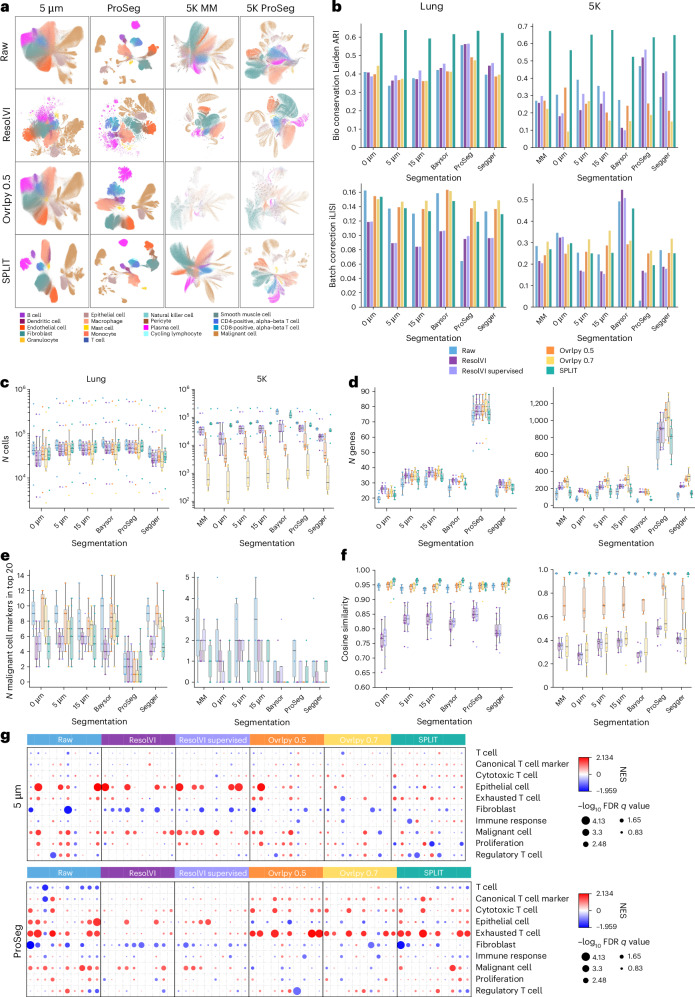
SPLIT improvements and comparison of segmentation approaches. **a**, UMAPs of Xenium cell-level data obtained before (raw) and after count correction for the Lung and 5K panels, and default (that is, 5 μm for the Lung panel and multimodal (MM) 5 μm for the 5K panel) or ProSeg segmentation. Colored by cell-type annotation. **b**–**f**, Metrics obtained before (raw) and after count correction for different segmentations and count correction methods, for Lung and 5K panel: biological conservation and batch correction metrics from SCIB (higher is better) (**b**); number of cells (**c**); median number of genes (**d**); strength of T cell contamination by malignant cells (**e**). Assessed by the number of malignant marker genes (with *N* = 20 markers) found in the top 20 ranks of sorted logistic regression coefficients. **f**, Cosine similarity of Xenium with Chromium snRNA-seq T cell pseudo-bulk profiles. All box plots show the median and interquartile range (IQR), with whiskers extending to the nearest value outside 1.5× IQR. Lung (left): *n* = 11 samples (10 biological and 2 technical replicates); 5K (right): *n* = 6 biological replicates. **g**, Dot plots showing normalized enrichment scores (NES) and −log_10_ FDR for Prerank GSEA ran using differential expression log fold changes for T cells close versus distant from malignant cells. Rows are individual samples. Top: Lung panel with 5 μm segmentation. Bottom: Lung panel with ProSeg segmentation.

Visual inspection of UMAPs shows that SPLIT improves cell-type separation (Fig. 4a). To quantitatively evaluate performance, we compared each correction method across different segmentation outputs based on cell type separation, batch integration and the retention of cells and transcripts. An optimal correction method should enhance separation between cell types, avoid introducing or amplifying batch effects, and preserve as many cells and transcripts as possible (Fig. 4b–d). Although not the primary focus of our study, these same metrics can also inform comparisons between segmentation algorithms.

In addition, to assess transcript spillover, we adopted a previously described^16^ neighborhood-based logistic-regression approach that tests whether gene expression in a given cell type predicts proximity to a potential contaminating cell type. Our analysis focused on malignant cells and T cells: malignant cells have larger spillover index and tend to release large amounts of RNA, while T cells are smaller and contain fewer transcripts, and are thus more vulnerable to contamination (Fig. 3d–g). We had access to IHC data for both cell types, which enabled visual inspection of the results.

Overall, all correction methods improve cell-type separation (Fig. 4b, top row) and decrease contamination (Fig. 4e) across all segmentation methods. We further observe that nucleus segmentation (0 µm), sometimes assumed to be contamination-free^19^, still shows signs of contamination which are improved by count correction (Fig. 4e). Samples profiled with the 5K panel displayed much lower contamination compared with other gene panels, especially when using multimodal segmentation (Fig. 4e). However, this reduction may be attributed to decreased statistical power resulting from a lower number of transcripts detected per gene. Batch effects are overall unchanged by correction methods, except for ResolVI, which sometimes results in slightly worse iLISI (Fig. 4b, bottom row).

SPLIT achieves the best cell-type separation (Fig. 4a,b, top row) and the highest cosine similarity with Chromium snRNA-seq profiles (Fig. 4f). While this is expected given that SPLIT relies on Chromium reference to resolve mixed cell-type signals, these results confirm its effectiveness in practice. While all correction methods reduce contamination (Fig. 4e), ovrlpy and ResolVI substantially decrease the number of expressed genes, resulting in many empty or near-empty cells (Fig. 4c). Once these low-expression cells are removed, the remaining cells express a higher median number of genes compared to raw segmentation data (Fig. 4d).

Our data further demonstrate that segmentation algorithms strongly impact cell-level transcript quantification. ProSeg yields a higher median gene count, including many low-expression values, probably reflecting its probabilistic assignment of nonzero expression genes that may otherwise be undetected. Overall, ProSeg outperforms Baysor, Multimodal and Segger (Fig. 4b), and combining ProSeg with SPLIT yields the greatest overall improvement, demonstrating that SPLIT can be combined with any segmentation tool.

**Extended Data Fig. 9:**
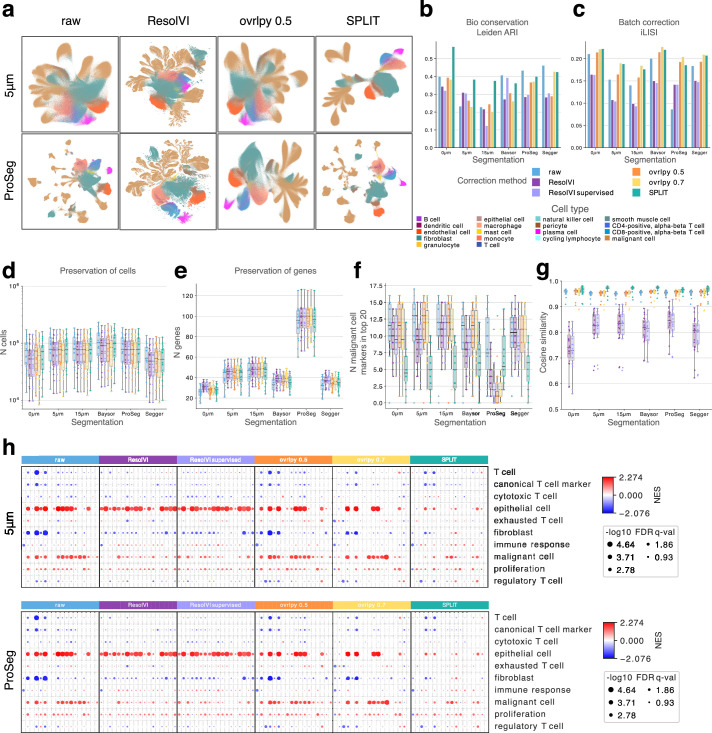
SPLIT improvements and comparison of segmentation approaches for Breast panel. **a**, UMAPs of Xenium cell-level data obtained before (raw) and after count correction for Breast panel and default (5 μm) or ProSeg segmentation. Colored by cell-type annotation. **b-g**, Show metrics obtained before (raw) and after count correction for different segmentations and count correction methods for Breast panel. **b**, Biological conservation and c, batch correction metrics from SCIB (higher is better). **d**, Number of cells. **e**, Median number of genes. **f**, Strength of T cell contamination by malignant cells. Assessed by the number of malignant marker genes (with N = 20 markers) found in the top 20 ranks of sorted logistic regression coefficients. **g**, Cosine similarity of Xenium with Chromium snRNA-seq T cell pseudo-bulk profiles. **h**, Dot plots showing Normalized Enrichment Scores (NES) and -log10 FDR for Prerank GSEA ran using differential expression log-fold changes for T cells close vs distant from malignant cells. Rows are individual samples. Top: Breast panel with 5 μm segmentation; Bottom: Breast panel with ProSeg segmentation. All box plots show the median and interquartile range (IQR), with whiskers extending to the nearest value outside 1.5× IQR; n = 19 samples (17 biological and 3 technical replicates).

While our comparative analysis primarily focuses on lung samples—owing to the availability of multiple gene panels—similar analyses in breast cancer samples yielded consistent conclusions (Extended Data Fig. 9).

### T cells near malignant cells exhibit increased exhaustion signatures

To further demonstrate SPLIT’s utility for biological insight, we examined phenotypic differences between T cells located near malignant cells and those positioned farther away in lung tissues, both before and after correction. In the default 5 μm segmentation—and to a lesser extent with ProSeg—T cells adjacent to tumor regions exhibited spurious enrichment of malignant and epithelial gene signatures in the raw data (Fig. 4g). While methods like ResolVI and Ovrlpy reduced this contamination and partially recovered T cell signatures, their effects were inconsistent; notably, ResolVI occasionally suppressed both contaminating and T cell programs. By contrast, SPLIT consistently removed nonspecific signals while preserving or enhancing T cell signatures across segmentations.

**Extended Data Fig. 10:**
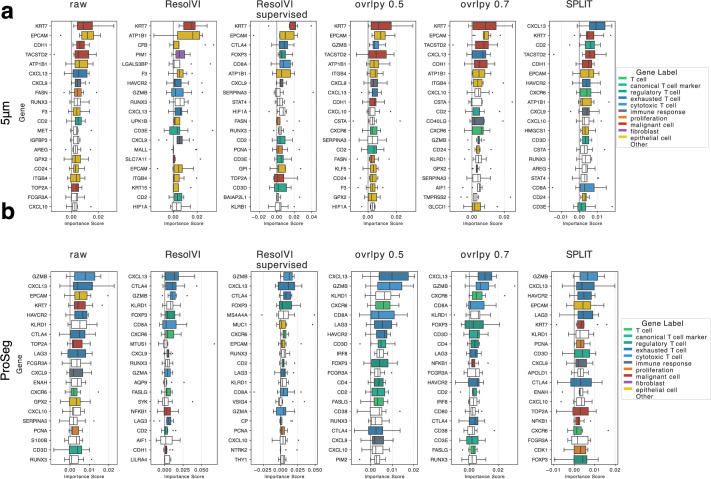
Top predictive genes for T cell proximity to malignancy across segmentations. Top 20 gene importances for logistic regression models trained to predict T cells located near versus distant from malignant cells using the lung gene panel. Gene importances are ranked by their mean across samples for each count correction method. **a**, Results using 5 µm segmentation. **b**, Results using ProSeg segmentation. Box plots show the median and interquartile range (IQR), with whiskers extending to the nearest value outside 1.5× IQR; n = 11 samples (10 biological and 2 technical replicates).

Following SPLIT correction, T cells near tumor cells showed stronger exhaustion signatures, including increased expression of canonical markers such as *HAVCR2* (TIM-3), *CTLA4*, *PDCD1*, *LAG3* and *CXCL13* (Extended Data Fig. 10), in line with prior studies^19,28^. For instance, Jones et al.^19^ reported *CXCL13* enrichment in T cells adjacent to malignant cells after ProSeg-based segmentation in renal cell carcinoma. Our results not only corroborate this finding but also highlight SPLIT’s added value, with greater enrichment of exhaustion markers and clearer removal of non-T-cell contamination. Combining ProSeg with SPLIT yielded further improvements, emphasizing their complementarity.

## Discussion

In this study, we leveraged an extensive Xenium spatial transcriptomics dataset to assess data quality, evaluate segmentation and correction strategies, and introduce SPLIT, a novel method for signal purification. Our analyses confirm that Xenium produces high-quality, highly reproducible data with low technical variation, enabling reliable integration across tissue sections and patients and offering strong potential for both single-sample and multisample spatial studies. Several of our findings were validated using additional publicly available Xenium dataset, supporting their generalizability. However, as our primary analyses were limited to two cancer types, further validation across additional datasets and tumor contexts will be required.

Our comparison of panel designs highlights that targeted panels, despite covering fewer genes, offer greater sensitivity and are sufficient to resolve major cell types. By contrast, the 5K panel offers broader coverage but reduced per-gene sensitivity and weaker downstream performance. This trade-off suggests opportunities to optimize probe composition by fine-tuning panel content or supplementing the 5K panel with additional targets, as recommended by 10x Genomics and supported by recent computational advances in probe^29^. As spatial transcriptomics scales to larger discovery studies, systematically quantifying this sensitivity–coverage trade-off and defining criteria for optimal panel design will be essential, particularly as reduced sensitivity may be acceptable in exploratory settings.

We also confirm previous reports that single-cell expression data derived from Xenium are prone to transcript contamination, stemming from segmentation inaccuracies and transcript spillover—two interrelated sources of error. Using complementary RCTD deconvolution and IHC data, we showed that this artifact is widespread and disproportionately affects low RNA-content cells, such as T cells. As noted by Mitchel et al.^16^, such contamination can substantially distort the inference of cellular niches—one of the key goals of spatial transcriptomics. These findings underscore the importance of developing robust computational strategies to correct for contamination and highlight the need for researchers to critically evaluate the outputs of cell–cell interaction inference tools. This concern is particularly relevant for emerging foundation models trained on large-scale spatial transcriptomics datasets, where self-supervised tasks often involve predicting masked cell phenotypes based on spatial context^30,31^. Uncorrected contamination may introduce systematic biases that propagate through downstream predictions, underscoring the need for caution when interpreting model outputs.

We demonstrated that incorporating snRNA-seq as a reference markedly enhances cell-type annotation. In addition, RCTD’s doublet model reveals the composition of mixed signals by estimating the contributing cell types and their relative proportions. We further leverage this model within SPLIT to decompose mixed signals and correct for transcript spillover, thereby improving cell-type resolution. While matched references from the same sample are preferable—particularly for distinguishing malignant populations—we show that external references also perform well, underscoring the robustness and generalizability of reference-based approaches. With the growing availability of reference datasets from initiatives such as the Human Cell Atlas^32^, we anticipate that reference-based analyses will become increasingly accessible. Nonetheless, future work will focus on developing reference-free versions of SPLIT to further expand its applicability.

We also evaluated the performance and implications of background correction and segmentation strategies, including our method, SPLIT. While many correction techniques can be applied independently of the segmentation algorithm, they often raise concerns about interpretability and the risk of overcorrection—particularly when used in combination with transcript-space segmentation methods like ProSeg, which already account for spillover. This is especially evident with approaches such as Ovrlpy and ResolVI, which can substantially reduce gene counts and, as a result, may compromise statistical power. By contrast, SPLIT is less prone to these artifacts, as it operates as a reference-based signal decomposition method rather than a direct spatial correction layer. Its outputs are inherently more interpretable, and importantly, SPLIT preserves gene detection levels more effectively than alternative methods.

Segmentation in transcript space—particularly using probabilistic methods such as ProSeg—improves cell-type resolution, especially when paired with targeted panels. However, these gains are more modest with the 5K panel, probably due to its lower sensitivity and the increased complexity of performing segmentation in a higher-dimensional space. Nevertheless, transcript-based segmentation remains fully compatible with correction methods like SPLIT, allowing them to be effectively combined for enhanced resolution. Notably, applying SPLIT to the 5K panel improved cell-type separation when using the default 5-μm expansion, highlighting its utility even under standard segmentation conditions. This advantage was further evident in our analysis of T cells proximal to malignant regions, where SPLIT markedly enhanced detection of exhaustion signatures following correction.

An important consideration when using SPLIT is the interpretation of mixed cellular phenotypes inferred from deconvolution, as substantial contamination—particularly in low–RNA content cells such as T cells—can lead to misclassification of the true phenotype as secondary. This general limitation of deconvolution-based approaches motivates strategies to better resolve mixed signals. In the current implementation, we retain the primary phenotype but optionally allow phenotype shifts through a simple procedure we refer to as SPLIT-shift, while future extensions could incorporate more advanced criteria, including morphological features, to infer true cellular identity.

Although originally developed for RCTD, SPLIT is now deconvolution-agnostic, supports generic deconvolution outputs and enables alternative strategies for handling contaminating transcripts, including reassignment to neighboring cells of the appropriate type, to be tested in future work.

It is also important to recognize that both reference-based cell-type annotation and SPLIT assume that all relevant cell types are present in the reference. The absence of phenotypes can compromise annotation, potentially resulting in artificial contaminated assignments as the model attempts to account for unrepresented variation. For instance, cycling tumor cells may be incorrectly decomposed into a combination of cycling T cells and noncycling tumor cells. This represents a critical caveat that users should consider when interpreting annotation and decomposition results.

## Methods

### Human samples

All patients provided informed consent for the use of the tumor samples in this study. The protocol used for sample collection was approved by the local ethics committee (CER-VD, BASEC ID 2016-02094).

### Datasets

#### Study design

Data were generated from 10 FFPE NSCLC samples and 17 FFPE breast cancer samples. Xenium spatial transcriptomics was performed on all samples using different gene panels, with the 5K panel specifically applied to lung samples. In addition, matched snRNA-seq and multiplexed IHC data were generated for selected samples, as detailed below and in Fig. 1.

#### Xenium

Xenium lung tissues were processed using three gene panels in parallel, the 10x Human Lung panel, a custom immuno-oncology panel (Custom IO; see Supplementary Table 2 for the gene list) and the 10x Prime 5K Human Pan Tissue & Pathways panel, allowing an investigation into how the choice of a gene panel may affect downstream analyses. For breast samples, we used the 10x Human Breast panel.

#### snRNA-seq

snRNA-seq data were obtained from FFPE blocks from eight patients, four per disease group, on the Chromium platform with NEXT-GEM FLEX chemistry. Details about this dataset and its methodology are available in the publication by Dong et al.^2^.

#### IHC

Two post-Xenium slides—one per disease—containing a total of five tissue sections were immunohistochemically stained for 4′,6-diamidino-2-phenylindole (DAPI; Spectral DAPI, AKOYA), CD8 (Cellmarque, Clone SP16, 108R) and pan-cytokeratin (panCK, Dako, Clone AE1/AE3, IS053) in an autostainer (ventana Discovery Ultra, Roche) using the Tyramide Signal Amplification system (OPAL, Akoya), allowing us to identify CD8^+^ T cells and malignant cells. Antigenicity was reduced in some samples, particularly those processed with the multimodal staining protocol used for Xenium segmentation, leading to failed IHC staining in certain cases. As a result, IHC yielded usable staining quality in only five samples.

### Data preprocessing and QC

#### snRNA-seq

Filtered barcode counts from CellRanger were analyzed using the Seurat package^33^ (v. 5.0.1) in R (v. 4.3.2, https://www.R-project.org/). Cells with at least 200 detected genes and fewer than 20% of reads mapping to mitochondrial genes were retained. To recover neutrophils, the gene threshold was lowered to 100 genes per cell, and cells forming a distinct cluster with high expression of canonical neutrophil markers (that is, *FCGR3B*, *S100A9*, *IL1R2*, *CSF3R*, *FPR1* and *NAMPT*) were retained.

Raw counts were normalized using SCTransform, and the top 3,000 variable genes across samples were selected using SelectIntegrationFeatures. Dimensionality reduction was done using principal component analysis (PCA). Clustering was done via Seurat’s shared nearest neighbor modularity optimization algorithm (FindNeighbors and FindClusters) using 30 principal components and resolutions between 0.4 and 0.8.

Clusters were annotated based on the top differentially expressed genes and canonical markers. Significant markers were identified with Seurat::FindAllMarkers() (two-sided Wilcoxon’s rank sum test, Bonferroni correction, adjusted *P* < 0.05, log fold change >1). Subclustering enabled finer annotation, resulting in four hierarchical annotation levels. Cell-type labels were standardized to match ontology naming conventions. This dataset was used as a matched reference for RCTD cell-type annotation of Xenium data.

Annotation Level 2.1 was added for visualizing cell types on Xenium. As an extension of the broader Level 2, it distinguishes CD8^+^ T cells and supports validation with IHC staining.

#### Xenium

##### 10x segmentation

To facilitate segmentation, Xenium’s standard output includes a DAPI-stained image for nuclear localization along with the *x*, *y* and *z* coordinates of individual transcripts. Segmentation algorithms can leverage this information to assign transcripts to individual cells. Unless the multimodal segmentation kit is used, the default 10x segmentation algorithm expands from the nucleus outward—up to 5 µm or until it reaches the boundary of an adjacent cell.

In our study, the multimodal segmentation kit was applied only to the 5K panel. Therefore, we used the default 5-µm nuclear expansion-based segmentation for all other panels. In practice, segmentation results are identical for a substantial proportion of cells, as the multimodal method defaults to the 5-µm nuclear expansion when membrane staining is weak or absent. In either case, we consider these approaches to provide reasonable and consistent baselines for our analyses.

##### Alternative segmentations

Several alternative segmentation methods have been developed to improve upon the default 10x approach described above. In this study, we evaluated Baysor (v0.7.0, https://github.com/kharchenkolab/Baysor), ProSeg (v2, https://github.com/dcjones/proseg, commit ef2d1ca8c535fd911b7cd37da47c84de98e784a4) and Segger (a version adapted by us at https://github.com/bdsc-tds/segger_dev, commit 4bf56dec2a364de8eee4fcab663e798eb106e21a)—all of which utilize transcript-level information to guide segmentation. We applied these methods with default arguments to all Xenium samples. Specifically, when running Segger, we used 6,000 tokens for samples from the 5K panel and 500 tokens for others. These methods represent robust alternatives and serve as valuable baselines for benchmarking segmentation performance.

##### Quality control

Cells with fewer than ten counts or five genes were filtered out, as well as genes expressed in fewer than ten cells.

#### IHC

##### Analysis

Xenium and IHC images were coregistered using Xenium Explorer (v3.2.0). Xenium’s cell segmentation, as a GeoJSON file, was loaded into a project in Qupath (v0.5.1) with the corresponding IHC image (details included as part of our pipeline; see ‘Code availability’ section), and an object classifier was trained within QuPath to classify cells based on protein expression.

### Downstream analyses

#### UMAP visualization

UMAPs were computed for log-normalized snRNA-seq and for Xenium data at the sample and panel level using default scanpy^34^ parameters for PCA, *k*-nearest neighbor (kNN) search and UMAP, except kNN n_neighbors, which was set to 50.

#### RCTD cell-type decomposition

RCTD^22^ is a computational method for deconvolving spatial transcriptomics data using single-cell RNA-seq references. It uses a Poisson regression model to represent each spatial unit (spot or segmented cell) as a mixture of cell-type-specific profiles, assigning weights that capture the contribution of each type. In doublet mode, RCTD restricts the decomposition to two cell types, assigning *w*_1_ to the primary type and *w*_2_ to the secondary type. In our analysis, we interpret the primary cell type as the cell’s true underlying identity, whereas the secondary type and its weight *w*_2_ are treated as indicative of contaminating signal and its magnitude.

In addition to cell-type composition, RCTD in doublet mode assigns a ‘spot class’ to each unit, with four possible values: (1) singlet, for cells confidently assigned to a single type; (2) doublet_certain, for cells confidently annotated as a mixture of two types; (3) doublet_uncertain, for cells with a confident primary type but unclear contamination sources; and (4) rejects, for cells with uncertain annotation. This classification is based on the difference between two scores: the singlet score, the residual error between the observed profile and the primary reference profile, and the doublet score, the residual error to the weighted sum (*w*_1_ and *w*_2_) of primary and secondary profiles. This doublet–singlet calling is based on the difference between two scores: singlet score and doublet score. The doublet calling is performed on a fixed threshold. Because residuals accumulate over all expressed genes, the score difference tends to be higher in larger panels (for example, 5K Prime), leading to higher apparent doublet rates. Therefore, doublet scores and rates are only meaningful for comparisons within the same panel.

#### Xenium annotation with RCTD

We performed cell-type annotation of Xenium data using the RCTD algorithm in doublet mode, leveraging both matched snRNA-seq and external scRNA-seq references (see ‘Data availability’ section). Each sample was annotated independently. To enhance the specificity of tumor cell detection, matched references were restricted to include only tumor cells derived from the corresponding donor, when donor-specific cells were available. Reference datasets were filtered to remove cells with fewer than 10 or more than 2,000 unique molecular identifiers (UMIs), and cell types represented by fewer than 25 cells were excluded. RCTD was run on raw UMI count data from both the reference and Xenium data. Due to the inherently lower UMI counts in single-cell resolution Xenium data, we applied a more permissive filtering strategy: cells with fewer than 10 total UMIs were excluded and do not appear in further plots, and the UMI_min_sigma parameter was reduced to 100 to account for the reduced transcript coverage compared with spot-based spatial transcriptomics in the original RCTD application. To improve the robustness of RCTD annotation, we provided the class_df parameter, which groups cell types into broader classes. Finally, we applied mild postprocessing to the RCTD results, removing an arbitrary second cell type in highly confident singlets—specifically, singlets that showed no evidence of a secondary signal.

#### Spatial spillover and contamination metrics

A cell’s neighborhood composition is defined as the relative proportion of cell types within a cell’s spatial neighborhood. The spatial neighborhood consists of up to 20 nearest cells within a 15-µm radius. Each neighbor is represented by two cell types (primary and secondary) with associated weights defined above (namely *w*_1_ and *w*_2_), obtained from the RCTD decomposition. Within a neighborhood, weights are aggregated by cell type and normalized to sum to 1, yielding a vector of relative cell-type proportions, referred to as the neighborhood composition.

The proportion of the secondary cell type in the neighborhood is defined as the neighborhood composition weight corresponding to the secondary cell type. Equivalently, it can be described as the average weight of the signal corresponding to a given cell’s secondary cell type across all other cells in its spatial neighborhood.

The pairwise spillover index is defined as the cosine similarity between a cell’s secondary cell-type weight (*w*_2_) and the proportion of that same cell type within the cell’s spatial neighborhood, computed across all cells sharing a specific primary and secondary cell-type pair. A higher index suggests greater evidence of spillover from type 2 into type 1. For each sample, a pairwise spillover index was computed for cell-type pairs having at least 20 observations. Nonsignificant scores (<20 observations or permutation test false discovery rate (FDR) >0.01) were replaced with zeros.

The cell-type spillover index is derived by averaging the pairwise spillover indices across all primary cell types for each given secondary (contaminating) cell type. A higher index indicates stronger spillover originating from that secondary cell type. When averaging across all primary cell types, statistically nonsignificant observations (<20 observations or permutation test FDR >0.01) were treated as zeros.

#### SPLIT correction

To reduce contamination from secondary cell-type signals in an observed expression profile, we leveraged RCTD output. RCTD provides, for each cell, the primary and secondary cell-type labels, along with their weights *w*_1_ and *w*_2_ = 1 − *w*_1_, which reflect the relative contributions of the primary and secondary reference profiles to the observed expression. Using these weights and the reference profiles, we computed a purified expression profile designed to separate the contribution of the primary cell type from the secondary cell type. Specifically, for each cell:

$${{\bf{x}}}_{\mathrm{doublet}-\mathrm{SPLIT}}=\frac{{w}_{1}{{\bf{ref}}}_{1}}{{w}_{1}{{\bf{ref}}}_{1}+{w}_{2}{{\bf{ref}}}_{2}}\odot {{\bf{x}}}_{\mathrm{observed}},$$

where **ref**_1_ and **ref**_2_ are the average reference profiles of the primary and secondary cell types, respectively. Fraction bar and ‘$\odot$’ correspond to element-wise division and product, respectively. Intuitively, this ratio (used as a scaling factor) is an estimate of the expected fraction of transcripts coming from the primary cell type. The complement, namely $\frac{{w}_{2}{{\bf{ref}}}_{2}}{{w}_{1}{{\bf{ref}}}_{1}+{w}_{2}{{\bf{ref}}}_{2}}$, being the expected fraction of transcripts coming from the secondary cell type.

We call this approach doublet-SPLIT and by default apply it to cells classified as ‘doublets_certain’ and ‘singlets’ that showed signs of contamination—that is, cells for which both a primary and secondary cell type were confidently assigned by RCTD.

For cells labeled as ‘doublets_uncertain’, only the primary cell type is confidently assigned, and the secondary identity is considered ambiguous. To purify these cells, we performed a full-SPLIT as

$${{\bf{x}}}_{\mathrm{full}-\mathrm{SPLIT}}=\frac{{w}_{1}{{\bf{ref}}}_{1}}{{\sum }_{i=1}^{k}{w}_{i}\,{{\bf{ref}}}_{i}}\odot {{\bf{x}}}_{\mathrm{observed}}.$$

In addition, we observed that RCTD occasionally swaps the primary and secondary cell types. To account for this, we implemented a label-correction step termed SPLIT-shift. If a cell has primary label *ct*_1_ and secondary label *ct*_2_, but its transcriptomic neighborhood is primarily composed of *ct*_2_ cells, we assume a potential label swap and perform purification using *ct*_2_ as the primary identity, namely

$${{\bf{x}}}_{\mathrm{SPLIT}-\mathrm{shift}}=\frac{{w}_{2}{{\bf{ref}}}_{2}}{{w}_{1}{{\bf{ref}}}_{1}+{w}_{2}{{\bf{ref}}}_{2}}\odot {{\bf{x}}}_{\mathrm{observed}}.$$

To support SPLIT-shift decisions, we also constructed a transcriptomic neighborhood for each cell, defined by a kNN graph (*k* = 10) based on Euclidean distance in PCA space.

#### Which cells to SPLIT

We offer several modes to decide which cells should be purified:

- Default: purifies all cells having secondary cell type by applying doublet-SPLIT to singlets and doublets_certain, and full-SPLIT to doublets_uncertain.
- balance_score_based: singlets and doublets_certain undergo doublet-SPLIT only if they show evidence of contamination, specifically, if they are surrounded by cells of their assigned secondary type. Doublets_uncertain still receive full-SPLIT; all others remain unchanged. To support this decision-making, we introduce a spatial neighborhood-based metric, neighborhood_weight_second_type (referred to as proportion of secondary cell type in cell’s neighborhood in Fig. 3), which represents the average proportion of a cell’s secondary type found within its local spatial neighborhood. In this study, we defined each cell’s spatial neighborhood using a kNN approach with (*k* = 20) based on Euclidean distance in physical space. To ensure locality, we removed all edges longer than 15 µm.

#### Alternative count correction

ResolVI^14^ is a probabilistic model that refines spatial transcriptomic data by resolving cell-type mixtures using variational inference. In this study, we applied ResolVI using default parameters, with models trained either in an unsupervised manner or supervised using Level 2.1 cell-type labels, with T cell subtypes and malignant cell subtypes each grouped into one category. We treated sample information as a batch covariate, as recommended through personal communication with the authors (https://github.com/bdsc-tds/xenium_analysis_pipeline/issues/83). Because ResolVI requires input expression values to be integers, ProSeg expression matrices were rounded to the nearest integer.

Ovrlpy^15^ combines a vertical subslicing strategy with an unsupervised, segmentation-free analysis of spatial transcriptomics data to identify and correct for potential spatial doublets. In this study, we applied ovrlpy with default parameters, with a threshold of either 0.5 or 0.7 applied to the pixel signal integrity map, as recommended in the original preprint and through personal communication with the authors (https://github.com/HiDiHlabs/ovrl.py/issues/40), respectively.

### Comparing segmentations and count correction methods

Computational procedures and metrics for assessing cell type separation, batch effect, data quality and potential spatial signal contamination. Analyses focused on quantifying specificity (that is, potential contamination between neighboring cell types) and sensitivity (that is, data preservation, similarity to chromium profiles) were adapted from Mitchel et al.^16^. These were applied to each sample, before and after count correction.

### Assessment of cell-type separation and batch effect strength

Biological conservation of cell types and batch effect strength were assessed using metrics proposed by Luecken et al.^26^, as implemented in the scib-metrics package (https://github.com/YosefLab/scib-metrics) and Calinski–Harabasz, Davies–Bouldin scores from scikit-learn^35^. Higher scores indicate improved biological conservation or batch integration; Extended Data Fig. 2 uses color coding to facilitate interpretation and comparison across methods. Malignant cells were excluded before computing these metrics because they are often sample specific. Datasets were downsampled to a maximum of 100,000 cells to speed up calculations.

### Assessment of specificity and potential contamination

To evaluate potential transcript spillover or misassignment between spatially adjacent cells of different types, we reimplemented a recently proposed approach^16^. For each pair of distinct cell types, denoted as the target cell type *i* and the potential contaminating cell type *j*, cells annotated as type *i* were assigned a binary label: 1 for those spatially adjacent (based on centroid distance ≤15 µm) to at least one cell of type *j*, and 0 for those not adjacent to any cell of type *j*. We then trained a logistic regression model to predict, based on the gene expression profile of a cell of type *i*, whether it is adjacent to a cell of type *j*.

For each sample after QC filtering, input features were *z*-scored log-normalized expression levels of all genes for cells of type *i*. We used the implementation from scikit-learn^35^ with default parameters, except for max_iter, which was increased to 500 and *class_weight* set to ‘balanced’ to account for possible imbalance of target labels.

Genes were ranked by the magnitude of their coefficients to assess their potential association with signal contamination from cell type *j* within cell type *i*. To evaluate whether these ranked gene lists were enriched for markers of cell type *j*, we counted the number of markers present in the top 20 ranks. Cell type markers were defined independently for each sample, as the top 20 genes identified via Wilcoxon rank-sum test statistics, comparing with all other cells.

This analysis was run across all segmentations of each Xenium sample, both before and after applying count correction. We used RCTD cell-type annotation Level 2.1, grouping T cell subtypes into one class and tumor subtypes into one class. Cell types with more than 50,000 cells were downsampled to a maximum of 25,000 cells from each class. For a given sample, cell types were excluded from analysis if fewer than 100 cells were available in both target classes (proximal versus distal to cell type *j*).

In addition, we ran Wilcoxon differential expression tests between classes, on all samples except those with fewer than 30 cells available in both target classes. For T cell contamination by malignant cells, we ran Prerank gene set enrichment analysis (GSEA) using genes ranked by log-fold changes and custom gene sets (Supplementary Tables 3 and 4).

### Assessment of sensitivity and data quality

After applying QC filters (see ‘Xenium’ in ‘Data preprocessing and QC’ section in Methods) to raw or corrected count matrices, standard QC metrics were computed per cell, including the total number of UMIs or reads detected, and the number of genes expressed (defined as genes with >0 counts). Mean and median values for the number of genes expressed per cell, as well as the mean UMI count per cell, were calculated across all cells.

To assess the biological fidelity of the expression profiles, we compared them with reference chromium data. Pseudo-bulk expression profiles were generated for each identified cell type in our dataset by summing gene expression across all cells assigned to that type. We then calculated the cosine similarity between log-normalized pseudo-bulk profiles and corresponding cell type profiles derived from Chromium scRNA-seq. The similarity calculation was performed using all shared genes.

#### Analysis coordination

Given the raw Xenium data and the curated snRNA-seq reference, we implemented a reproducible Snakemake^36^ pipeline to coordinate the above sophisticated analysis on a high-performance cluster. The number of threads and the amount of memory for each task were allocated dynamically, with a limit of 48 threads and 1 TB memory, and the run time was limited to 3 days (72 h). Tasks that failed due to out-of-memory errors were excluded from the results.

### Statistics and reproducibility

All correlations were quantified using Spearman’s rank correlation coefficient (*R*). Statistical significance was assessed using a two-sided *t*-test of the null hypothesis *R* = 0.

Statistical significance of cosine similarity metric was assessed using a one-sided permutation test (*n* = 1,000).

The micrographs shown in Fig. 3f,g are illustrative. Confounding of primary and contaminating cell-type assignments by RCTD, occurring when contaminating signal dominates the true signal, was observed consistently within each slide and across all five slides with successful IHC staining.

### Reporting summary

Further information on research design is available in the Nature Portfolio Reporting Summary linked to this article.

## Online content

Any methods, additional references, Nature Portfolio reporting summaries, source data, extended data, supplementary information, acknowledgements, peer review information; details of author contributions and competing interests; and statements of data and code availability are available at 10.1038/s41592-026-03089-8.

## Supplementary information

Supplementary Information Supplementary Information.

Reporting Summary

Peer Review File

Supplementary Tables 1–4 Supplementary Table 1: Overview of Xenium samples. For each Xenium sample, the table includes panel information, the default segmentation used and whether matching snRNA-seq and IHC data are available. Supplementary Table 2: Custom IO panel genes. Supplementary Table 3: Annotated Lung panel genes. Supplementary Table 4: Annotated Breast panel genes.

## Data Availability

Xenium data produced as part of this study have been deposited in NCBI’s Gene Expression Omnibus (GEO) and are accessible through GEO series accession number GSE311609. Raw data of our matched lung and breast cancer snRNA-seq references are publicly available via cellxgene at https://cellxgene.cziscience.com/collections/bd552f76-1f1b-43a3-b9ee-0aace57e90d6. The external lung scRNA-seq reference is available via cellxgene at https://cellxgene.cziscience.com/collections/edb893ee-4066-4128-9aec-5eb2b03f8287. We used the extended atlas, containing data from 318 patients, which has over 1 million cells^37^. The external breast scRNA-seq reference described by Wu et al.^38^ is available at https://www.ncbi.nlm.nih.gov/geo/query/acc.cgi?acc=GSE176078.

## Code availability

Details on reproducing our analysis and analyzing other datasets are available via GitHub at https://github.com/bdsc-tds/xenium_analysis_pipeline. SPLIT is available as an R package, via GitHub at https://github.com/bdsc-tds/SPLIT. Code to reproduce the analysis and figures is available via GitHub at https://github.com/bdsc-tds/Bilous2026.
