# Supplementary material for: Resolving sensitivity, specificity and signal contamination in Xenium spatial transcriptomics
Source: Nat Methods. 2026 Apr 30;23(6):1152–62. doi: 10.1038/s41592-026-03089-8 (PMC13259927; doi:10.1038/s41592-026-03089-8)

nature methods

Supplementary information https://doi.org/10.1038/s41592-026-03089-8

Resolving sensitivity, specificity and signal contamination in Xenium spatial transcriptomics

In the format provided by the authors and unedited

Table of Content

Supplementary Notes

Supplementary Note 1. RCTD-identified doublets in Xenium data reflect transcript contamination rather than physical doublets

Supplementary Note 2. Nuclear segmentation provides a clearer transcriptomics profile.

Supplementary Note 3. Transcript spillover is reproducible in public datasets.

Supplementary Methods

Data availability

References

Supplementary Tables Legends/Captions

Supplementary Table 1. Overview of Xenium samples.

Supplementary Table 2. Custom IO panel genes.

Supplementary Table 3. Annotated Lung panel genes.

Supplementary Table 4. Annotated Breast panel genes.

Supplementary Figures Legends/Captions

Supplementary Fig. 1. Consistency of Xenium data across patients and technical replicates in breast samples.

Supplementary Fig. 2. Consistency of Xenium data across patients and technical replicates in lung samples.

Supplementary Fig. 3. Cell class composition across segmentations.

Supplementary Fig. 4. Comparison of cell-class composition obtained from scDblFinder and RCTD doublet detection.

Supplementary Fig. 5. Comparison of cell-class composition obtained from TACIT and RCTD deconvolutions.

Supplementary Fig. 6. Comparison of cell-type annotations obtained from TACIT and RCTD deconvolutions.

Supplementary Fig. 7. Features of Xenium cells of different RCTD-derived spot classes.

Supplementary Fig. 8. Comparison of RCTD-derived cell-class composition in Chromium and Xenium data.

Supplementary Fig. 9. Transcript spillover in public Xenium datasets and across segmentations.

Supplementary Figures

Supplementary Notes

Supplementary Note 1. RCTD-identified doublets in Xenium data reflect transcript contamination rather than physical doublets

In our Xenium data generated using targeted panels, transcripts are assigned to cells based on a computational tissue expansion radius, which extends cell boundaries by 5 µm beyond the segmented nucleus, or until another cell boundary is reached. This default setting, defined by 10x genomics, aims to capture the mRNA dispersed throughout the cell cytoplasm. For the 5K panel, transcripts are assigned to cells based on multimodal segmentation (see Methods). Based on our RCTD analyses, we find that the standard segmentations (based on nucleus expansion or multimodal staining) often result in the assignment of mixed transcriptional signals to individual cells, classified as "doublets" by RCTD (Supplementary Fig. 3a). This finding is consistent with results obtained using another deconvolution tool scDblFinder[40] (Supplementary Fig. 4), and even higher proportion of doublets was observed when applying TACIT[41] (Supplementary Fig. 5 and 6), another deconvolution tool developed for imaging data.

Importantly, Xenium cells classified by RCTD as "doublets" rarely represent true biological doublets (i.e., two cells segmented as one) but instead reflect mixed signals, also supported by the fact that "doublets" do not double in cell size (Supplementary Fig. 7a). Since the distinction between singlets and doublets is based on a fixed threshold, many RCTD-derived "singlets" also exhibit secondary signals, in particular from neighboring cells (Fig. 3g, Supplementary Fig. 7b), indicating that contamination can occur even in confidently annotated single-cell profiles.

Supplementary Note 2. Nuclear segmentation provides a clearer transcriptomics profile.

RCTD doublet classification relies on a fixed threshold applied to a score that scales with the number of detected genes. As a result, panels with broader gene coverage, such as 5K, tend to produce higher doublet scores (Supplementary Fig. 8a). Consequently, doublet rates are not directly comparable across panels but remain informative for within-panel comparisons.

The RDCT doublet rate is lower when transcript assignment is based on nuclear segmentation (0 µm; Supplementary Fig. 3b) or when RCTD is (artificially) applied to snRNA-seq data (Supplementary Fig. 8b, see Supplementary Methods), which experience little to no transcript spillover. In agreement with previous studies[12], we also observe better cell-type separation in nuclear segmentations (Extended Data Fig. 2d). Both observations suggest that these admixtures are due to segmentation errors or transcript spillover between neighboring cells.

Supplementary Note 3. Transcript spillover is reproducible in public datasets.

To test this, we assessed the correlation between contamination levels—estimated by the RCTD-assigned weight of the secondary cell type in each cell (w_2)—and the local relative abundance of that same cell type in the surrounding 2D neighborhood (Fig.

3a,b). As shown in Fig. 3c, these two metrics show strong cosine similarity, indicating that spatial patterns of elevated local concentrations of a given cell type align with increased contamination from that type, consistent with transcript spillover. The same trends were observed in independent, publicly available Xenium datasets derived from additional tissue types (Supplementary Fig. 9a,b). The relatively lower cosine similarity observed for the 5K panel may reflect improvements in the updated chemistry and more accurate multimodal (MM) segmentation that together reduce spillover (Supplementary Fig. 9c). However, this trend could also arise from the panel's lower per-gene RNA abundance, which may inherently limit detectable contamination, or from variation in RCTD decomposition due to the larger gene panel.

Supplementary Methods

Xenium annotation with TACIT

We performed an alternative cell-type deconvolution using TACIT[41], a method specifically developed for cell-type annotation and deconvolution in imaging-based spatial transcriptomics. Similar to RCTD, TACIT relies on a cell-type reference. We applied TACIT to our data in two modes: (i) *signature mode*, as recommended in the original publication for Xenium data, using the top 10 most enriched differentially expressed genes per cell type; and (ii) *reference mode*,for direct comparability with RCTD, using the same reference expression profiles. For all the other parameters,we used the values suggested in the vignette.

Despite extensive efforts to integrate TACIT into our automated processing pipeline, technical limitations required us to run it manually on a per-sample basis. Some runs failed to complete, and in several others, the algorithm did not converge. Nevertheless, we include all available TACIT outputs to provide the most comprehensive comparison possible across samples.

Doublet detection in Xenium with scDblFinder

We would like to emphasize that "doublets" identified in Xenium data are rarely actual doublets known in scRNA-seq data, where two or more cells are captured in the same bead/well. Xenium alternative of such doublets would be 2 cells segmented as one. Instead, Xenium "doublets" are mostly single-segmented cells that have infiltration of transcripts from neighboring cells that contaminate their profile. Therefore, we call such cells "contaminated" instead of "doublets". Nevertheless, we applied scDblFinder[1] – a doublet detection tool developed for scRNA-seq data – to compare its results to ones obtained with RCTD doublet calling. For the fair comparison with RCTD, we also provided cell-type labels derived from RCDT. We used the default parameters for other parameters.

Chromium (snRNA-seq) annotation and doublet detection with RCTD

To assess the high proportion of doublets detected by RCTD when applied to Xenium data, we simulated more homogeneous "Xenium-like" datasets from the matched Chromium data. This was done by restricting the Chromium data to subsets of genes

corresponding to different Xenium panels: Breast (n = 280), Lung (n = 289), Custom IO (n = 340), 5K (n ≈ 5,000), and genes shared between Lung and 5K panels (n = 194). RCTD was then applied to these simulated datasets using an external reference. We compared the resulting spot-class compositions (i.e., proportions of singlets and doublets) between the simulated Chromium data and the corresponding Xenium datasets from the same samples.

Data availability

Xenium

Publicly available Xenium data used for validation can be found here:
Breast : Breast https://www.10xgenomics.com/datasets/ffpe-human-breast-with-pre-designed-panel-1-standard.
PDAC : IO https://www.10xgenomics.com/datasets/ffpe-human-ductal-adenocarcinoma-data-with-human-immuno-oncology-profiling-panel-1-standard.
CRC : IO https://www.10xgenomics.com/datasets/ffpe-human-colorectal-cancer-data-with-human-immuno-oncology-profiling-panel-and-custom-add-on-1-standard.
MEL : 5K https://www.10xgenomics.com/datasets/xenium-prime-ffpe-human-skin.

sc/snRNA-seq

The matched breast cancer reference used for annotation of the public Breast:Breast sample can be found here: https://cf.10xgenomics.com/samples/cell-exp/7.0.1/Chromium_FFPE_Human_Breast_Cancer_Chromium_FFPE_Human_Breast_Cancer/Chromium_FFPE_Human_Breast_Cancer_Chromium_FFPE_Human_Breast_Cancer_count_sample_filtered_feature_bc_matrix.h5.
The external PDAC scRNA-seq reference used for annotation of the public PDAC : IO sample can be found here: https://zenodo.org/records/14199536.
The external CRC scRNA-seq reference used for annotation of the public CRC : IO sample can be found here: https://cf.10xgenomics.com/samples/cell-exp/8.0.0/HumanColonCancer_Flex_Multiplex/HumanColonCancer_Flex_Multiplex_count_filtered_feature_bc_matrix.h5.
The external MEL scRNA-seq reference used for annotation of the public MEL : 5K sample can be found here:
https://www.ncbi.nlm.nih.gov/geo/query/acc.cgi?acc=GSE115978.

References

40. Germain, P.-L., Lun, A., Meixide, C. G., Macnair, W. & Robinson, M. D. Doublet identification in single-cell sequencing data using *scDblFinder*. Preprint at https://doi.org/10.12688/f1000research.73600.2 (2022).

41. Huynh, K. L. A. *et al.* Deconvolution of cell types and states in spatial multiomics utilizing TACIT. *Nat. Commun.* **16**, 3747 (2025).

Supplementary Tables Legends/Captions

Supplementary Table 1. Overview of Xenium samples.

For each Xenium sample, the table includes panel information, the default segmentation used and whether matching snRNA-seq and IHC data are available.

Supplementary Table 2. Custom IO panel genes.

Supplementary Table 3. Annotated Lung panel genes.

Supplementary Table 4. Annotated Breast panel genes.

Supplementary Figures Legends/Captions

Supplementary Fig. 1. Consistency of Xenium data across patients and technical replicates in breast samples.

a, Integrated UMAPs of the breast samples profiled with the targeted Breast panel, faceted by sample. Cells are colored by RCTD-inferred cell type using matched Chromium data. **b,** Heatmap of cell-cell Euclidean distances for the breast Xenium samples, with annotations indicating cell type and sample of origin.

Supplementary Fig. 2. Consistency of Xenium data across patients and technical replicates in lung samples.

Integrated UMAPs of the lung samples profiled with the prime 5k (a), targeted Lung (b) and targeted Custom IO (c) panels, faceted by sample. Cells are colored by RCTD-assigned cell types using matched Chromium data. **d,** Heatmap of cell-cell Euclidean distances for the NSCLC Xenium samples, with annotations indicating cell type and sample of origin.

Supplementary Fig. 3. Cell class composition across segmentations.

a, RCTD spot-class composition for each Xenium sample, grouped by donor. **b,** RCTD-inferred cell class distributions across segmentation methods, all panels and samples. Box plots show the median and interquartile range (IQR), with whiskers extending to the nearest value outside 1.5× IQR. Breast: n=19 samples (17 biological and 3 technical replicates); Lung: n=11 samples (10 biological and 2 technical replicates); Custom IO: n=6 samples (5 biological and 2 technical replicates); 5K: n=6 biological replicates.

Supplementary Fig. 4. Comparison of cell-class composition obtained from scDblFinder and RCTD doublet detection.

a, Cell-class composition derived using RCTD (first bar) and scDblFinder (second bar). **b,** Confusion matrix between RCTD and scDblFinder cell-class assignments; values represent averages across samples. **c,** Spearman correlation between RCTD

and scDblFinder doublet scores, each dot corresponds to a sample. **d,** Distribution of median scDblFinder doublet scores per sample across RCTD-defined cell classes. **e,** Distribution of median RCTD doublet scores per sample across scDblFinder-defined cell classes. Box plots show the median and interquartile range (IQR), with whiskers extending to the nearest value outside 1.5× IQR. Breast: n=19 samples (17 biological and 3 technical replicates); Lung: n=11 samples (10 biological and 2 technical replicates); Custom IO: n=6 samples (5 biological and 2 technical replicates); 5K: n=6 biological replicates.

Supplementary Fig. 5. Comparison of cell-class composition obtained from TACIT and RCTD deconvolutions.

a, Cell-class composition derived using RCTD (first bar), TACIT in reference mode (second bar), and TACIT in signature mode (third bar). **b,** Confusion matrix between RCTD and reference-based TACIT cell-class assignments; values represent averages across samples. **c,** Spearman correlation between RCTD and TACIT doublet scores, each dot corresponds to a sample. **d,** Distribution of median TACIT doublet scores per sample across RCTD-defined cell classes. **e,** Distribution of median RCTD doublet scores per sample across TACIT-defined cell classes.

Supplementary Fig. 6. Comparison of cell-type annotations obtained from TACIT and RCTD deconvolutions.

a, Confusion matrices comparing cell-type annotations from RCTD (rows) and TACIT (columns) in both reference and signature modes. **b,** Confusion matrix comparing TACIT cell-type annotations between reference (rows) and signature (columns) modes.

Supplementary Fig. 7. Features of Xenium cells of different RCTD-derived spot classes.

a, Distribution of median cell area, number of detected genes and number of counts across RCTD-derived cell classes. **b,** Distribution of the secondary cell-type weight (w_2) in exposed cells (ie., cells with >10% of the secondary cell type present in their spatial neighborhood) among RCTD-assigned singlets, doublets, and rejects. Distributions are shown per sample across all panels. Color corresponds to RCTD decomposition type. Box plots show the median and interquartile range (IQR), with whiskers extending to the nearest value outside 1.5× IQR. Breast: n=19 samples (17 biological and 3 technical replicates); Lung: n=11 samples (10 biological and 2 technical replicates); Custom IO: n=6 samples (5 biological and 2 technical replicates); 5K: n=6 biological replicates.

Supplementary Fig. 8. Comparison of RCTD-derived cell-class composition in Chromium and Xenium data.

a, Scatterplot of Chromium cell doublet scores when transcriptomic profiles are restricted to gene sets corresponding to different Xenium panels. Trend line fitted with intercept fixed at zero is shown in blue, x=y line is shown in dashed black. b, RCTD spot-class composition for Chromium data restricted to the corresponding Xenium gene panels and for the matched Xenium samples.

Supplementary Fig. 9. Transcript spillover in public Xenium datasets and across segmentations.

a, Cosine similarity between the w_2 (weight of the secondary cell type) and the proportion of the secondary cell type in each cell's neighborhood in public Xenium datasets. **b**, RCTD-derived cell-class composition in the same public Xenium datasets. Each dataset is labeled as "Disease : 10x Panel." Abbreviations: PDAC, pancreatic ductal adenocarcinoma; CRC, colorectal cancer; MEL, melanoma; IO, Immuno-Oncology panel; 5K, 5K Prime panel. **c**, Cosine similarity between secondary cell-type weight (w_2) and the proportion of the secondary cell type in each cell's neighborhood in our data across different segmentation approaches. Box plots show the median and interquartile range (IQR), with whiskers extending to the nearest value outside 1.5× IQR. Breast: n=19 samples (17 biological and 3 technical replicates); Lung: n=11 samples (10 biological and 2 technical replicates); Custom IO: n=6 samples (5 biological and 2 technical replicates); 5K: n=6 biological replicates.

Supplementary Figures

Supplementary Figure 1

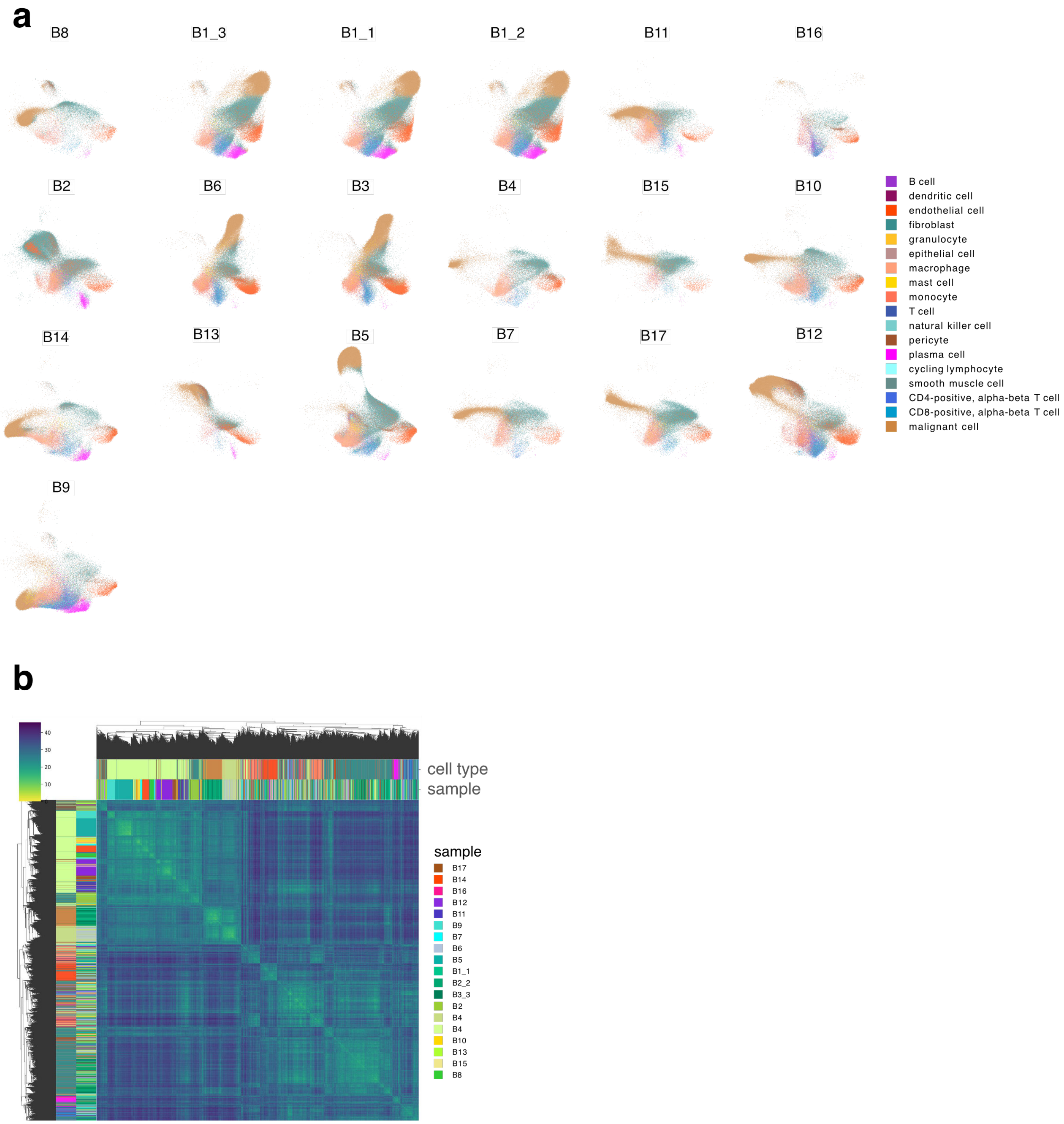

Supplementary Figure 2

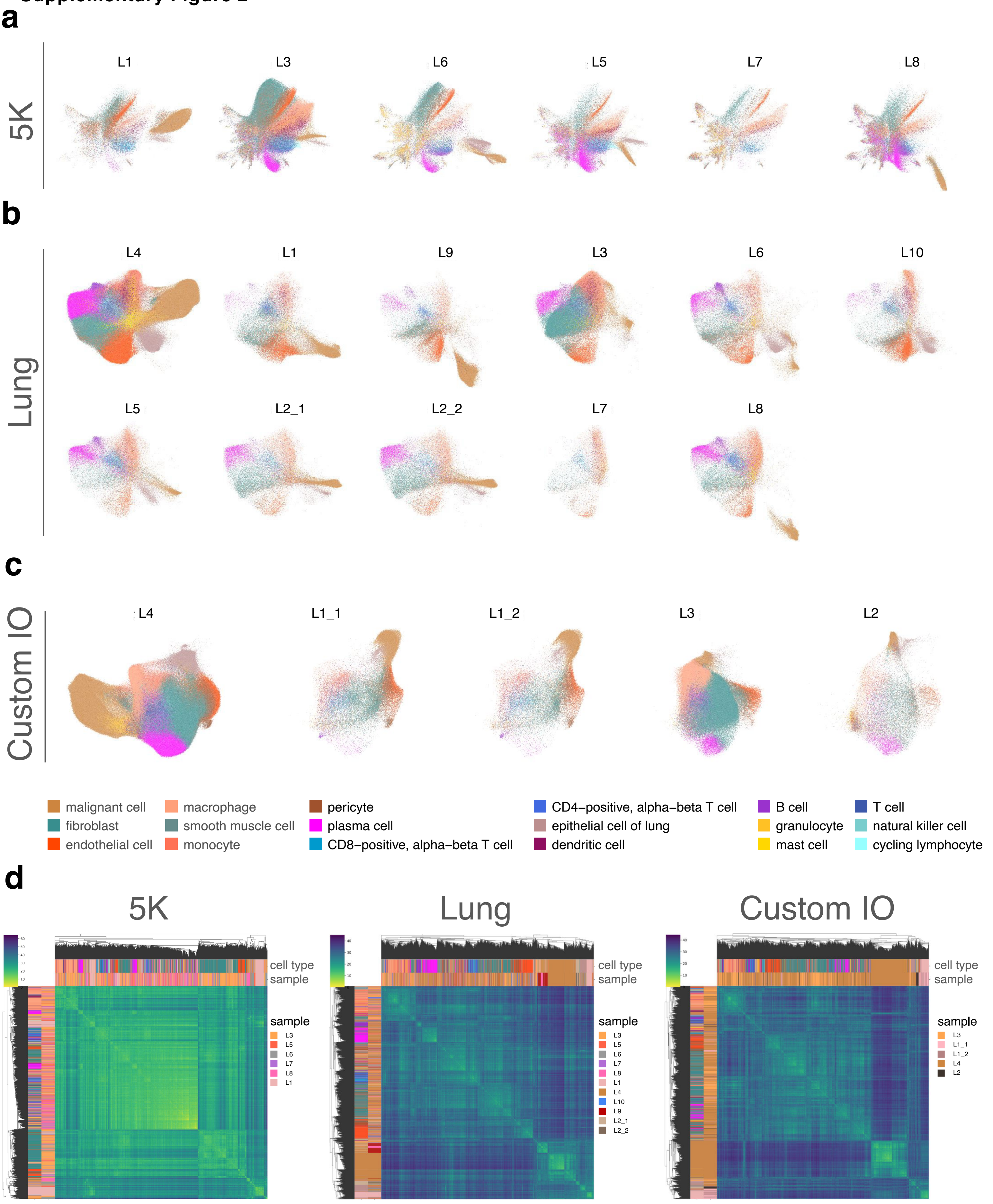

Supplementary Figure 3

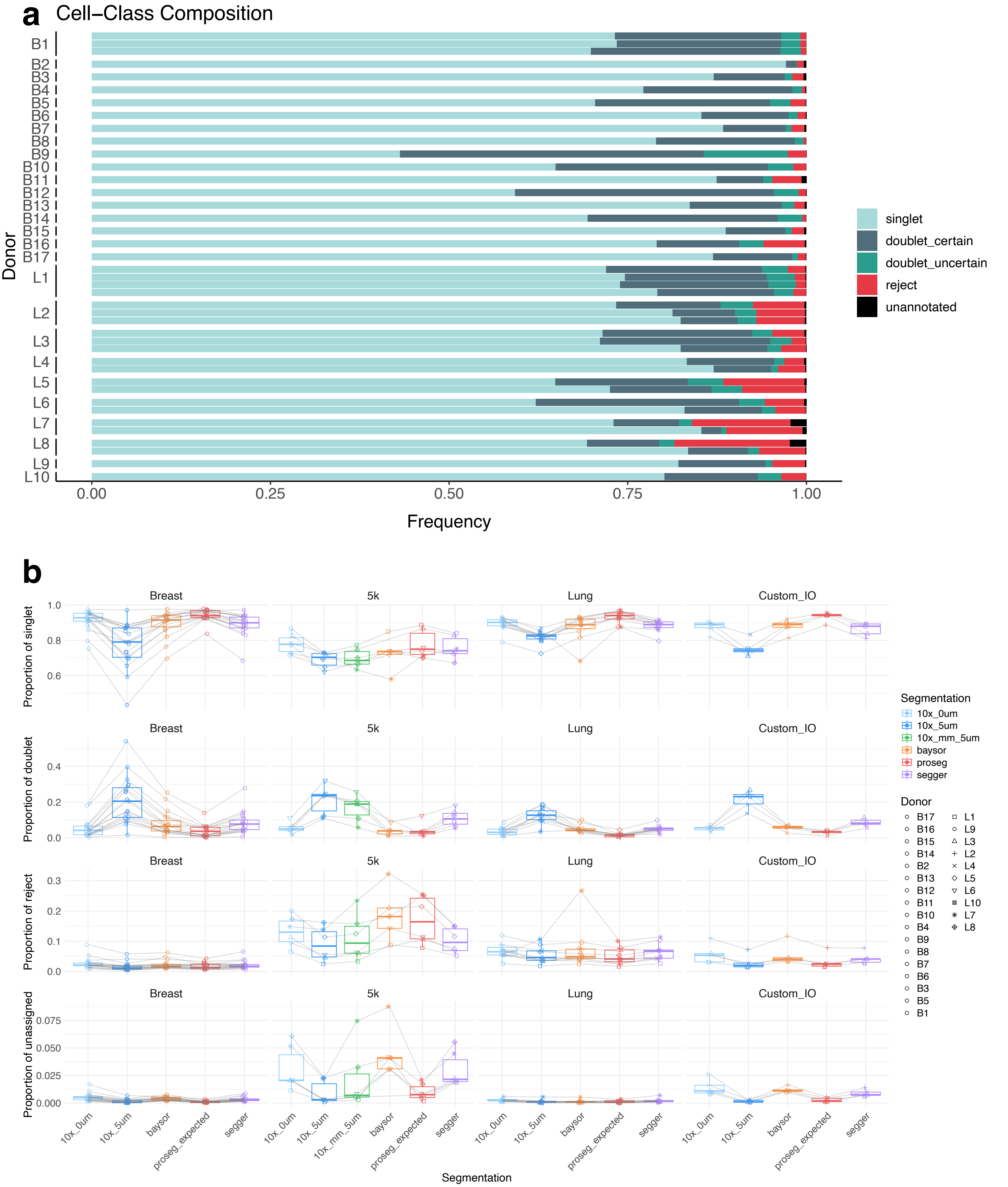

Supplementary Figure 4

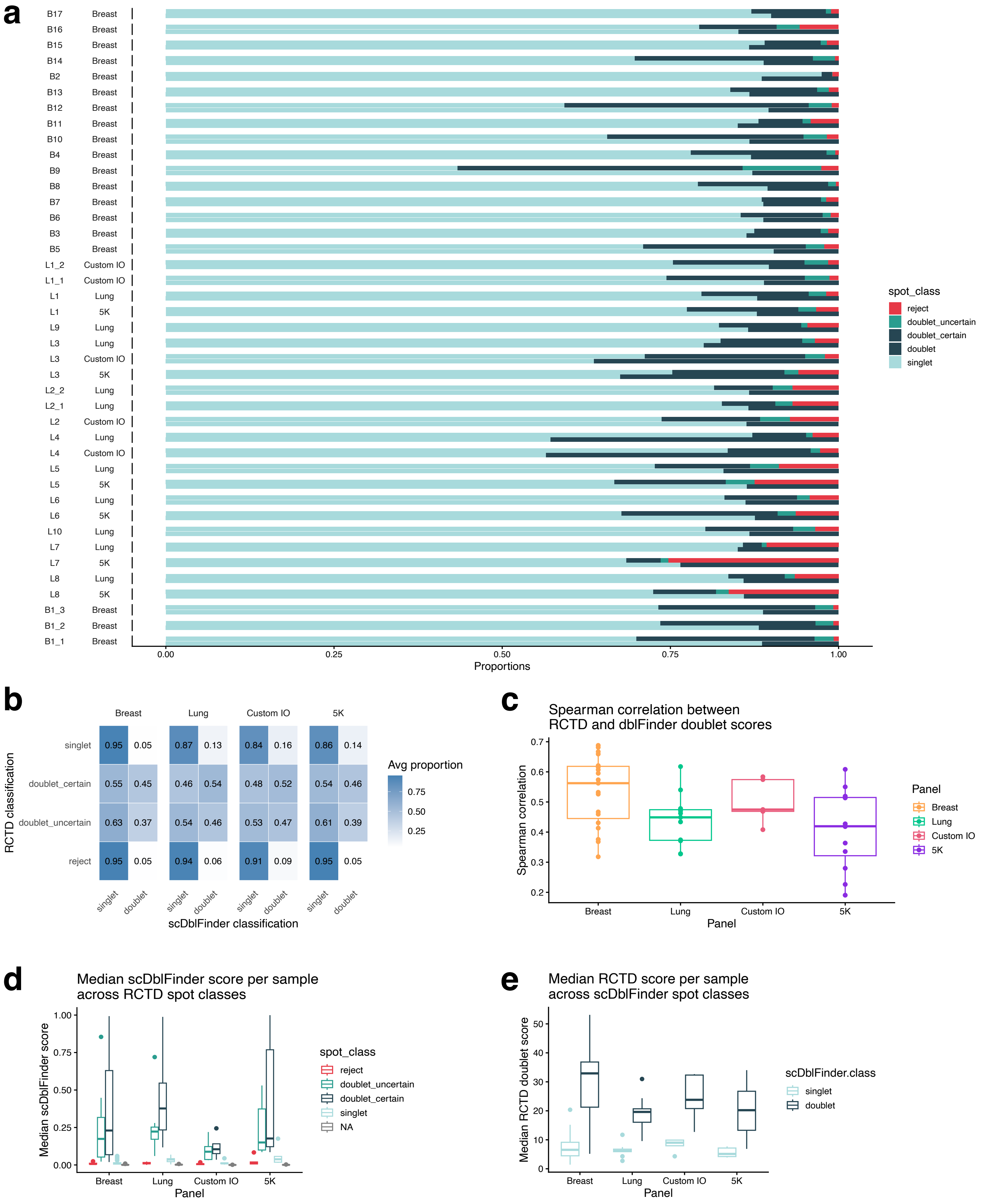

Supplementary Figure 5

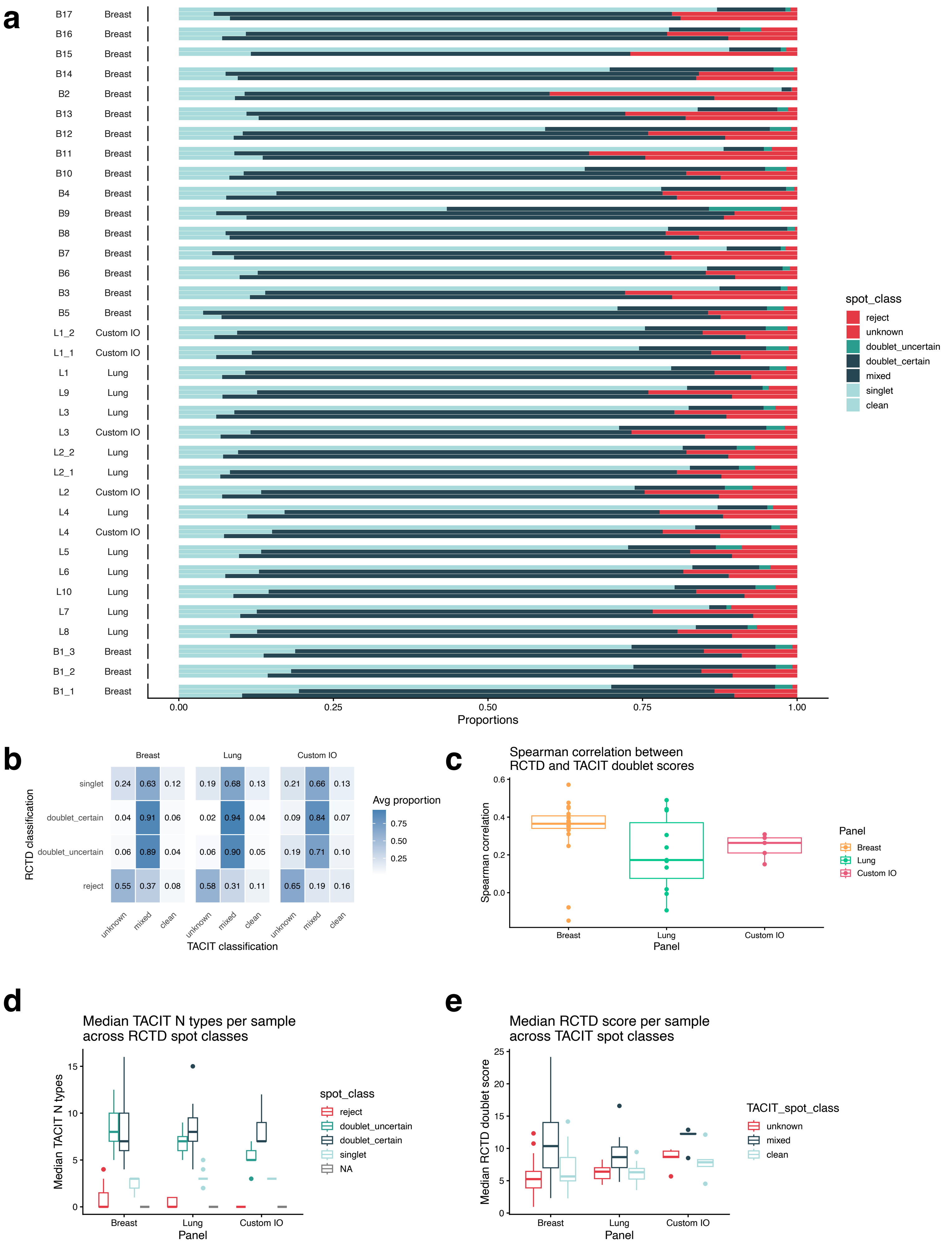

Supplementary Figure 6

a

Annotation agreement between RCTD and TACIT (reference)   Annotation agreement between RCTD and TACIT (signature)

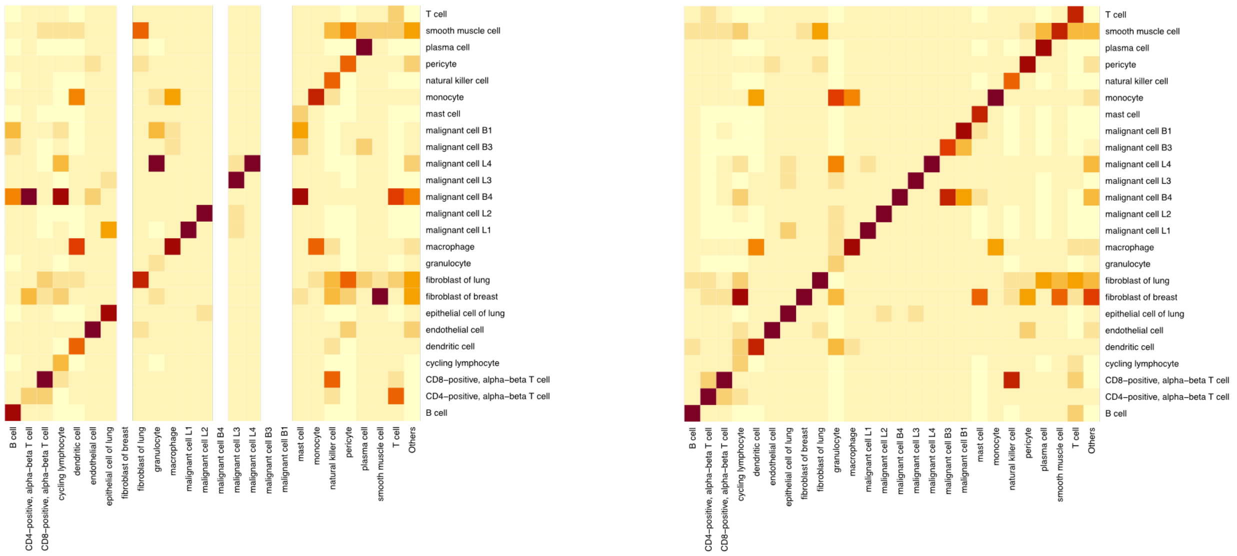

b

Annotation agreement between
TACIT (signature) and TACIT (reference)

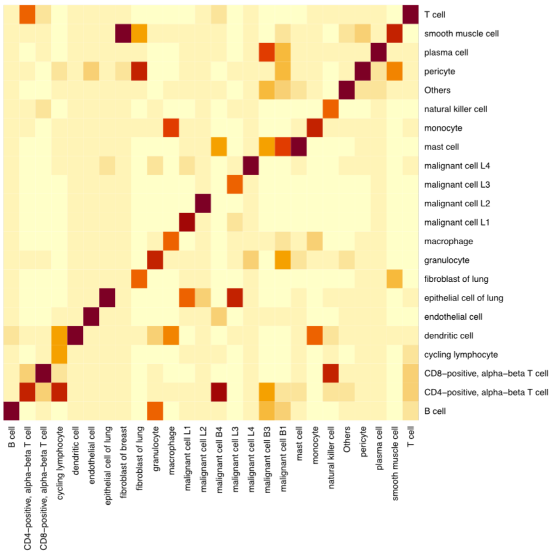

Supplementary Figure 7

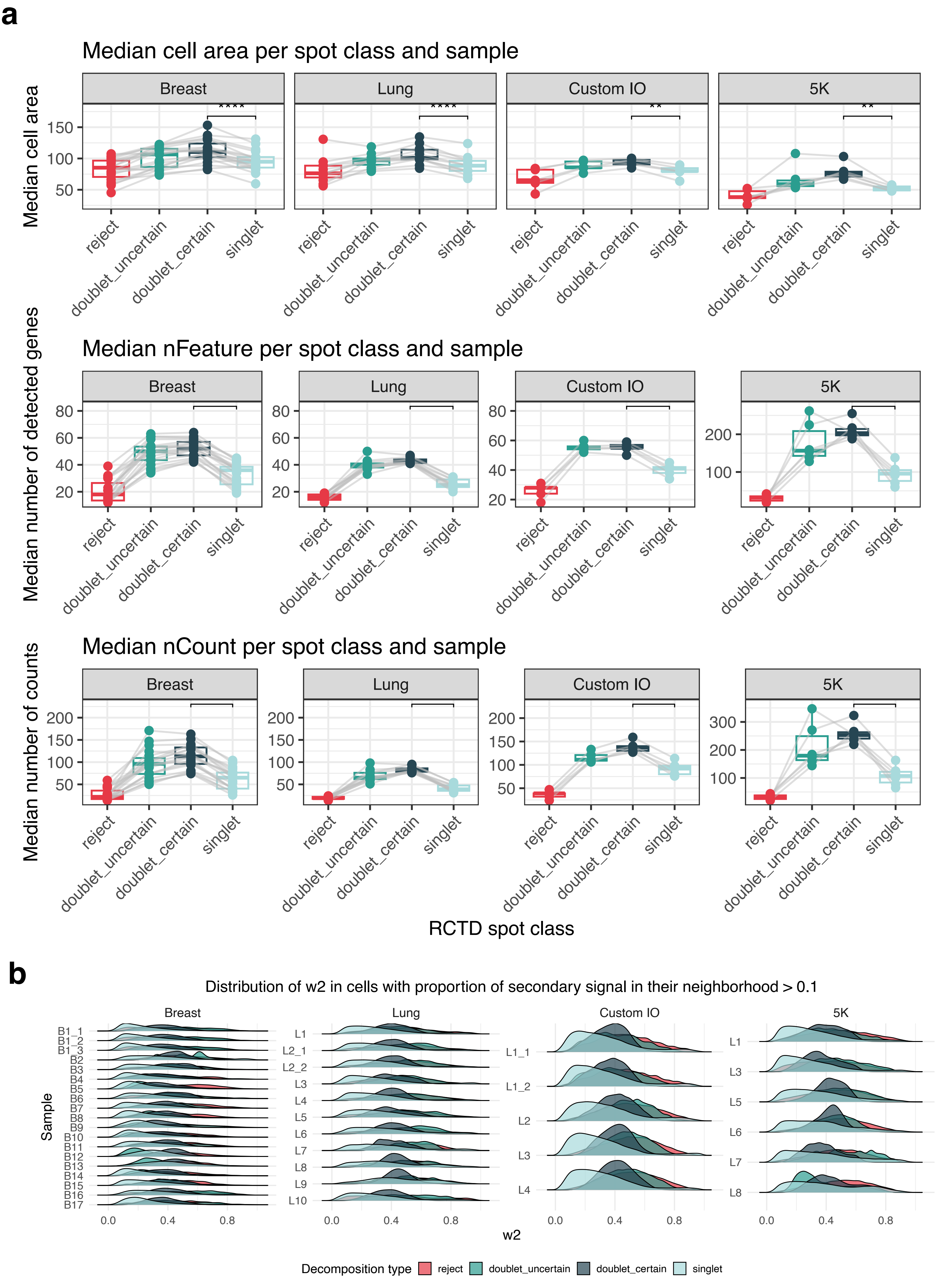

Supplementary Figure 8

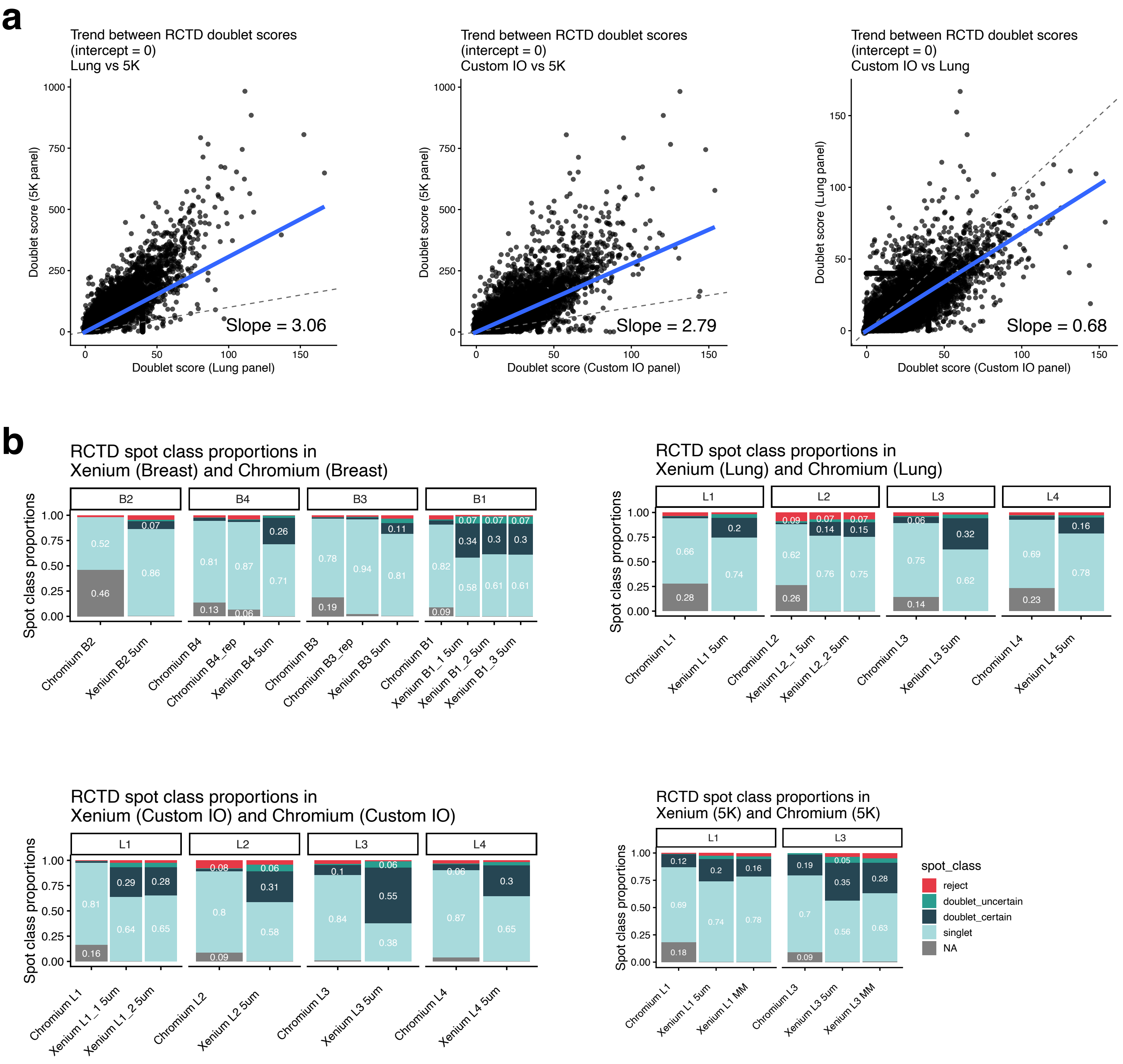

Supplementary Figure 9

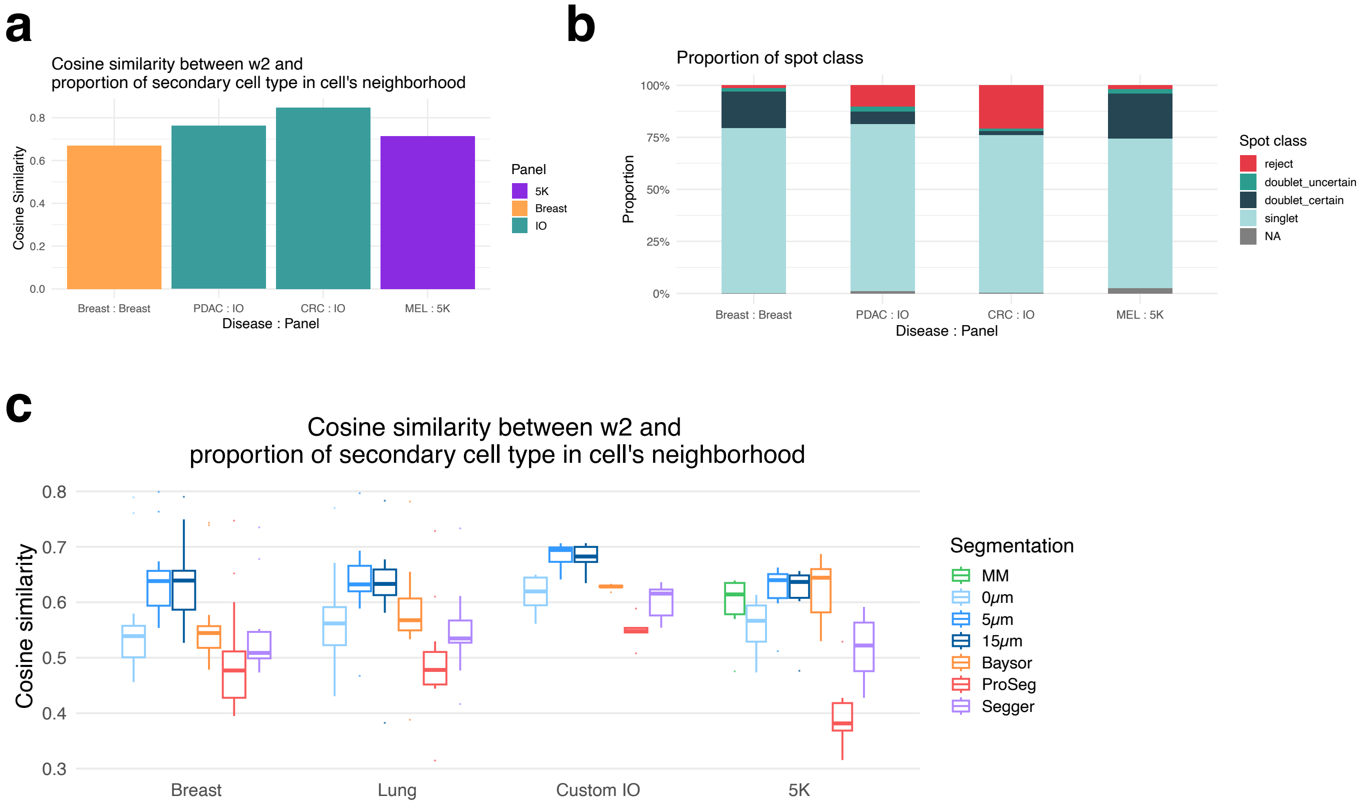

Supplement: Supplementary file 1 — Supplementary Information. [file 41592_2026_3089_MOESM1_ESM.pdf]
