# Supplementary material for: Resolving sensitivity, specificity and signal contamination in Xenium spatial transcriptomics
Source: Nat Methods. 2026 Apr 30;23(6):1152–62. doi: 10.1038/s41592-026-03089-8 (PMC13259927; doi:10.1038/s41592-026-03089-8)
Supplement: Supplementary file 2 — Reporting Summary [file 41592_2026_3089_MOESM2_ESM.pdf]

nature portfolio

Corresponding author(s): Raphael Gottardo, Krisztian Homicsko

Last updated by author(s): Jan 29, 2026

Reporting Summary

Nature Portfolio wishes to improve the reproducibility of the work that we publish. This form provides structure for consistency and transparency in reporting. For further information on Nature Portfolio policies, see our Editorial Policies and the Editorial Policy Checklist.

Statistics

For all statistical analyses, confirm that the following items are present in the figure legend, table legend, main text, or Methods section.

n/a	Confirmed	
☐	☒	The exact sample size (*n*) for each experimental group/condition, given as a discrete number and unit of measurement
☐	☒	A statement on whether measurements were taken from distinct samples or whether the same sample was measured repeatedly
☐	☒	The statistical test(s) used AND whether they are one- or two-sided<br>*Only common tests should be described solely by name; describe more complex techniques in the Methods section.*
☐	☒	A description of all covariates tested
☐	☒	A description of any assumptions or corrections, such as tests of normality and adjustment for multiple comparisons
☐	☒	A full description of the statistical parameters including central tendency (e.g. means) or other basic estimates (e.g. regression coefficient) AND variation (e.g. standard deviation) or associated estimates of uncertainty (e.g. confidence intervals)
☐	☒	For null hypothesis testing, the test statistic (e.g. *F*, *t*, *r*) with confidence intervals, effect sizes, degrees of freedom and *P* value noted<br>*Give P values as exact values whenever suitable.*
☒	☐	For Bayesian analysis, information on the choice of priors and Markov chain Monte Carlo settings
☒	☐	For hierarchical and complex designs, identification of the appropriate level for tests and full reporting of outcomes
☐	☒	Estimates of effect sizes (e.g. Cohen's *d*, Pearson's *r*), indicating how they were calculated

Our web collection on statistics for biologists contains articles on many of the points above.

Software and code

Policy information about availability of computer code

Data collection	Xenium Ranger v 3.0.1
Data analysis	Details on reproducing our analysis and analyzing other datasets can be found on GitHub (https://github.com/bdsc-tds/xenium_analysis_pipeline). SPLIT is available as an R package and can be found on GitHub (https://github.com/bdsc-tds/SPLIT). Snakemake workflow and scripts to produce figures: https://github.com/bdsc-tds/Bilous2026

For manuscripts utilizing custom algorithms or software that are central to the research but not yet described in published literature, software must be made available to editors and reviewers. We strongly encourage code deposition in a community repository (e.g. GitHub). See the Nature Portfolio guidelines for submitting code & software for further information.

Data

Policy information about availability of data

All manuscripts must include a data availability statement. This statement should provide the following information, where applicable:

- Accession codes, unique identifiers, or web links for publicly available datasets
- A description of any restrictions on data availability
- For clinical datasets or third party data, please ensure that the statement adheres to our policy

Xenium data produced as part of this study have been deposited in NCBI's Gene Expression Omnibus (GEO) and are accessible through GEO Series accession number GSE311609.

Raw data of our matched lung and breast cancer snRNA-seq references are publicly available from cellxgene at https://cellxgene.cziscience.com/collections/bd552f76-1f1b-43a3-b9ee-0aace57e90d6

The external lung scRNA-seq reference can be found here: https://cellxgene.cziscience.com/collections/edb893ee-4066-4128-9aec-5eb2b03f8287. We used the extended atlas, containing data from 318 patients, which accounts for over 1 million cells.

The external breast scRNA-seq reference, described by Wu et al, 2021, can be found here: https://www.ncbi.nlm.nih.gov/geo/query/acc.cgi?acc=GSE176078.

Research involving human participants, their data, or biological material

Policy information about studies with human participants or human data. See also policy information about sex, gender (identity/presentation), and sexual orientation and race, ethnicity and racism.

Reporting on sex and gender	NA (all data are fully identified)
Reporting on race, ethnicity, or other socially relevant groupings	NA (all data are fully identified)
Population characteristics	NA (all data are fully identified)
Recruitment	No recruitment. Samples come from an existing biobank
Ethics oversight	All patients provided informed consent for the use of the tumor samples in this study. The protocol used for sample collection was approved by the local ethics committee (CER-VD, BASEC ID 2016-02094).

Note that full information on the approval of the study protocol must also be provided in the manuscript.

Field-specific reporting

Please select the one below that is the best fit for your research. If you are not sure, read the appropriate sections before making your selection.

☒ Life sciences ☐ Behavioural & social sciences ☐ Ecological, evolutionary & environmental sciences

For a reference copy of the document with all sections, see nature.com/documents/nr-reporting-summary-flat.pdf

Life sciences study design

All studies must disclose on these points even when the disclosure is negative.

Sample size	41 tissue sections from breast and lung tumors across 27 donors
Data exclusions	No data exclusion
Replication	For Xenium, technical replicates were derived from adjacent tissue sections. The data demonstrate strong reproducibility among technical replicates while revealing substantial variability between patients and between technologies—namely, Chromium and Xenium. See Figure 1D and Extended Data Figures 3-4
Randomization	Not applicable, the study does not test multiple experimental groups
Blinding	Not applicable, the study does not test multiple experimental groups

Reporting for specific materials, systems and methods

We require information from authors about some types of materials, experimental systems and methods used in many studies. Here, indicate whether each material, system or method listed is relevant to your study. If you are not sure if a list item applies to your research, read the appropriate section before selecting a response.

Materials & experimental systems

n/a	Involved in the study
☐	☒ Antibodies
☒	☐ Eukaryotic cell lines
☒	☐ Palaeontology and archaeology
☐	☒ Animals and other organisms
☒	☐ Clinical data
☒	☐ Dual use research of concern
☒	☐ Plants

Methods

n/a	Involved in the study
☒	☐ ChIP-seq
☒	☐ Flow cytometry
☒	☐ MRI-based neuroimaging

Antibodies

Antibodies used

Two post-Xenium slides—one per disease—containing a total of five tissue sections were immunohistochemically stained for DAPI (Spectral DAPI, AKOYA), CD8 (Cellmarque, Clone SP16, 108R), and pan-cytokeratin (panCK, Dako, Clone AE1/AE3, IS053) in autostainer (ventana Discovery Ultra, Roche) using Tyramide Signal Amplification system (OPAL, Akoya), allowing us to identify $CD8^{+}$ T cells and malignant cells.

Validation

All primary antibodies were commercially sourced and validated by the manufacturers for immunohistochemistry on FFPE human tissue; expected staining patterns were confirmed by concordance with known tissue morphology and cell-type localization.

Animals and other research organisms

Policy information about studies involving animals; ARRIVE guidelines recommended for reporting animal research, and Sex and Gender in Research

Laboratory animals

Study did not involve laboratory animals.

Wild animals

Study did not involve wild animals.

Reporting on sex

Indicate if findings apply to only one sex; describe whether sex was considered in study design, methods used for assigning sex. Provide data disaggregated for sex where this information has been collected in the source data as appropriate; provide overall numbers in this Reporting Summary. Please state if this information has not been collected. Report sex-based analyses where performed, justify reasons for lack of sex-based analysis.

Field-collected samples

Study did not involve samples collected from the field.

Ethics oversight

Identify the organization(s) that approved or provided guidance on the study protocol, OR state that no ethical approval or guidance was required and explain why not.

Note that full information on the approval of the study protocol must also be provided in the manuscript.

Plants

Seed stocks

Study did not involve plants.

Novel plant genotypes

Describe the methods by which all novel plant genotypes were produced. This includes those generated by transgenic approaches, gene editing, chemical/radiation-based mutagenesis and hybridization. For transgenic lines, describe the transformation method, the number of independent lines analyzed and the generation upon which experiments were performed. For gene-edited lines, describe the editor used, the endogenous sequence targeted for editing, the targeting guide RNA sequence (if applicable) and how the editor was applied.

Authentication

Describe any authentication procedures for each seed stock used or novel genotype generated. Describe any experiments used to assess the effect of a mutation and, where applicable, how potential secondary effects (e.g. second site T-DNA insertions, mosiacism, off-target gene editing) were examined.
