# Supplementary material for: Resolving sensitivity, specificity and signal contamination in Xenium spatial transcriptomics
Source: Nat Methods. 2026 Apr 30;23(6):1152–62. doi: 10.1038/s41592-026-03089-8 (PMC13259927; doi:10.1038/s41592-026-03089-8)
Supplement: Supplementary file 3 — Peer Review File [file 41592_2026_3089_MOESM3_ESM.pdf]

nature portfolio

Peer Review File

From Transcripts to Cells: Dissecting Sensitivity, Signal Contamination, and Specificity in Xenium Spatial Transcriptomics

Corresponding Author: Professor Raphael Gottardo

This file contains all editorial decision letters in order by version, followed by all author rebuttals in order by version.

Version 0:

Decision Letter:

4th Sep 2025

Dear Dr Gottardo,

Your Article, "From Transcripts to Cells: Dissecting Sensitivity, Signal Contamination, and Specificity in Xenium Spatial Transcriptomics", has now been seen by four reviewers, including one co-reviewer. As you will see from their comments below, although the reviewers find your work of considerable potential interest, they have raised a number of concerns regarding its conceptual novelty and the technical aspects of the analyses. We continue to be interested in potentially publishing your manuscript in Nature Methods, but would like to evaluate your response to these concerns before we reach a final decision on publication.

We therefore invite you to revise your manuscript to address these concerns. We are committed to providing a fair and constructive peer-review process. Do not hesitate to contact us if there are specific requests from the reviewers that you believe are technically impossible or unlikely to yield a meaningful outcome.

When revising your paper:

* include a point-by-point response to the reviewers and to any editorial suggestions

* please underline/highlight any additions to the text or areas with other significant changes to facilitate review of the revised manuscript

* address the points listed described below to conform to our open science requirements

* ensure it complies with our general format requirements as set out in our guide to authors at www.nature.com/naturemethods

* resubmit all the necessary files by using the link below to access your home page

Link Redacted

Note: This URL links to your confidential account and associated information about manuscripts you may have submitted, or that you are reviewing for us. If you wish to forward this email to co-authors, please delete this link.

We hope to receive your revised paper within 12 weeks. If you are substantially delayed, please let us know. In this event, we will still be happy to reconsider your paper at a later date so long as nothing similar has been accepted for publication at Nature Methods or published elsewhere.

OPEN SCIENCE REQUIREMENTS

REPORTING SUMMARY
When revising your manuscript, please update your reporting summary.

Reporting summary: https://www.nature.com/documents/nr-reporting-summary.zip

If your paper includes custom software, we also ask you to complete a supplemental reporting summary.

Software supplement: https://www.nature.com/documents/nr-software-policy.pdf

Please submit these with your revised manuscript. They will be available to reviewers to aid in their evaluation if the paper is re-reviewed. If you have any questions about the checklist, please see http://www.nature.com/authors/policies/availability.html or contact me.

Please note that these forms are dynamic 'smart pdfs' and must therefore be downloaded and completed in Adobe Reader. We will then flatten them for ease of use by the reviewers. If you would like to reference the guidance text as you complete the template, please access these flattened versions at http://www.nature.com/authors/policies/availability.html.

For any revision that includes light microscopy data, we ask our authors to please include a completed light microscopy reporting table [https://www.nature.com/documents/Light_microscopy_reporting_table.xlsx] to ensure the methods are described thoroughly. The table will be available to reviewers and ultimately published should the manuscript be accepted at the journal.

IMAGE INTEGRITY
When submitting the revised version of your manuscript, please pay close attention to our Digital Image Integrity Guidelines and to the following points below:

-- that unprocessed scans are clearly labelled and match the gels and western blots presented in figures.
-- that control panels for gels and western blots are appropriately described as loading on sample processing controls
-- all images in the paper are checked for duplication of panels and for splicing of gel lanes.

Finally, please ensure that you retain unprocessed data and metadata files after publication, ideally archiving data in perpetuity, as these may be requested during the peer review and production process or after publication if any issues arise.

EXTENDED DATA FIGURES
When re-submitting your manuscript, please ensure that any supplementary figures and tables that are crucial to the manuscript's conclusions are converted into Extended Data figures and tables to increase visibility of these data. Extended Data figures and tables are online-only (present in the online PDF and full-text HTML versions of the paper), peer-reviewed display items that provide essential background to the article but are not included in the main article due to space constraints. A maximum of ten Extended Data display items (figures and tables) is permitted.

DATA AVAILABILITY
We strongly encourage you to deposit all new data associated with the paper in a persistent repository where they can be freely and enduringly accessed. We recommend submitting the data to discipline-specific and community-recognized repositories; a list of repositories is provided here: http://www.nature.com/sdata/policies/repositories

All novel DNA and RNA sequencing data, protein sequences, genetic polymorphisms, linked genotype and phenotype data, gene expression data, macromolecular structures, and proteomics data must be deposited in a publicly accessible database, and accession codes and associated hyperlinks must be provided in the "Data Availability" section.

For papers containing bioimaging data, we strongly recommend depositing the data to Bioimage Archive (https://www.ebi.ac.uk/bioimage-archive/). Associated accession codes and hyperlinks should be provided in the "Data Availability" section.

Refer to our data policies here: https://www.nature.com/nature-research/editorial-policies/reporting-standards#availability-of-data

To further increase transparency, we encourage you to provide, in tabular form, the data underlying the graphical representations used in your figures. This is in addition to our data-deposition policy for specific types of experiments and large datasets. For readers, the source data will be made accessible directly from the figure legend. Spreadsheets can be submitted in .xls, .xlsx or .csv formats. Only one (1) file per figure is permitted: thus if there is a multi-paneled figure the source data for each panel should be clearly labeled in the csv/Excel file; alternately the data for a figure can be included in multiple, clearly labeled sheets in an Excel file. File sizes of up to 30 MB are permitted. When submitting source data files with your manuscript please select the Source Data file type and use the Title field in the File Description tab to indicate which figure the source data pertains to.

Please include a “Data availability” subsection in the Online Methods. This section should inform readers about the availability of the data used to support the conclusions of your study, including accession codes to public repositories, references to source data that may be published alongside the paper, unique identifiers such as URLs to data repository entries, or data set DOIs, and any other statement about data availability. At a minimum, you should include the following statement: “The data that support the findings of this study are available from the corresponding author upon request”, describing which data is available upon request and mentioning any restrictions on availability. If DOIs are provided, please include these in the Reference list (authors, title, publisher (repository name), identifier, year). For more guidance on how to write this section please see: http://www.nature.com/authors/policies/data/data-availability-statements-data-citations.pdf

CODE AVAILABILITY
Please include a “Code Availability” subsection in the Online Methods which details how your custom code is made available. Only in rare cases (where code is not central to the main conclusions of the paper) is the statement “available upon request” allowed (and reasons should be specified).

We request that you deposit code in a DOI-minting repository such as Zenodo, Gigantum or Code Ocean and cite the DOI in the Reference list. We also request that you use code versioning and provide a license.

For more information on our code sharing policy and requirements, please see: https://www.nature.com/nature-research/editorial-policies/reporting-standards#availability-of-computer-code

MATERIALS AVAILABILITY
As a condition of publication in Nature Methods, authors are required to make unique materials promptly available to others without undue qualifications.

Authors reporting new chemical compounds must provide chemical structure, synthesis and characterization details. Authors reporting mutant strains and cell lines are strongly encouraged to use established public repositories.

More details about our materials availability policy can be found at https://www.nature.com/nature-portfolio/editorial-policies/reporting-standards#availability-of-materials

SUPPLEMENTARY PROTOCOL
To help facilitate reproducibility and uptake of your method, we ask you to prepare a step-by-step Supplementary Protocol for the method described in this paper. We encourage authors to share their step-by-step experimental protocols on a protocol sharing platform of their choice and report the protocol DOI in the reference list. Nature Portfolio's protocols.io is a free-to-use and open resource for protocols; protocols deposited onto protocols.io are citable and can be linked from the published article. More details can found at protocols.io.

ORCID
Nature Methods is committed to improving transparency in authorship. As part of our efforts in this direction, we are now requesting that all authors identified as ‘corresponding author’ on published papers create and link their Open Researcher and Contributor Identifier (ORCID) with their account on the Manuscript Tracking System (MTS), prior to acceptance. This applies to primary research papers only. ORCID helps the scientific community achieve unambiguous attribution of all scholarly contributions. You can create and link your ORCID from the home page of the MTS by clicking on ‘Modify my Springer Nature account’. For more information please visit please visit www.springernature.com/orcid.

Please do not hesitate to contact me if you have any questions or would like to discuss these revisions further. We look forward to seeing your revised manuscript and thank you for the opportunity to consider your work.

Best regards,
Lei

Lei Tang, Ph.D.
Senior Editor
Nature Methods

Reviewers' Comments:

Reviewer #1 (Remarks to the Author):

This manuscript investigated the quality and errors in Xenium data, including the latest 5K gene panel, using a large dataset consisting of over 40 slices of breast and lung tumor samples. This analysis revealed the prevalent problem of transcript diffusion in Xenium data and presented a method, SPLIT, to correct for the blurred gene expression signal due to transcript diffusion. This work has a high impact on the emerging Xenium data analysis. However, I have the following concerns.

Major comments:
1. The main analysis is based on the labeling of doublets for each Xenium spot. In order for the conclusion to hold, the labeling of doublets should be very accurate. Despite that RCTD is generally an accurate deconvolution method, only using RCTD does not establish the reliability of doublet detection.
(1). Are any of the scRNA-seq doublet detection method applicable to Xenium data if ignoring spatial coordinates? Do they identify consistent proportion of doublets or trends of doublet proportions as RCTD?
(2). A recent method TACIT (https://www.nature.com/articles/s41467-025-58874-4) also mentioned identifying mixed spots and performing deconvolution for xenium data. How does the mixed spots identified by TACIT compared to RCTD?
(3). It may be possible to evaluate the potential errors when applying RCTD to deconvolve near-single-cell gene expression of a small gene panels by realistic simulated data. Particularly, the matched scRNA-seq suffer less from "transcript diffusion" problem, and therefore it's possible to simulate Xenium data by selecting genes and sub-sampling UMI counts from scRNA-seq data. Applying RCTD on the simulated data using scRNA-seq, and using another scRNA-seq as reference to avoid double dipping, the results are expected to be all singlets if the method doesn't have any errors. If the results are not all singlets, it will inform the potential error rates from the computational method.
2. Quantifying horizontal transcript diffusion is a fascinating idea. However, I have a few questions on the details of how transcript diffusion is quantified and analyzed.
(1). It's unclear to me what are possible explanations of "doublets" in xenium data, is it only horizontal diffusion and vertical diffusion? Particularly, the 5K panel has a lower degree of horizontal diffusion but higher rate of doublets, then what explains the high rate of doublets in 5K data?
(2). The definition of diffusion index is hard to parse.
(3). It seems that the diffusion index computes the correlation between each pair of primary and secondary cell type. I didn't get why it is important to consider the primary cell type to quantify the tendency of secondary cell type to diffuse, instead of ignoring the primary cell type and compute a single correlation for each secondary cell type.
(4). How many spots/numbers are used to compute each correlation value? If a secondary cell type is rare (say, only a few spots have cell type A as secondary cell type), then the correlation value may not be reliable.
(5). Is diffusion index plot in Fig 3d computed using one lung sample or multiple lung samples? If computing on each individual lung sample, are the diffusion index consistent across slices for each patient or gene panels?
(6). On line 421, a logistic regression model to predict to predict secondary cell type based on neighboring cell type is mentioned, how does the logistic regression model compare to the diffusion index defined based on correlations?
3. The doublet analysis is performed on the breast and lung sections generated by this study. Are the doublet proportions generalizable to other published Xenium slices if applying the same method on them?
4. The separability of cell types in UMAP space across the technologies is only visually compared (Fig 2a, S5h, line 280). However, the visual comparison may be affected by many plotting artifacts, e.g., the number of points in the plot and whether or not using transparency. I suggest using quantitative metrics to indicate the separability of clusters, such as silhouette score, Calinski and Harabasz score, and Davies-Bouldin score.
5. SPLIT is based on RCTD, which requires a reference scRNA-seq data. If scRNA-seq data of the same patient is not available, what's the accuracy of SPLIT using a different scRNA-seq reference?
6. On line 329, "residual expression of transcripts fr om neighboring cell types is detected", for readers who are not experts in reading IHC images, is there an explanation on how to read the residual expression from the image?
7. In SPLIT method description, line 707 introduces the formula of x_{doublet-SPLIT}. Are ref_1 and ref_2 vectors? Are the computation elemenwise? This should be clarified.

Minor:
1. "This is evident in Figure 3h, where singlet-classified cells still show signs of contamination", is it referring to a different figure panel as 3h is the illustration of SPLIT. Please also check the reference to other figure panels.
2. In figure 3g, does the y axis label "proportion of the tumor signal in cell's neighborhood" mean the proportion of spots whose primary cell type is tumor? Or does it mean other quantities?

Reviewer #1 (Remarks on code availability):

The code installation is smooth. But I recommend providing the memory and runtime information for the tutorial examples, which will be very useful for HPC computing job scheduling.

Reviewer #2 (Remarks to the Author):

This manuscript presents a comprehensive analysis of signal quality in Xenium, with a particular focus on comparing whole transcriptome (5K) versus targeted panels. A key contribution is the introduction of the SPLIT method, designed to identify and address cell missegmentation, which is a known limitation in imaging-based spatial transcriptomics. The study highlights how misassigned transcripts can impact downstream analysis and proposes both empirical assessments and computational strategies to address this issue.
The study tackles an important problem in spatial transcriptomics and provides a valuable dataset with well-executed profiling of two tumor types. The comparison between panels and segmentation strategies is timely and informative. However, the manuscript suffers from some conceptual and presentation issues that limit its impact. In particular, the uncritical use of the term

"diffusion" is problematic, and several analytical claims require more nuanced interpretation. With revisions, the manuscript could become a strong contribution to the field.
Major Comments
The most significant issue concerns the terminology and interpretation surrounding the concept of "diffusion." Throughout the manuscript, the authors use the term indistinctly to describe transcript spillover between cells. However, diffusion implies a specific biophysical process involving random molecular movement through a medium (e.g., 2D or 3D space). The manuscript does not provide evidence that such a process is occurring. In fact, reported findings—such as cell type–specific "diffusion"—suggest the phenomenon is more likely due to technical artifacts such as missegmentation or incorrect assignment of transcripts.
I strongly recommend the authors reconsider the terminology used. More accurate terms would be *spillover*, *misassignment*, or *signal contamination*. Persisting with "diffusion" without clear justification introduces conceptual confusion and detracts from the rigor of the study.
There are also some issues with respect to novelty for the first part of the paper (figs 1-3), where Xenium data are compared to single cell/nucleus data and other imaging based spatially resolved methods, and where the extent of contamination is evaluated. For the quantitative and qualitative comparisons of data set, there are now a number of papers presenting this, some cited by the authors, and for the contamination from neighboring cells, this has been shown and elaborated in the recent publication by Marco Salas, S. et al, Nat Methods 22, 813-823 (2025), a study not cited by the authors.

Minor Comments

-Use of RCTD and cell decomposition: While valid, this is a highly specific approach. A short explanation of its niche applicability and limitations would improve accessibility for a broader audience.
-Figure 2b: The grey and blue colors are difficult to distinguish. Consider adjusting the color palette to improve visual clarity.
-Line 54: The description of Xenium as an “in situ hybridization technique” is inaccurate. Xenium relies on rolling circle amplification and padlock probes. “Imaging-based spatial transcriptomics” would be a more appropriate term.
-Line 76-77: The authors mention that prior studies are limited in scale and patient diversity. However, this study is itself limited in tissue types, focusing on only two tumor types. This limitation should be acknowledged.
-Based on our RCTD analyses..." (Methods section): This interpretation of mixed signals as “doublets” has already been described in Marco Salas et al. Please cite accordingly and clarify the novelty, if any.
-Figure 3c and correlation metrics: The observed lower correlation in the 5K panel is interpreted as reduced diffusion. This is a speculative conclusion. Other factors could explain the result, including differences in segmentation accuracy or RCTD performance. Additionally, correlation is sensitive to outliers; consider using cosine similarity or other robust metrics for this comparison.
-"Diffusion index" (Figure 3d): The notion of a “cell type–specific diffusion index” is contradictory if the process were truly physical diffusion—it should not vary by cell type. This again underscores the need to revise the terminology.
-UMAP visualization: The manuscript refers extensively to UMAPs for interpretation. While useful for exploratory analysis, UMAP embeddings are sensitive to graph construction parameters, sample size, and preprocessing. Some caution in interpreting visual differences as biological should be added.
-Figure 4 and segmentation comparison: This is a valuable part of the manuscript but currently hard to interpret. The connection between SCIB integration metrics and segmentation accuracy needs stronger justification. Panels 4c and 4d seem tangential and could be moved to supplementary material.
-Line 515: The manuscript mentions niche analysis being impacted by signal contamination, but this is never demonstrated. Please provide supporting analysis or remove the statement.
-SPLIT limitations: The discussion should acknowledge that SPLIT requires a reference single-cell or single-nucleus dataset for decomposition, which may not always be available in all experimental contexts.
-It’s not clear what Chromium chemistry was used to generate the snRNAseq data.

Reviewer #3 (Remarks to the Author):

I co-reviewed this manuscript with one of the reviewers who provided the listed reports. This is part of the Nature Methods initiative to facilitate training in peer review and to provide appropriate recognition for Early Career Researchers who co-review manuscripts.

Reviewer #4 (Remarks to the Author):

Segmented cell data from Xenium are prone to mixed signals due to the presence of doublets and transcript diffusion across neighboring cells. To address this, the authors introduce SPLIT, a computational pipeline designed to decompose transcripts within segmented cells. The method applies RCTD, originally developed for Visium spot-level deconvolution—to partition the transcripts of each cell according to its inferred mixture of cell types.

While the study tackles an important methodological challenge, the analyses presented are largely descriptive and lack rigorous quantitative validation to substantiate several of the key claims. For example, the authors conclude from Figure 1e that “cells of the same type demonstrated strong concordance across samples without the need for batch correction.” However, it is not evident what this conclusion is based on—whether it arises solely from qualitative inspection of the UMAP, or whether any formal metric of concordance was applied.

An important issue concerns the integration of spatial single-cell data across tissue samples. The manuscript does not provide sufficient methodological detail on how batch effects or sample-specific effects are addressed, if at all, during integration. Indeed, Figure 1d suggests that factors beyond cell type may be driving the observed separation among cells, raising questions about the robustness of the approach.

Similarly, in Line 389, the authors state that "SPLIT-corrected cells reveal more distinct clustering and improved separation compared to the original mixed signals." Once again, this conclusion is presented in descriptive terms without supporting quantitative benchmarks. Formal statistical assessments would be essential to support this claim.

Version 1:

Decision Letter:

Our ref: NMETH-A61440A

17th Dec 2025

Dear Dr. Gottardo,

Thank you for submitting your revised manuscript "From Transcripts to Cells: Dissecting Sensitivity, Signal Contamination, and Specificity in Xenium Spatial Transcriptomics" (NMETH-A61440A). It has now been seen by the original referees and their comments are below. The reviewers find that the paper has improved in revision, and therefore we'll be happy in principle to publish it in Nature Methods, pending minor revisions to satisfy the referees' final requests and to comply with our editorial and formatting guidelines.

We are now performing detailed checks on your paper and will send you a checklist detailing our editorial and formatting requirements within two weeks or so (realistically only in Jan). Please do not upload the final materials and make any revisions until you receive this additional information from us.

TRANSPARENT PEER REVIEW
Nature Methods offers a transparent peer review option for new original research manuscripts submitted from 17th February 2021. We encourage increased transparency in peer review by publishing the reviewer comments, author rebuttal letters and editorial decision letters if the authors agree. Such peer review material is made available as a supplementary peer review file. **Please state in the cover letter 'I wish to participate in transparent peer review' if you want to opt in, or 'I do not wish to participate in transparent peer review' if you don't.** Failure to state your preference will result in delays in accepting your manuscript for publication.

Please note: we allow redactions to authors' rebuttal and reviewer comments in the interest of confidentiality. If you are concerned about the release of confidential data, please let us know specifically what information you would like to have removed. Please note that we cannot incorporate redactions for any other reasons. Reviewer names will be published in the peer review files if the reviewer signed the comments to authors, or if reviewers explicitly agree to release their name. For more information, please refer to our FAQ page.

ORCID
IMPORTANT: Non-corresponding authors do not have to link their ORCIDs but are encouraged to do so. Please note that it will not be possible to add/modify ORCIDs at proof. Thus, please let your co-authors know that if they wish to have their ORCID added to the paper they must follow the procedure described in the following link prior to acceptance:
https://www.springernature.com/gp/researchers/orcid/orcid-for-nature-research

Author names using non-Roman characters
Nature Portfolio journals can support presentation of author names using non-Roman characters in the HTML version of the article. If you wish to, please include author names in parentheses after the Roman-character spelling; see example online here. Currently supported scripts are: Arabic, Chinese, Cyrillic, Devanagari, Greek, Hebrew, Hangul, Japanese and Persian. You will be asked to verify the rendering is correct at proof stage.

Thank you again for your interest in Nature Methods. Please do not hesitate to contact me if you have any questions. We will be in touch again soon.

Happy Holidays!

Sincerely,
Madhura

Madhura Mukhopadhyay, PhD
Senior Editor
Nature Methods

Reviewer #1 (Remarks to the Author):

The revised manuscript is so much clearer and addressed my previous concerns. I have the following clarification questions that will further help me better understand the details.

1. Main text line 332, “the local relative abundance of that same cell type in the surrounding 2D neighborhood”, does the quantity refer to the proportion of neighboring cells with primary cell type being the same cell type, or the sum of RCTD weights of this cell type regardless of primary or secondary cell type?
2. In Figure 1b, the sample/donor in cell count barplot panel is hard to tell partially because donors have different number of samples under different technologies. It would be easier to parse if donor/sample annotation is better denoted.
3. In Figure 1c, there is no ambiguous/unannotated cells in Xenium samples, but Figure S1a contains “reject” and “unannotated” cells. Is Fig 1c plotted by filtering out unannotated cells or are the unannotated cells further manually annotated by some procedure?

Reviewer #1 (Remarks on code availability):

The updated code is satisfactory to me.

Reviewer #2 (Remarks to the Author):

The revised version has improved a lot in terms of clarity. I still find the "singlet"/"doublet" terminology confusing and would recommend "pure"/"mixed" instead for clarity.
otherwise I think the revised manuscript is acceptable for publication. It is an interesting and important contribution.

Reviewer #2 (Remarks on code availability):

The code worked and instructions were sufficient. It runs a bit slow, and the authors could include some text in the manuscript about run-time and memory requirements.

Reviewer #3 (Remarks to the Author):

I co-reviewed this manuscript with one of the reviewers who provided the listed reports. This is part of the Nature Methods initiative to facilitate training in peer review and to provide appropriate recognition for Early Career Researchers who co-review manuscripts.

Reviewer #4 (Remarks to the Author):

In response to my earlier comments, the authors have added calculations of scores for biological conservation and batch integration. However, several concerns remain.

Supplementary Figure 4 presents seven different biological conservation metrics across platforms and experiments. The reported values span a wide range—from less than 1 to over 1000—yet the manuscript provides no guidance on how these scores should be interpreted or what the color scales represent. Similarly, the authors state that “batch integration metrics further confirm strong concordance of cell types across samples,” but it is unclear how this conclusion is supported by the table of numerical values, especially given the inconsistent color coding and lack of explanation. The same issue applies to the batch integration metrics, where results across methods are not always concordant and their implications are not clearly articulated.

More broadly, the paper suffers from an overabundance of analyses and metrics. Numerous claims are made hastily without adequate explanation or justification. Several figures are overcrowded with panels, many of which are difficult to read or interpret. The manuscript would benefit greatly from a substantial rewrite and tightening of the narrative, ideally with active involvement from the senior authors to ensure clarity and coherence.

Version 2:

Decision Letter:

27th Mar 2026

Dear Raphael,

I am pleased to inform you that your Article, "From Transcripts to Cells: Dissecting Sensitivity, Signal Contamination, and Specificity in Xenium Spatial Transcriptomics", has now been accepted for publication in Nature Methods. The received and

accepted dates will be Jun 24, 2025 and Mar 27, 2026. This note is intended to let you know what to expect from us over the next month or so, and to let you know where to address any further questions.

Over the next few weeks, your paper will be copyedited to ensure that it conforms to Nature Methods style. Once your paper is typeset, you will receive an email with a link to choose the appropriate publishing options for your paper and our Author Services team will be in touch regarding any additional information that may be required. It is extremely important that you let us know now whether you will be difficult to contact over the next month. If this is the case, we ask that you send us the contact information (email, phone and fax) of someone who will be able to check the proofs and deal with any last-minute problems.

After the grant of rights is completed, you will receive a link to your electronic proof via email with a request to make any corrections within 48 hours. If, when you receive your proof, you cannot meet this deadline, please inform us at rjsproduction@springernature.com immediately.

Authors may need to take specific actions to achieve compliance with funder and institutional open access mandates. If your research is supported by a funder that requires immediate open access (e.g. according to Plan S principles or the NIH public access policy) then you should select the gold OA route, and we will direct you to the compliant route where possible. Because authors warrant under our subscription licensing terms that they haven’t committed to licensing any version of their article under a licence inconsistent with the terms of our agreement – including the applicable embargo period – publication under the subscription model isn’t suitable for authors whose funders require no embargo.

If you have any questions about our publishing options, costs, Open Access requirements, or our legal forms, please contact ASJournals@springernature.com

If you have posted a preprint on any preprint server, please ensure that the preprint details are updated with a publication reference, including the DOI and a URL to the published version of the article on the journal website.

You may wish to make your media relations office aware of your accepted publication, in case they consider it appropriate to organize some internal or external publicity. Once your paper has been scheduled you will receive an email confirming the publication details. This is normally 3-4 working days in advance of publication. If you need additional notice of the date and time of publication, please let the production team know when you receive the proof of your article to ensure there is sufficient time to coordinate. Further information on our embargo policies can be found here: https://www.nature.com/authors/policies/embargo.html

To assist our authors in disseminating their research to the broader community, our SharedIt initiative provides you with a unique shareable link that will allow anyone (with or without a subscription) to read the published article. Recipients of the link with a subscription will also be able to download and print the PDF.

As soon as your article is published, you will receive an automated email with your shareable link.

If you are active on Twitter/X or Bluesky, please e-mail me your and your coauthors’ handles so that we may tag you when the paper is published.

You can now use a single sign-on for all your accounts, view the status of all your manuscript submissions and reviews, access usage statistics for your published articles and download a record of your refereeing activity for the Nature journals.

Please note that you and any of your coauthors will be able to order reprints and single copies of the issue containing your article through Nature Portfolio's reprint website, which is located at http://www.nature.com/reprints/author-reprints.html. If there are any questions about reprints please send an email to author-reprints@nature.com and someone will assist you.

Please feel free to contact me if you have questions about any of these points.

Best regards,
Madhura

Madhura Mukhopadhyay, PhD
Senior Editor
Nature Methods

** Visit the Springer Nature Editorial and Publishing website at www.springernature.com/editorial-and-publishing-jobs for more information about our career opportunities. If you have any questions please click here.**

Open Access This Peer Review File is licensed under a Creative Commons Attribution 4.0 International License, which permits use, sharing, adaptation, distribution and reproduction in any medium or format, as long as you give appropriate credit to the original author(s) and the source, provide a link to the Creative Commons license, and indicate if changes were made. In cases where reviewers are anonymous, credit should be given to 'Anonymous Referee' and the source.
The images or other third party material in this Peer Review File are included in the article's Creative Commons license, unless indicated otherwise in a credit line to the material. If material is not included in the article's Creative Commons license and your intended use is not permitted by statutory regulation or exceeds the permitted use, you will need to obtain permission directly from the copyright holder.
To view a copy of this license, visit https://creativecommons.org/licenses/by/4.0/

Response to reviewers critics

We are grateful to the editors and reviewers for their thoughtful evaluation of our work. We believe that the revised manuscript is substantially improved. We have carefully addressed all reviewer comments, including clarifying terminology and better emphasizing the novelty of our approach within the context of existing literature.

In response to the reviewers' feedback, we have made extensive revisions, adding new comparisons to existing single-cell doublet detection methods, new simulations, more quantitative assessments, and additional analyses using publicly available datasets that extend beyond our original large-scale dataset.

With respect to novelty, we have clearly articulated what is new in this version. Compared with existing studies, our work provides a more comprehensive evaluation that includes systematic panel comparisons (notably incorporating the new 5K panel), sensitivity assessments, segmentation and background correction benchmarks, and a novel strategy for mitigating transcript spillover. Together, these additions strengthen the manuscript and clarify how our work offers a distinct and complementary contribution to the field.

Below, we provide a point-by-point response, with reviewer comments shown in *italic gray* and our replies in regular black font. All revisions in the manuscript are highlighted in red.

Reviewers' Comments:

Reviewer #1 (Remarks to the Author):

This manuscript investigated the quality and errors in Xenium data, including the latest 5K gene panel, using a large dataset consisting of over 40 slices of breast and lung tumor samples. This analysis revealed the prevalent problem of transcript diffusion in Xenium data and presented a method, SPLIT, to correct for the blurred gene expression signal due to transcript diffusion. This work has a high impact on the emerging Xenium data analysis. However, I have the following concerns.

We thank the reviewer for their positive assessment of our study and for recognizing the potential impact of our work on Xenium data analysis. We greatly appreciate the constructive comments and suggestions, which have helped us refine the conceptual framing, strengthen the validation of our analyses, and improve the overall presentation of the manuscript.

Major comments:

1. The main analysis is based on the labeling of doublets for each Xenium spot. In order for the conclusion to hold, the labeling of doublets should be very accurate. Despite that RCTD is generally an accurate deconvolution method, only using RCTD does not establish the reliability of doublet detection.

We thank the reviewer for raising this important point. We would like to clarify that, although RCTD uses the term doublet, in our analysis this does not necessarily imply the presence of a true biological doublet (i.e., an aggregate of two cells). Rather, we interpret these cases as instances of spatial mixing of signals from two different cell types within a Xenium location (usually a segmented cell). To avoid confusion, we have revised the terminology throughout the manuscript to make this distinction explicit. To avoid confusion with RCTD's terminology of singlets and doublets, we define pure-signal cells as profiles dominated by a single cell type, and contaminated-signal cells as profiles containing contributions from multiple cell types (e.g., due to spatial mixing of signal). We added a clarifying paragraph to the main text (see page 12, lines 365-373 or below) and throughout the manuscript, we refer to these simply as pure and contaminated cells.

"Importantly, Xenium cells classified by RCTD as "doublets" rarely represent true biological doublets (i.e., two cells segmented as one) but instead reflect mixed signals, also supported by the fact that "doublets" do not double in cell size (Supplementary Figure 15a). Since the distinction between singlets and doublets is based on a fixed threshold, many RCTD-derived "singlets" also exhibit secondary signals, in particular from neighboring cells (Fig. 3g, Supplementary Figure 15b), indicating that contamination can occur even in confidently annotated single-cell profiles. We therefore define cells with evidence of a secondary signal as contaminated and those without as pure, adopting terminology more appropriate for Xenium data and moving beyond RCTD's threshold-based classification."

(1) Are any of the scRNA-seq doublet detection method applicable to Xenium data if ignoring spatial coordinates? Do they identify consistent proportion of doublets or trends of doublet proportions as RCTD?

We thank the reviewer for this insightful question. We would like to clarify that RCTD does not use spatial coordinates; each Xenium cell/segment is deconvolved independently of its position.

Applying existing scRNA-seq doublet detection methods (e.g., Scrublet, DoubletFinder, scDblFinder) to Xenium data is problematic because these methods are tailored to droplet-based assays, where doublets arise from two whole cells captured in the same droplet or GEM. By contrast, in Xenium data, mixed profiles stem primarily from spillover signals of neighboring cells. In the revised manuscript (and detailed in other responses), we have clarified this distinction and refer to such cases as *contaminated cells* rather than true doublets.

Nevertheless, we applied scDblFinder to our Xenium data, a widely used single-cell doublet detection method that does not require prior knowledge of expected doublet rates. Although the absolute proportions of doublets identified by RCTD and scDblFinder differ (Supplementary Figure 9a duplicated below), in every sample the Chi-square test indicated a significant non-random association between the two classifications (Supplementary Figure 9b). Moreover, RCTD-identified doublets consistently exhibited higher doublet scores with scDblFinder and vice versa, and doublet scores derived from RCTD and scDblFinder were strongly correlated (Supplementary Figure 9c-d). This overall concordance supports the robustness of our RCTD-based classification. The remaining discrepancies likely reflect both

the differing assumptions of droplet-based doublet detection algorithms and the threshold-based nature of RCTD classification. It is also important to note that doublet detection tools for scRNA-seq exhibit variability even in their native setting, with results sensitive to doublet rate and sequencing depth (Xi & Li, *Cell Systems*, 2021).

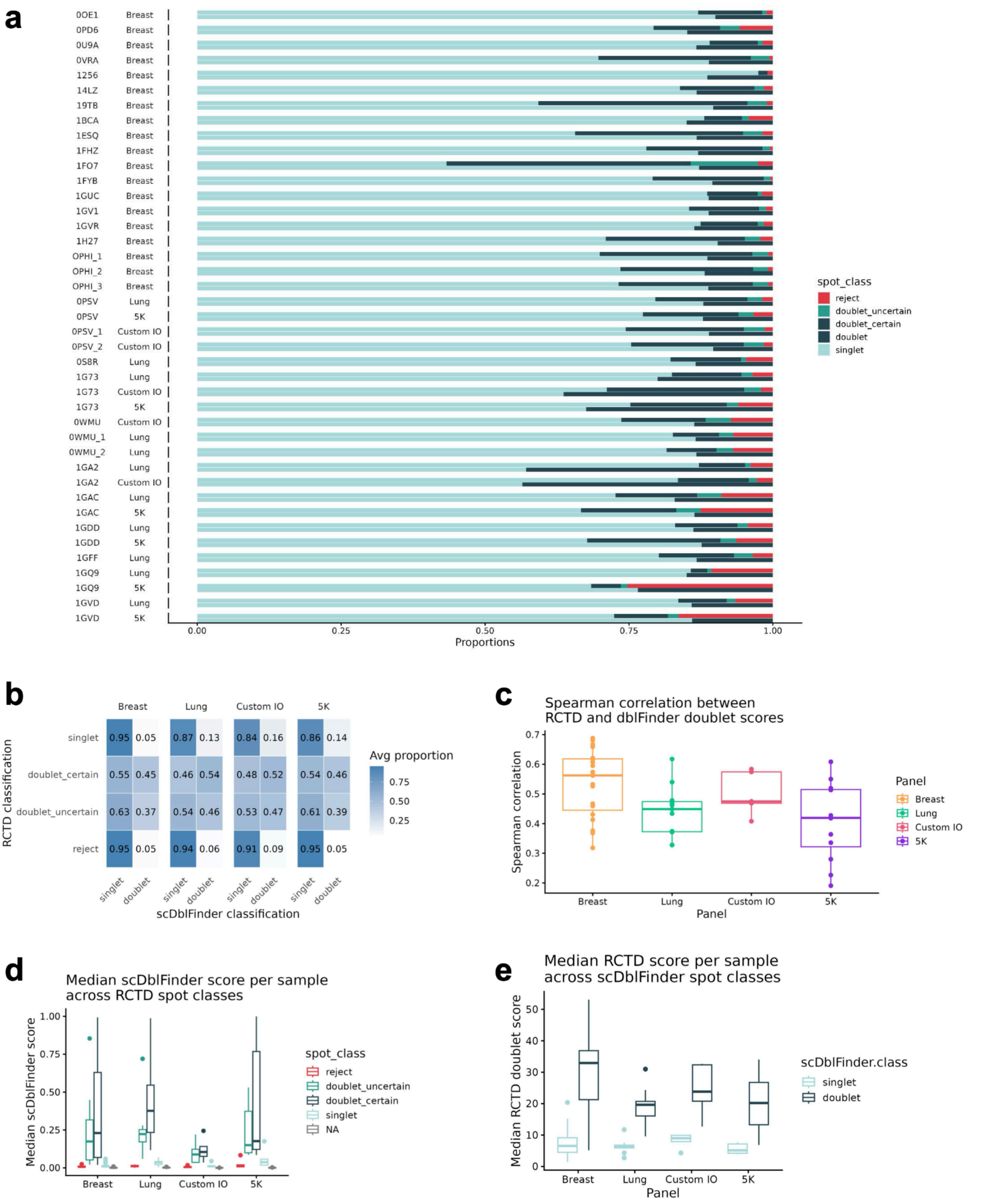

Supplementary Figure 9. Comparison of cell-class composition obtained from scDblFinder and RCTD doublet detection. a, Cell-class composition derived using RCTD (first bar) and scDblFinder (second bar). **b,** Confusion matrix between RCTD and scDblFinder cell-class assignments; values represent averages across samples. **c,** Spearman correlation between RCTD and scDblFinder doublet

scores, each dot corresponds to a sample. **d,** Distribution of median scDblFinder doublet scores per sample across RCTD-defined cell classes. **e,** Distribution of median RCTD doublet scores per sample across scDblFinder-defined cell classes.

(2). A recent method TACIT (https://www.nature.com/articles/s41467-025-58874-4) also mentioned identifying mixed spots and performing deconvolution for xenium data. How does the mixed spots identified by TACIT compared to RCTD?

We thank the reviewer for pointing us to TACIT. TACIT annotates cells based on signatures (e.g., reference profiles) and, for cells with ambiguous annotations, examines discriminative genes to define the primary signal. We applied TACIT to our data in two modes: (i) *signature mode* – as suggested in the original paper for Xenium data, using the top 10 most enriched differentially expressed genes per cell type; and (ii) *reference mode* – to ensure consistency with our RCTD run, using the same reference profiles. We made considerable efforts to incorporate TACIT into our automated processing pipeline; however, due to technical constraints, it had to be executed manually on a per-sample basis. Some runs failed to complete, and in several others the algorithm did not converge. We nonetheless include all the available outputs to provide a more complete comparison across samples.

Applying a similar comparison framework as for scDblFinder (Supplementary Figure 10), we observed consistent trends: a non-random association between TACIT and RCTD classifications (b), a positive correlation between TACIT and RCTD doublet scores (c) (for most of samples), and mutual enrichment of cells identified as mixed or doublets by either method (d,e). The main difference is that TACIT reported a substantially higher fraction of mixed (i.e., contaminated) cells compared with RCTD (a).

This discrepancy is likely due to methodological differences – RCTD assigns doublets based on a discrete threshold, whereas contamination is inherently continuous. Consequently, RCTD doublets represent cells with relatively high contamination levels, while many RCTD singlets still exhibit mild contamination (as was noted in the original manuscript and now expanded on page 12, lines 365–373). Accordingly, we now define pure cells as RCTD singlets without evidence of secondary signal and contaminated cells as those with any detectable secondary contribution, regardless of RCTD classification.

As TACIT provides cell-type annotation, we compared the agreement between TACIT and RCTD labels (Supplementary Figure 11). The agreement between RCTD and TACIT is reasonable (a) and comparable to the one between TACIT signature and TACIT reference mode (b).

Together, the higher fraction of mixed spots identified by TACIT and the elevated mixture scores among RCTD-defined doublets reinforce our conclusion that Xenium data are broadly affected by signal contamination, with RCTD doublets representing the most contaminated subset of cells.

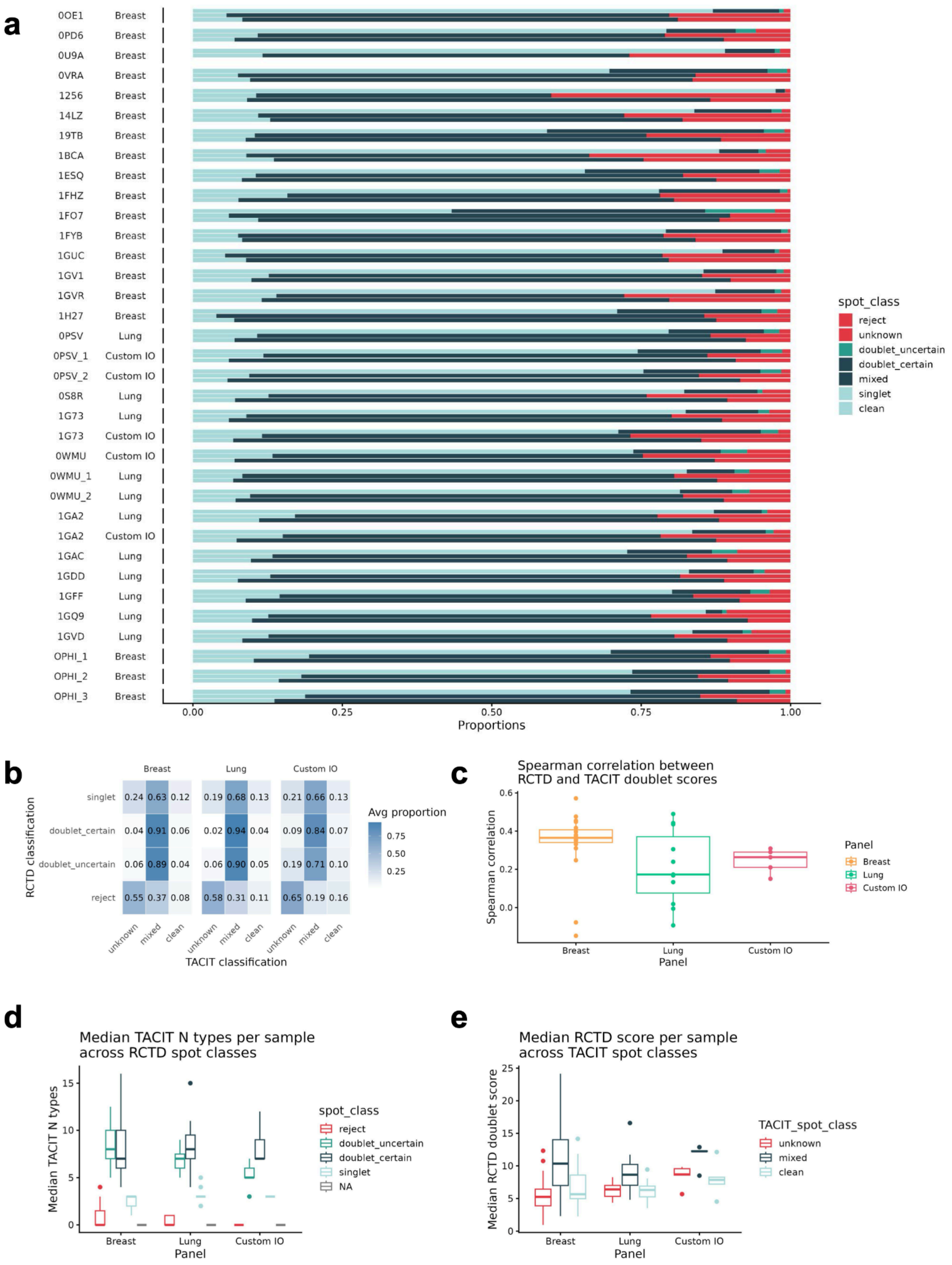

Supplementary Figure 10. Comparison of cell-class composition obtained from TACIT and RCTD deconvolutions. a, Cell-class composition derived using RCTD (first bar), TACIT in reference mode (second bar), and TACIT in signature mode (third bar). **b,** Confusion matrix between RCTD and reference-based TACIT cell-class assignments; values represent averages across samples. **c,** Spearman correlation between RCTD and TACIT doublet scores, each dot corresponds to a sample. **d,** Distribution of median TACIT doublet scores per sample across RCTD-defined cell classes. **e,** Distribution of median RCTD doublet scores per sample across TACIT-defined cell classes.

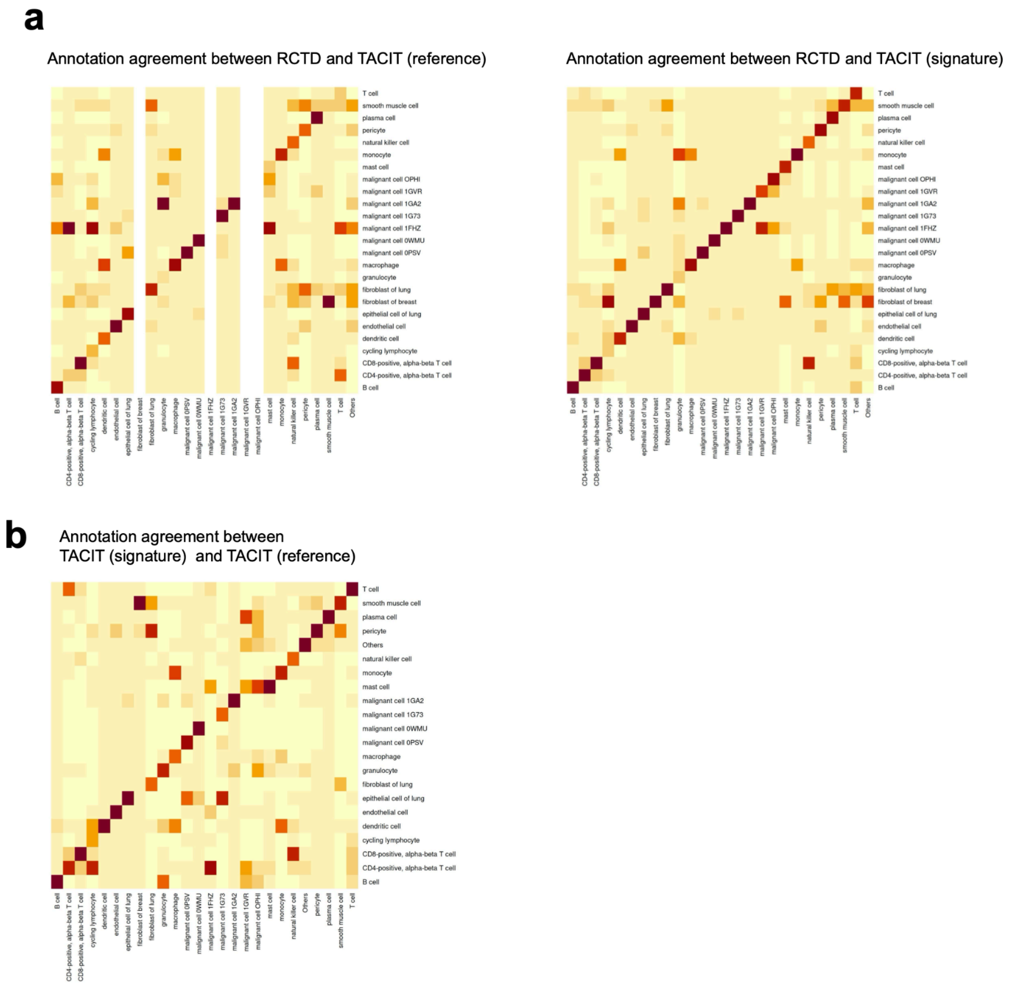

Supplementary Figure 11. Comparison of cell-type annotations obtained from TACIT and RCTD deconvolutions. a, Confusion matrices comparing cell-type annotations from RCTD (rows) and TACIT (columns) in both reference and signature modes. **b,** Confusion matrix comparing TACIT cell-type annotations between reference (rows) and signature (columns) modes.

(3). It may be possible to evaluate the potential errors when applying RCTD to deconvolve near-single-cell gene expression of a small gene panels by realistic simulated data. Particularly, the matched scRNA-seq suffer less from "transcript diffusion" problem, and therefore it's possible to simulate Xenium data by selecting genes and sub-sampling UMI counts from scRNA-seq data. Applying RCTD on the simulated data using scRNA-seq, and using another scRNA-seq as reference to avoid double dipping, the results are expected to be all singlets if the method doesn't have any errors. If the results are not all singlets, it will inform the potential error rates from the computational method.

We thank the reviewer for this thoughtful suggestion. Following the proposed approach, we simulated Xenium data from the matched Chromium dataset and applied RCTD using an external scRNA-seq reference to avoid double dipping. Across all tested panels (Lung, Custom IO, Breast, 5K), the results consistently showed lower number of doublets than in Xenium (Supplementary Figure 8b). This supports our conclusion that the observed doublets in real Xenium data arise not from the limited number of genes but rather from contamination signals intrinsic to the technology.

In RCTD, doublet scores are sums of per-gene contributions, so larger panels (e.g., 5K) naturally yield higher scores (Supplementary Figure 8a). Consequently, the higher doublet rate in 5K data reflects panel size rather than increased contamination. Doublet scores and rates are therefore only meaningful when compared within the same panel. We have now clarified this in the main text (see page 11, lines 307-312 or the paragraph below) and in the Methods section (see page 23, lines 726-738).

“RCTD doublet classification relies on a fixed threshold applied to a score that scales with the number of detected genes. As a result, panels with broader gene coverage, such as 5K Prime, tend to produce higher doublet scores (Supplementary Figure 8a). Consequently, doublet rates are not directly comparable across panels but remain informative for within-panel comparisons.”

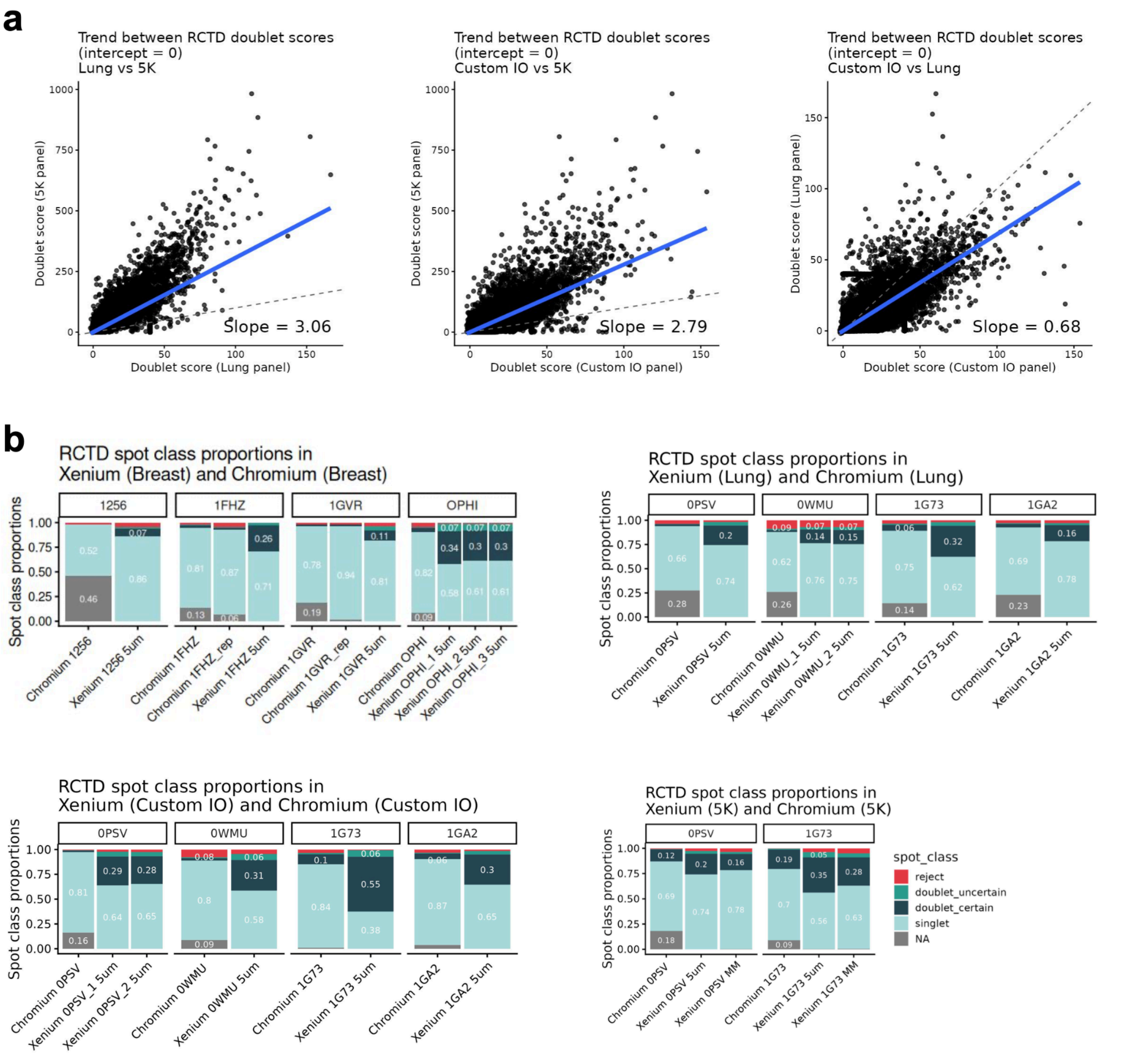

Supplementary Figure 8. Comparison of RCTD-derived cell-class composition in Chromium and Xenium data. a, Scatterplot of Chromium cell doublet scores when transcriptomic profiles are restricted to gene sets corresponding to different Xenium panels. Trend line fitted with intercept fixed at zero is shown in blue, x=y line is shown in dashed black. b, RCTD spot-class composition for

Chromium data restricted to the corresponding Xenium gene panels and for the matched Xenium samples.

2. Quantifying horizontal transcript diffusion is a fascinating idea. However, I have a few questions on the details of how transcript diffusion is quantified and analyzed.

(1). It's unclear to me what are possible explanations of "doublets" in xenium data, is it only horizontal diffusion and vertical diffusion? Particularly, the 5K panel has a lower degree of horizontal diffusion but higher rate of doublets, then what explains the high rate of doublets in 5K data?

We thank the reviewer for this important question. First, as explained previously, we have replaced the term doublet with mixed signal to avoid confusion, since RCTD doublets are not doublets in the strict sense. As discussed in the manuscript, mixed signals may result either from segmentation errors (where multiple cells are incorrectly segmented together) or from horizontal and vertical transcript spillover. Our data suggest that morphological segmentation is generally reliable (as confirmed by visual inspection and doublet size distribution in Supplementary Figure 15a), but transcript spillover is consistently present. While mixed signals tend to have slightly larger surface areas, they are primarily characterized by a higher number of detected features.

We believe the lower apparent horizontal diffusion in the 5K panel is primarily a consequence of reduced sensitivity: with fewer transcripts detected per gene, spillover events become less visible (hence the lower correlation, now replaced with the cosine similarity as suggested by one of the reviewers). Additionally, improved multimodal segmentation in this chemistry (Supplementary Figure 12c) may further reduce apparent diffusion.

The higher RCTD-derived doublet rate arises because doublet calling relies on a fixed threshold applied to a score that increases with the number of genes (reflecting a larger degree of freedom), making doublet rates incomparable across panels. For this reason, we have moderated our interpretation and revised the relevant text as follows (page 11, lines 337–342):

"The relatively lower cosine similarity observed for the 5K panel may reflect improvements in the updated chemistry and more accurate multimodal (MM) segmentation that together reduce spillover (Supplementary Figure 12c). However, this trend could also arise from the panel's lower per-gene RNA abundance, which may inherently limit detectable contamination, or from variation in RCTD decomposition due to the larger gene panel."

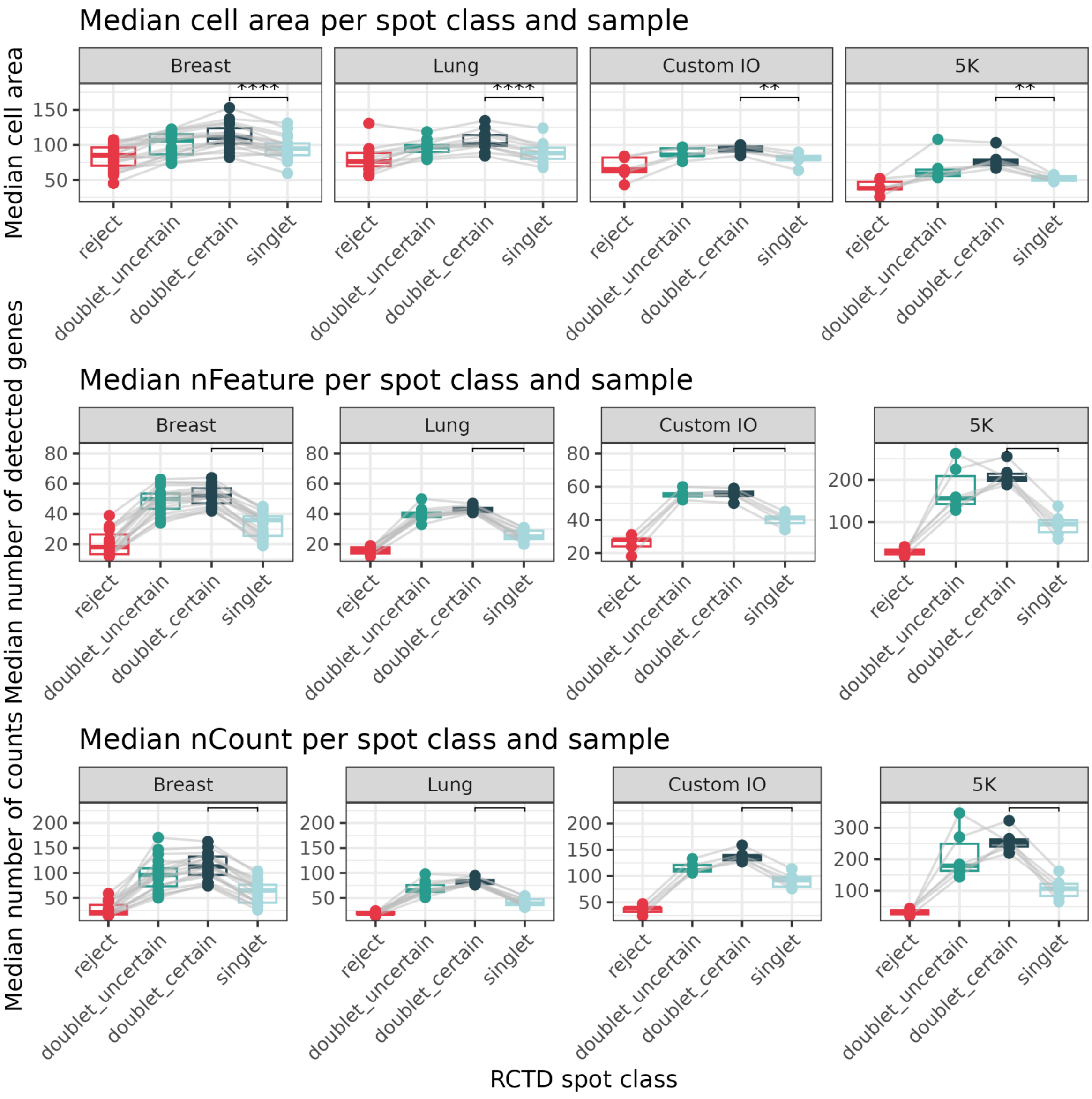

Supplementary Figure 15. Distribution of median cell area, number of detected genes and number of counts across RCTD-derived cell classes.

(2). The definition of diffusion index is hard to parse.

To clarify the definition of the spillover index, we first expanded the description of RCTD to make the notation explicit (see pages 10-11, lines 302-305) and added an extensive RCTD cell-type decomposition section into the Methods section (see page 23, lines 717-725). RCTD (in doublet mode) decomposes each spot (in our case, segmented cells) into a mixture of two cell types, assigning weights w_1 to the primary cell type and w_2 to the secondary cell type. We interpret the primary type (w_1) as the true identity of the cell, while w_2 reflects contamination from the secondary type. For any given pair of cell types, the spillover index quantifies how much of the contamination (w_2) can be attributed to spillover from neighboring cells of the secondary type. Specifically, we compute the correlation (replaced now with cosine similarity as suggested by one of the reviewers) between w_2

(across all cells with primary type 1) and the abundance of type 2 transcripts in the neighborhood. A higher cosine similarity suggests a greater evidence of spillover from type 2 into type 1. We have updated the method section (page 25, lines 802-813) and have added the following text on page 12, lines 343-349.

"To assess the susceptibility of each cell type to contamination, we defined a spillover index. For each pair of cell types, we calculated the cosine similarity between the contamination level of the primary (i.e., affected) cell type—quantified as the mixture proportion of the secondary type w2—and the local abundance of the secondary (i.e., contaminating) cell type in its neighborhood. Averaging these pairwise scores across all primary cell types yields a spillover index that summarizes the overall propensity of each cell type to be affected by contamination (Figure 3d,...)."

(3). It seems that the diffusion index computes the correlation between each pair of primary and secondary cell type. I didn't get why it is important to consider the primary cell type to quantify the tendency of secondary cell type to diffuse, instead of ignoring the primary cell type and compute a single correlation for each secondary cell type.

We appreciate the reviewer's thoughtful question. The main motivation for this analysis is that the impact of contamination depends on the overall transcriptional activity of the affected cell type. Less transcriptionally active cell types (i.e., those with fewer detected transcripts) are more susceptible to contamination, since even a small number of foreign transcripts can substantially distort their profiles. Pairwise analyses therefore make these effects easier to detect and interpret. Moreover, ignoring primary cell type could produce misleading patterns dominated by a few abundant populations. We have added a sentence to clarify this, see page 12, lines 350-351.

"The spillover index shows that more abundant cell types tend to contribute more frequently to contamination, with malignant cells showing the highest values."

(4). How many spots/numbers are used to compute each correlation value? If a secondary cell type is rare (say, only a few spots have cell type A as secondary cell type), then the correlation value may not be reliable.

The analysis was restricted to cell-type pairs with at least 20 observations. For each sample and these type pairs, we computed cosine similarity (previously Spearman correlation) and corresponding p-values. To account for multiple testing, p-values were adjusted using the Benjamini–Hochberg false discovery rate (FDR) procedure. The spillover index (previously "diffusion index") was then computed by averaging cosine similarities within each secondary cell type across all primary cell types and samples, treating non-significant (FDR ≥ 0.01) or insufficiently supported pairs (< 20 observations) as zeros. Each computed value is thus based on at least 20 observations, while rare secondary cell types with fewer observations are excluded to ensure reliability. We added this clarification to the Methods section (page 25, lines 805-813 and 811-813)

(5). Is diffusion index plot in Fig 3d computed using one lung sample or multiple lung samples? If computing on each individual lung sample, are the diffusion index consistent across slices for each patient or gene panels?

In Fig. 3d, the diffusion index is computed using all Lung samples, while results for the other panels are shown in Supplementary Figure 13a. When computed per sample, the diffusion index remains consistent for the most spill-prone cell types but varies for those with weaker spillover (Supplementary Figure 14). This variation can be explained by differences in cell-type abundance across samples, which are particularly pronounced among less spill-prone cell types. Across replicates, diffusion index values are overall highly similar (Supplementary Figure 13b).

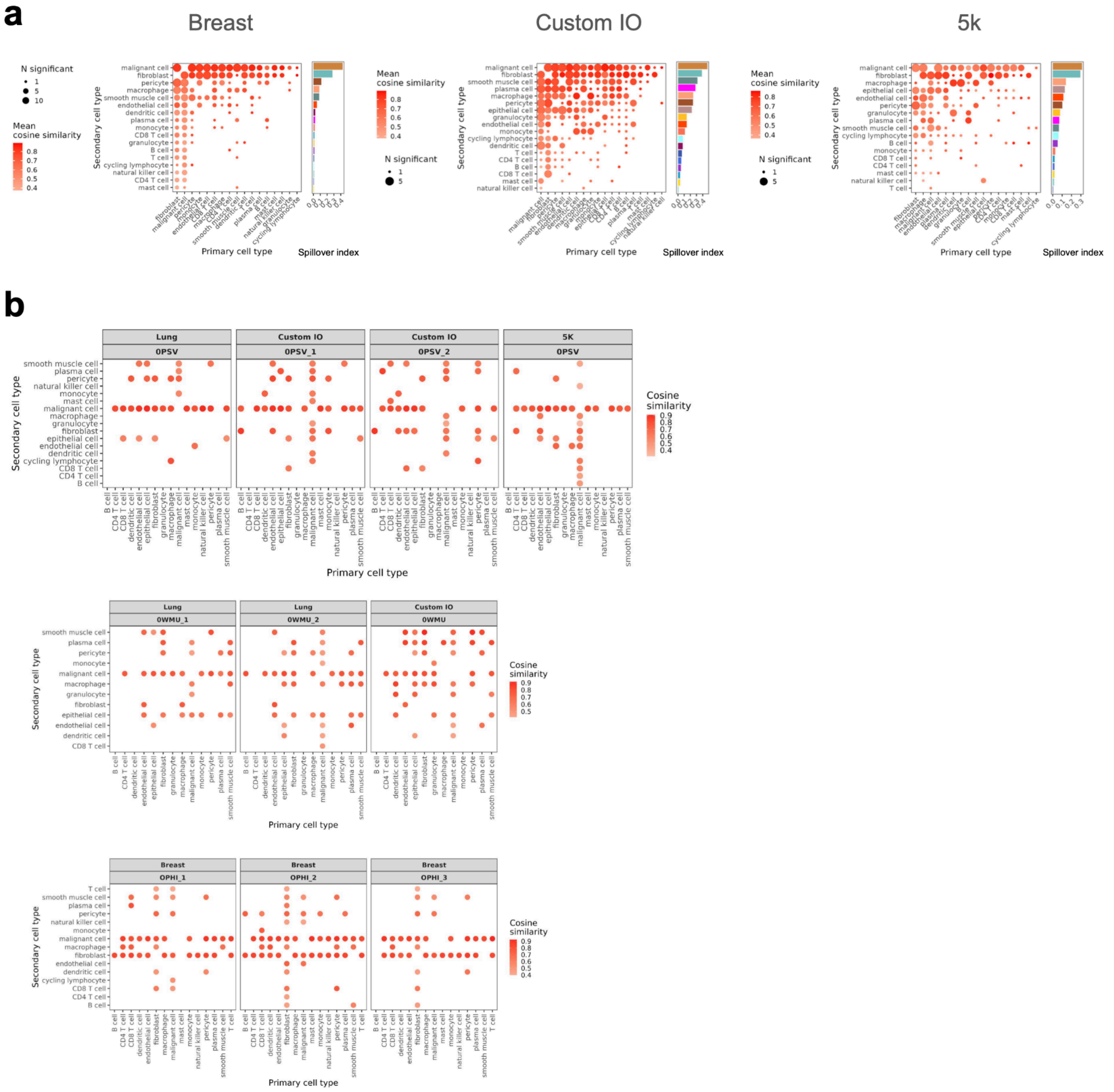

Supplementary Figure 13. Cell-type spillover index across panels and distribution of contamination level in doublets and singlets. a, Each dot represents the average Cosine similarity between a cell's secondary cell-type weight (w_2) and the proportion of that same cell type in its spatial neighborhood, computed for a given cell-type pair across all Xenium samples profiled with the Breast, Custom IO and 5K panels. Dot size indicates the number of samples in which the cosine similarity was statistically significant, and color denotes the average value. The adjacent bar plot summarizes these values per secondary (i.e., contaminating) cell type, representing the overall cell-type spillover index. **b,** Cosine similarity between cell's secondary cell-type weight (w_2) and the proportion of the secondary cell type in each cell's neighborhood, computed for all pairs of primary and secondary cell types across replicates. Only statistically significant values are shown (permutation test, n = 1000).

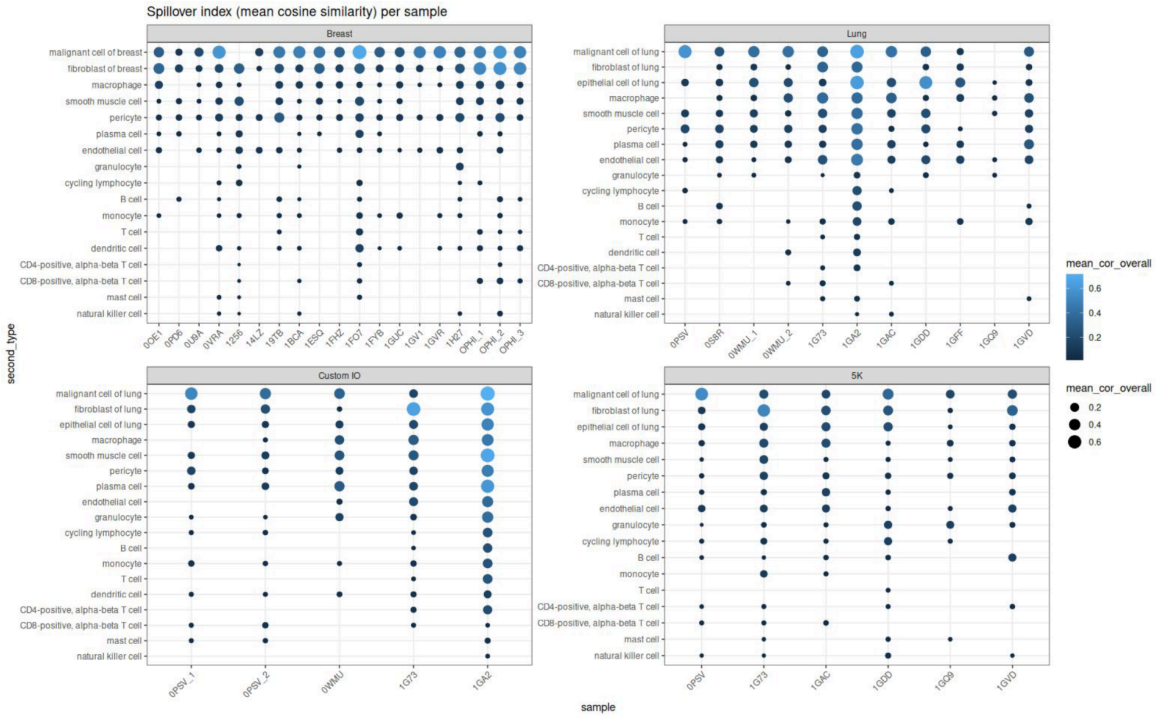

Supplementary Figure14. Cell-type spillover index per sample. The spillover index is defined as the mean cosine similarity between each cell's secondary cell-type weight (w_2) and the proportion of that same cell type in its spatial neighborhood, averaged across all primary cell types. When averaging, non-significant observations are set to zero.

(6). On line 421, a logistic regression model to predict secondary cell type based on neighboring cell type is mentioned, how does the logistic regression model compare to the diffusion index defined based on correlations?

We thank the reviewer for this interesting question. The spillover index quantifies correlations (cosine similarities) between neighborhood composition and contamination, whereas the logistic regression approach focuses on predicting T-cell versus non–T-cell identities of malignant cells from neighboring gene expression. While related in spirit, the two approaches address different questions and are therefore not directly comparable.

Moreover, the logistic regression model is multivariate, enabling us to consider all genes simultaneously when predicting cell types. In contrast to the spillover index, which is based on pairwise correlations, the logistic model integrates information across multiple genes while controlling for confounding effects, thereby capturing more complex and informative relationships between neighborhood expression and cell identity.

3. The doublet analysis is performed on the breast and lung sections generated by this study. Are the doublet proportions generalizable to other published Xenium slices if applying the same method on them?

Yes, we believe the results are generalizable. To support this, we have applied our method to a publicly available Xenium dataset and included the results in the Supplementary Material (Supplementary Figure 12a,b) and to the main text (page 11, lines 335-337). We observed a

high correlation between a cell’s contamination level and the presence of the contaminating cell type in its spatial neighborhood, along with consistent doublet and reject rates, as previously reported in our breast and lung sections.

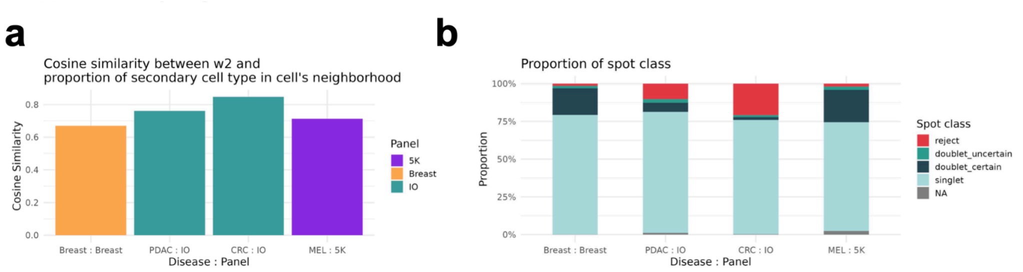

Supplementary Figure 12. Transcript spillover in public Xenium datasets and across segmentations. a, Cosine similarity between the w_2 (weight of the secondary cell type) and the proportion of the secondary cell type in each cell’s neighborhood in public Xenium datasets. **b,** RCTD-derived cell-class composition in the same public Xenium datasets. Each dataset is labeled as “Disease : 10x Panel.” Abbreviations: PDAC, pancreatic ductal adenocarcinoma; CRC, colorectal cancer; MEL, melanoma; IO, Immuno-Oncology panel; 5K, 5K Prime panel.

4. The separability of cell types in UMAP space across the technologies is only visually compared (Fig 2a, S5h, line 280). However, the visual comparison may be affected by many plotting artifacts, e.g., the number of points in the plot and whether or not using transparency. I suggest using quantitative metrics to indicate the separability of clusters, such as silhouette score, Calinski and Harabasz score, and Davies-Bouldin score.

We thank the reviewer for this important suggestion. To support our observations with unbiased analytical scores, we computed cell-type separation scores (Calinski-Harabasz, Davies-Bouldin, Leiden ARI, K-means ARI, label Silhouette) and batch integration scores (iLISI, batch Silhouette) and summarized then in Supplementary Figure 4.

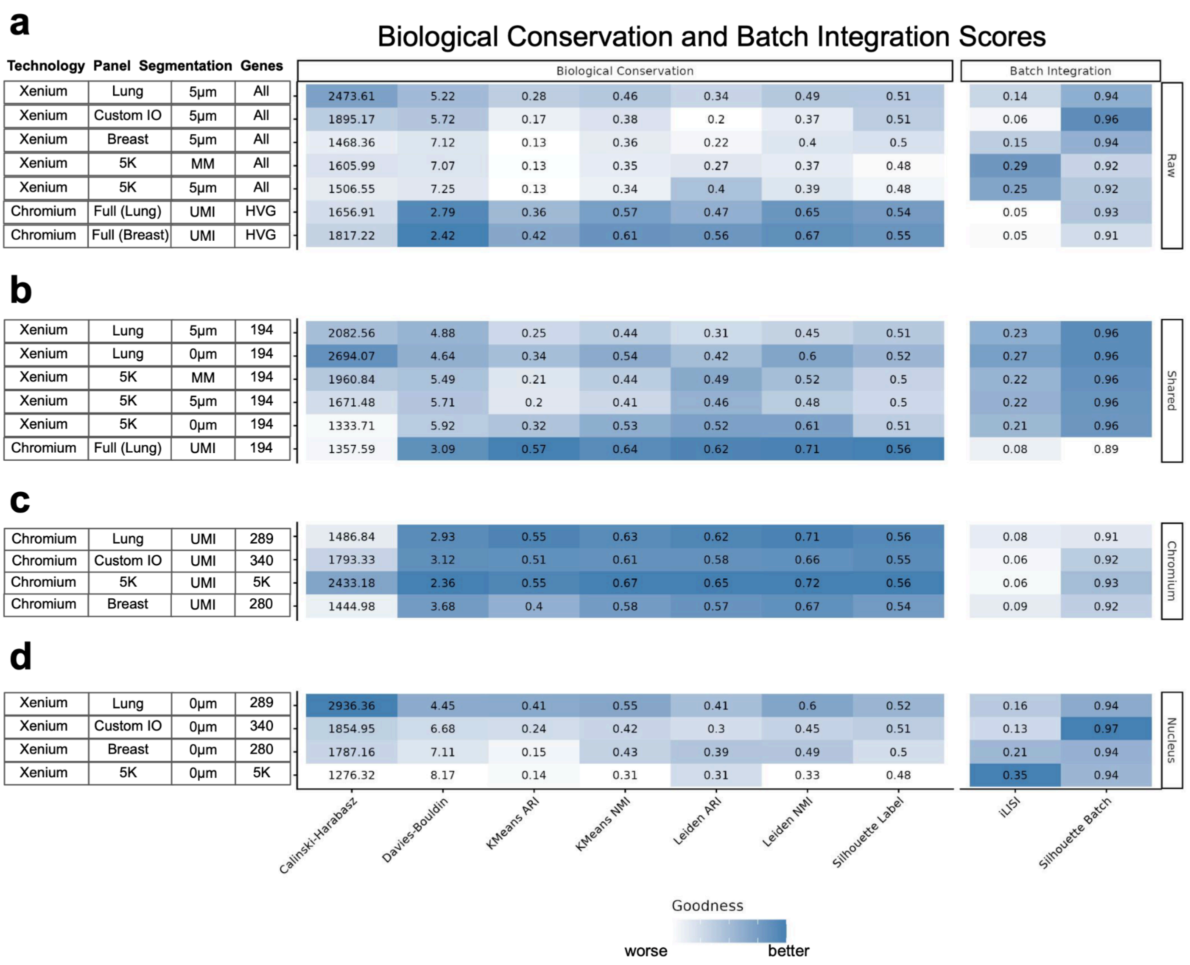

Supplementary Figure 4. Biological conservation and batch integration scores. a, Default data, including all Xenium datasets processed with default segmentations and Chromium datasets processed with the standard pipeline. Corresponding UMAPs are shown in Figure 1d. **b,** NSCLC datasets restricted to the 194 genes shared between 5K and Lung panels. For Xenium data, samples from six shared donors are included; for Chromium, all NSCLC samples are used. Corresponding UMAPs are shown in Figure 2a. **c,** Chromium data restricted to the genes present in the Xenium panel. Corresponding UMAPs are shown in Supplementary Figure 5h. **d**, Xenium data processed using nuclear segmentation. Color represents the goodness score, with darker shades indicating higher values; color gradient is scaled per column.

5. SPLIT is based on RCTD, which requires a reference scRNA-seq data. If scRNA-seq data of the same patient is not available, what's the accuracy of SPLIT using a different scRNA-seq reference?

We thank the reviewer for this important question. As noted in the manuscript (page 5, lines 156-158; page 19 lines 572-574; and page 24, lines 741-744), we do not rely on a reference dataset for each individual donor; instead, we use a pooled reference across all donors. Matched scRNA-seq data are available for 8 donors (4 in breast and 4 in lung cohort). To assess robustness, we also performed an analysis using an independent, publicly available scRNA-seq dataset as the reference (see Supplementary Figure 16b). The results were highly consistent, supporting that SPLIT is robust to the choice of reference dataset. We have clarified this point in the revised manuscript (page 14, line 424).

6. On line 329, "residual expression of transcripts from neighboring cell types is detected", for readers who are not experts in reading IHC images, is there an explanation on how to read the residual expression from the image?

The two main elements are the red and pink background stains, marking pan-cytokeratin–positive malignant cells (red) and CD8-positive T cells (pink). MUC1, F3, FASN, KRT7, EPCAM transcripts, which are cancer-specific, are shown as small brown dots and expected to localize primarily to malignant cells in the red regions. However, we also detect them in pink T-cell areas, where they should not be present. As this may not have been clear previously, we have revised the text to clarify it. Namely we now say the following:
"In particular, the IHC data allowed us to confidently identify $CD8^+$ T (stained in pink) cells and malignant cells (stained in red). Consistent with our hypothesis of physical transcript spillover, Figure 3a,f,g shows tumor-specific transcripts (small brown dots) extending into adjacent $CD8^+$ T cells, supporting the presence of spillover contamination."

7. In SPLIT method description, line 707 introduces the formula of x_{doublet-SPLIT}. Are ref_1 and ref_2 vectors? Are the computation elementwise? This should be clarified.

Exactly, ref_1 and ref_2 as well as observations (x_observed) are vectors, and the formulas imply element-wise division and multiplication. To clarify this, we have added vector notation to all formulas, replaced $\cdot$ symbol with $\odot$ to denote element-wise product, and included a clarifying sentence: *"Symbols '–' and '$\odot$' correspond to element-wise division and product, respectively."* We thank the reviewer for raising this point and helping us improve the clarity of the manuscript.

Minor:
1. "This is evident in Figure 3h, where singlet-classified cells still show signs of contamination", is it referring to a different figure panel as 3h is the illustration of SPLIT. Please also check the reference to other figure panels.

We thank the reviewer for their careful reading and for catching this mistake. We have corrected this error and also carefully reviewed all other figure panel references to ensure accuracy and consistency.

2. In figure 3g, does the y axis label "proportion of the tumor signal in cell's neighborhood" mean the proportion of spots whose primary cell type is tumor? Or does it mean other quantities?

By "proportion of the tumor signal in a cell's neighborhood" in Fig. 3e, we mean the average weight of the tumor cell type in the neighborhood, equivalent to the measure shown in Fig. 3b (bottom) as *"Proportion of secondary cell type in cell's neighborhood"*, where the secondary cell type is tumor.

Reviewer #1 (Remarks on code availability):

The code installation is smooth. But I recommend providing the memory and runtime information for the tutorial examples, which will be very useful for HPC computing job scheduling.

We thank the reviewer for noting that the code installation is smooth and for their valuable suggestion. Runtime information for the tutorial examples was already provided on the SPLIT README page. We have now added memory information (https://github.com/bdsc-tds/SPLIT/commit/a3cbddf).

Reviewer #2 (Remarks to the Author):

This manuscript presents a comprehensive analysis of signal quality in Xenium, with a particular focus on comparing whole transcriptome (5K) versus targeted panels. A key contribution is the introduction of the SPLIT method, designed to identify and address cell missegmentation, which is a known limitation in imaging-based spatial transcriptomics. The study highlights how misassigned transcripts can impact downstream analysis and proposes both empirical assessments and computational strategies to address this issue.
The study tackles an important problem in spatial transcriptomics and provides a valuable dataset with well-executed profiling of two tumor types. The comparison between panels and segmentation strategies is timely and informative. However, the manuscript suffers from some conceptual and presentation issues that limit its impact. In particular, the uncritical use of the term "diffusion" is problematic, and several analytical claims require more nuanced interpretation. With revisions, the manuscript could become a strong contribution to the field.

We thank the reviewer for their thoughtful and constructive evaluation. We greatly appreciate their recognition of the significance of SPLIT, the value of our dataset, and the timeliness of our comparisons. Their comments on terminology and interpretation are very helpful, and we have carefully revised the manuscript to address these points, specifically regarding diffusion, see below.

Major Comments

The most significant issue concerns the terminology and interpretation surrounding the concept of "diffusion." Throughout the manuscript, the authors use the term indistinctly to describe transcript spillover between cells. However, diffusion implies a specific biophysical process involving random molecular movement through a medium (e.g., 2D or 3D space). The manuscript does not provide evidence that such a process is occurring. In fact, reported findings—such as cell type–specific "diffusion"—suggest the phenomenon is more likely due to technical artifacts such as missegmentation or incorrect assignment of transcripts.
*I strongly recommend the authors reconsider the terminology used. More accurate terms would be *spillover*, *misassignment*, or *signal contamination*. Persisting with "diffusion" without clear justification introduces conceptual confusion and detracts from the rigor of the study.*

We thank the reviewer for this important clarification. To avoid conceptual confusion, we have replaced the term diffusion with spillover throughout the manuscript. We believe this

terminology more accurately reflects the technical nature of transcript misassignment and provides a clearer interpretation of our findings.

There are also some issues with respect to novelty for the first part of the paper (figs 1-3), where Xenium data are compared to single cell/nucleus data and other imaging based spatially resolved methods, and where the extent of contamination is evaluated. For the quantitative and qualitative comparisons of data set, there are now a number of papers presenting this, some cited by the authors, and for the contamination from neighboring cells, this has been shown and elaborated in the recent publication by Marco Salas, S. et al, Nat Methods 22, 813-823 (2025), a study not cited by the authors.

We thank the reviewer for this insightful comment. To clarify, our study does not compare Xenium to other imaging-based SRT platforms, but rather evaluates Xenium across different panels and, to a lesser extent, compares it with snRNA-seq (Flex). Our study offers a comprehensive evaluation, including systematic panel comparisons (notably incorporating the new 5K panel), sensitivity assessments, segmentation and background correction benchmarks, and a novel method for mitigating spillover.

We thank the reviewer for pointing out the reference to Marco Salas et al., which we were aware of but inadvertently omitted. We have now added this citation and discussed it in the Introduction in relation to our work (see page 3, lines 77-82 and 95-99; page 11, lines 323-326 or below). While Marco Salas et al. highlight background contamination in the context of optimizing nuclear extensions and variation along the z-plane, they do not address transcript spillover between cells or propose correction strategies. To our knowledge, our study is the first to systematically show that contamination is directly linked to the presence and abundance of contaminating cell types in the spatial neighborhood, providing broad and quantitative support for the concept of neighborhood-driven contamination.

We therefore believe our work provides a distinct and complementary contribution to the field, and we have revised the manuscript to make this perspective clearer.

page 3, lines 77-82 and 95-99:

"One of the most comprehensive analyses to date is that of Marco Salas et al.[12], who performed a systematic quality assessment of the Xenium platform by analyzing 25 primarily public samples across multiple tissues and benchmarking its performance against other spatial transcriptomics technologies. Their results consistently confirmed the high sensitivity of Xenium and provided valuable recommendations for analytical strategies."

"Indeed, Marco Salas et al.[12] reported contamination associated with cellular expansion in the standard Xenium segmentation, suggesting that transcript spillover within the x–y plane contributes substantially to background signal. However, aside from providing an automated method to adjust the expansion parameter, they did not propose a mechanism to correct this contamination."

Minor Comments

-Use of RCTD and cell decomposition: While valid, this is a highly specific approach. A short explanation of its niche applicability and limitations would improve accessibility for a broader audience.

We thank the reviewer for this important comment. We agree, we have added a summary in the methods section (page 23, lines 718-738) to recall the main idea behind the methodology and how we use it as well as a short explanation into the main text (pages 10-11, lines 303-307):

"...we used RCTD to annotate Xenium data, leveraging its doublet mode to model segmented cell profiles as a weighted mixture of two cell types, comprising a primary (true) type and a secondary (contaminant) type with corresponding weight w_1 and w_2. This decomposition was performed for all cells, after which RCTD classified each cell as a "singlet" or a "doublet"...".

We also added the following sentences to the discussion explaining its limitations (page 20, lines 603-605 and 614-615):

"An important consideration when using SPLIT is that it is currently relying on RCTD deconvolution. RCTD can assign multiple phenotypes to a single cell, requiring careful downstream interpretation of primary versus secondary identities. … Extending SPLIT to support other annotation and deconvolution frameworks is expected to mitigate the current dependency on, and limitations inherent to, RCTD."

-Figure 2b: The grey and blue colors are difficult to distinguish. Consider adjusting the color palette to improve visual clarity.

We have improved it (color palette in Figure 1a,b and Figure 2b has been updated).

-Line 54: The description of Xenium as an "in situ hybridization technique" is inaccurate. Xenium relies on rolling circle amplification and padlock probes. "Imaging-based spatial transcriptomics" would be a more appropriate term.

We have corrected it.

-Line 76-77: The authors mention that prior studies are limited in scale and patient diversity. However, this study is itself limited in tissue types, focusing on only two tumor types. This limitation should be acknowledged.

We thank the reviewer for this helpful comment. In the revised manuscript, we expanded our analysis to include four additional publicly available Xenium datasets, encompassing another breast sample, pancreatic ductal adenocarcinoma (PDAC), colorectal cancer (CRC), and melanoma samples profiled using three different panels (two targeted and one 5K Prime) (Supplementary Figure 12a,b). These additions broaden the scope and support the generality of the observed transcript spillover. We also explicitly acknowledge the remaining data limitations in the Discussion section (page 18, lines 538–542):
"Several of our findings were validated using additional publicly available Xenium datasets and we believe the results are generalizable. However, our primary analyses were limited to

two cancer types, and further validation across additional datasets and tumor contexts will be essential to confirm broader applicability."

-Based on our RCTD analyses..." (Methods section): This interpretation of mixed signals as "doublets" has already been described in Marco Salas et al. Please cite accordingly and clarify the novelty, if any.

We were aware of the study by Marco Salas et al. and regret that it was inadvertently omitted from our citations, most likely due to confusion with another related work in this rapidly evolving field. We have now properly cited it (Introduction, page 3, lines 77-82 and 95-99; Results, page 11, lines 323-326).

While Salas et al. addressed contamination in layered brain tissue, attributing it partially to z-tag overlap (~1.8% of cells) and primarily to domain-specific background noise, these mechanisms appear tissue-specific. In contrast, our analyses systematically demonstrate that in heterogeneous tumor tissue, contamination arises predominantly from neighboring cells. This is consistent with the findings of Mitchel et al., who reported neighboring cell contamination across multiple tissue types and SRT platforms, though their analyses were limited to selected cell-type pairs.

To our knowledge, our study is the first to systematically show that contamination is directly linked to the presence and abundance of contaminating cell types in the spatial neighborhood, providing broad and quantitative support for the concept of neighborhood-driven contamination. For importantly we were the first one to provide a mechanism to alleviate this problem. We had added more background information in the introduction.

-Figure 3c and correlation metrics: The observed lower correlation in the 5K panel is interpreted as reduced diffusion. This is a speculative conclusion. Other factors could explain the result, including differences in segmentation accuracy or RCTD performance. Additionally, correlation is sensitive to outliers; consider using cosine similarity or other robust metrics for this comparison.

We thank the receiver for this important comment and suggestions. We replaced correlation with the more robust cosine similarity as the reviewer suggested and obtained a similarly positive relationship between contamination magnitude and proportion of the contaminating signal in cells neighborhood (Fig. 3c).
We agree that the lower correlation/cosine similarity observed may be attributable to additional factors, such as variations in RCTD performance between panels. Accordingly, we have revised the paragraph and moderated the language as follows (page 11, lines 337 - 342):
"The relatively lower cosine similarity observed for the 5K panel may reflect improvements in the updated chemistry and more accurate multimodal (MM) segmentation that together reduce spillover (Supplementary Figure 12c). However, this trend could also arise from the panel's lower per-gene RNA abundance, which may inherently limit detectable contamination, or from variation in RCTD decomposition due to the larger gene panel."

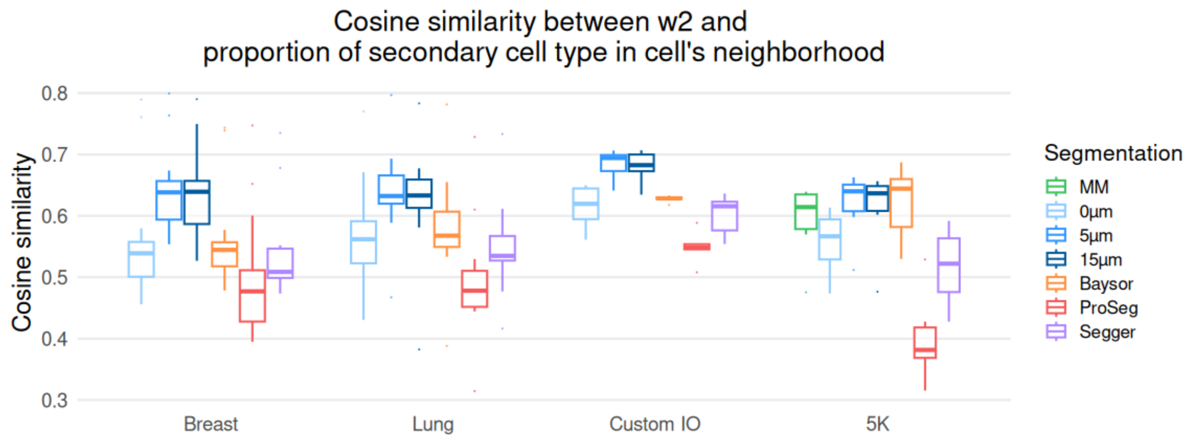

Supplementary Figure 12c. Cosine similarity between secondary cell-type weight (w_2) and the proportion of the secondary cell type in each cell's neighborhood in our data across different segmentation approaches.

-"Diffusion index" (Figure 3d): The notion of a "cell type–specific diffusion index" is contradictory if the process were truly physical diffusion—it should not vary by cell type. This again underscores the need to revise the terminology.

We thank the reviewer for this comment. We have revised the terminology and now call it cell-type-specific transcript spillover index.

-UMAP visualization: The manuscript refers extensively to UMAPs for interpretation. While useful for exploratory analysis, UMAP embeddings are sensitive to graph construction parameters, sample size, and preprocessing. Some caution in interpreting visual differences as biological should be added.

We thank the reviewer for this important caution. We agree that UMAP visualizations can be sensitive to parameter settings and sample composition. To ensure that our interpretations are supported quantitatively, we complemented the UMAP representations with analytical scores assessing clustering quality and integration performance, as summarized in Supplementary Figure 4 and Figure 4.

-Figure 4 and segmentation comparison: This is a valuable part of the manuscript but currently hard to interpret. The connection between SCIB integration metrics and segmentation accuracy needs stronger justification. Panels 4c and 4d seem tangential and could be moved to supplementary material.

We have added clarifications in the text to explain how these metrics relate to segmentation accuracy. Specifically, on pages 15 (lines 450-453) and 17 (lines 505-508), we now state:

"Although not the primary focus of our study, these same metrics can also inform comparisons between segmentation algorithms. A better segmentation approach should likewise improve cell type separation while minimizing batch variability. … ProSeg generally outperforms other algorithms, including Baysor, Multimodal, and Segger (Figure 4b).

Importantly, it also demonstrates that SPLIT can be combined with any segmentation tool, with SPLIT + ProSeg yielding the greatest overall gain."

Regarding panels C and D, we believe they provide valuable information on transcript and gene retention. This is an important metric, as some methods discard a substantial number of transcripts during correction, which can reduce statistical power. We therefore prefer to retain these panels in the manuscript.

-Line 515: The manuscript mentions niche analysis being impacted by signal contamination, but this is never demonstrated. Please provide supporting analysis or remove the statement.

We thank the reviewer for this observation. We have now adjusted the statement as follows (page 19, lines 558-560): "As noted by Mitchel et al.[15], such contamination can significantly distort the inference of cellular niches—one of the key goals of spatial transcriptomics."

-SPLIT limitations: The discussion should acknowledge that SPLIT requires a reference single-cell or single-nucleus dataset for decomposition, which may not always be available in all experimental contexts.

We thank the reviewer for this valuable comment. This limitation has been explicitly acknowledged and discussed in the Discussion section (page 19, lines 557–580):

"With the growing availability of reference datasets from initiatives such as the Human Cell Atlas[34], we anticipate that reference-based analyses will become increasingly accessible. Nonetheless, future work will focus on developing reference-free versions of SPLIT to further expand its applicability."

-It's not clear what Chromium chemistry was used to generate the snRNAseq data.

It is a Chromium platform with NEXT-GEM FLEX chemistry; we have added this information to the Data paragraph of Methods section, page 21, line 642.

Reviewer #3 (Remarks to the Author):

I co-reviewed this manuscript with one of the reviewers who provided the listed reports. This is part of the Nature Methods initiative to facilitate training in peer review and to provide appropriate recognition for Early Career Researchers who co-review manuscripts.

We thank the reviewer for their contribution to the evaluation of our work.

Reviewer #4 (Remarks to the Author):

Segmented cell data from Xenium are prone to mixed signals due to the presence of doublets and transcript diffusion across neighboring cells. To address this, the authors introduce SPLIT, a computational pipeline designed to decompose transcripts within segmented cells. The method applies RCTD, originally developed for Visium spot-level

deconvolution—to partition the transcripts of each cell according to its inferred mixture of cell types.

We are grateful for the reviewer's careful reading and for emphasizing the importance of quantitative validation, which has guided us in refining the analyses and strengthening the overall presentation of the manuscript.

While the study tackles an important methodological challenge, the analyses presented are largely descriptive and lack rigorous quantitative validation to substantiate several of the key claims. For example, the authors conclude from Figure 1e that "cells of the same type demonstrated strong concordance across samples without the need for batch correction." However, it is not evident what this conclusion is based on—whether it arises solely from qualitative inspection of the UMAP, or whether any formal metric of concordance was applied.

We thank the reviewer for this important comment. To quantitatively support our observations regarding batch integration and cell-type separation (i.e., biological preservation), we computed analytical scores that assess both integration quality and biological structure, as summarized in Supplementary Figure 4. These analyses were performed not only for the specific example referenced by the reviewer (Figure 1e, which is panel d in the revised version), but also more broadly to substantiate additional observations that were previously supported only by visual inspection. Furthermore, we demonstrate that cells cluster primarily by cell type, rather than by sample of origin (Supplementary Figures 2b and 3d), confirming that batch effects are minimal and that no explicit batch correction was required.

An important issue concerns the integration of spatial single-cell data across tissue samples. The manuscript does not provide sufficient methodological detail on how batch effects or sample-specific effects are addressed, if at all, during integration. Indeed, Figure 1d suggests that factors beyond cell type may be driving the observed separation among cells, raising questions about the robustness of the approach.

We thank the reviewer for raising this important point. As stated in the Results section, we did not perform explicit data integration across samples. When analyzing all samples jointly, we did not observe substantial batch effects. This observation is further supported by a favorable batch integration score (Supplementary Figure 4, Batch Silhouette) and by the clustering of cells according to their cell type rather than sample of origin (Supplementary Figures 2b and 3d).

Given that the merged data were used solely to assess cell-type separation (purity) and evaluate batch integration itself, we did not consider additional batch correction necessary. All other key analyses, including cell-type annotation and profile purification, were conducted independently per sample. Applying batch correction could introduce bias or distort biologically meaningful variation, as has been discussed in previous studies (e.g., Tran *et al.*, *Nat. Rev. Genet.*, 2020).

The discrepancy in cell-type composition (Figure 1d, now Figure 1c) are observed mainly between technologies (ie, Xenium and Chromium) due to the reason that in discussed in the main text:

"Figure 1c shows the distribution of annotated cell populations across individual samples. The data demonstrate strong reproducibility among technical replicates while revealing substantial variability between patients and between technologies—namely, Chromium and Xenium. This variability is expected: Chromium relies on dissociated single cells and typically requires larger input numbers, a process known to introduce biases in cell-type representation[23]. While snRNA-seq has been shown to mitigate some of these biases, it does not eliminate them entirely[24]. In contrast, Xenium profiles intact tissue sections, preserving spatial context and reducing dissociation-related artifacts."

Similarly, in Line 389, the authors state that "SPLIT-corrected cells reveal more distinct clustering and improved separation compared to the original mixed signals." Once again, this conclusion is presented in descriptive terms without supporting quantitative benchmarks. Formal statistical assessments would be essential to support this claim.

We thank the reviewer for this valuable comment. We would like to clarify that quantitative analyses supporting this observation are indeed provided in the subsequent section (Figure 4). In particular, Figure 4b presents analytical scores that quantitatively substantiate the improved clustering and separation observed for SPLIT-corrected cells.

To make this clearer in the text, we have revised the relevant section as follows (page 14, lines 442-425):
"UMAP embeddings of the resulting SPLIT-corrected cells (Supplementary Figure 16a,b) provide a visual example of improved clustering and separation compared to the original mixed signals, consistent across all panels and for both matched and external references. Quantitative validation of these improvements is provided in the following section."

Response to reviewers critics

We appreciate the acceptance in principle and the constructive feedback provided by the reviewers. We have carefully addressed all comments, including clarifying terminology, improving figures, and streamlining the text for clarity and conciseness.

Below, we provide a point-by-point response, with reviewer comments shown in *italic gray* and our replies in regular black font. All revisions in the manuscript are highlighted in red.

Reviewers' Comments:

Reviewer #1 (Remarks to the Author):

The revised manuscript is so much clearer and addressed my previous concerns. I have the following clarification questions that will further help me better understand the details.

We thank the reviewer for their positive assessment of the updated version of our study. We address the comments below and update the main text accordingly to facilitate clarification.

1. Main text line 332, "the local relative abundance of that same cell type in the surrounding 2D neighborhood", does the quantity refer to the proportion of neighboring cells with primary cell type being the same cell type, or the sum of RCTD weights of this cell type regardless of primary or secondary cell type?

By local relative abundance of a cell type, we mean the normalized sum of the RCTD weights for this cell type in doublet mode, regardless of whether it is assigned as the primary or secondary cell type, as demonstrated in Fig. 3b and highlighted in the figure caption. We have added this clarification to the main text:

For this, we compared contamination levels—estimated by the RCTD-assigned weight of the secondary cell type in each cell (w_2)—with the local relative abundance of that same cell type in the surrounding 2D neighborhood—estimated as normalized sum of the RCTD weights corresponding to this cell type *(Figure 3a,b).*

2. In Figure 1b, the sample/donor in cell count barplot panel is hard to tell partially because donors have different number of samples under different technologies. It would be easier to parse if donor/sample annotation is better denoted.

We thank the reviewer for this suggestion and have revised the figure accordingly.

3. In Figure 1c, there is no ambiguous/unannotated cells in Xenium samples, but Figure S1a contains "reject" and "unannotated" cells. Is Fig 1c plotted by filtering out unannotated cells or are the unannotated cells further manually annotated by some procedure?

Fig 1c plotted by filtering out unannotated cells (those cells usually have too few transcripts in common genes between single-cell and xenium). We have added a clarifying sentence in the Methods:

Due to the inherently lower UMI counts in single-cell resolution Xenium data, we applied a more permissive filtering strategy: cells with fewer than 10 total UMIs were excluded and do not appear in further plots, and UMI_min_sigma parameter was reduced to 100 to account for the reduced transcript coverage compared to spot-based spatial transcriptomics in the original RCTD application.

Reviewer #1 (Remarks on code availability):

The updated code is satisfactory to me.

We thank the reviewer for their positive assessment of our code.

Reviewer #2 (Remarks to the Author):

The revised version has improved a lot in terms of clarity. I still find the "singlet"/"doublet" terminology confusing and would recommend "pure"/"mixed" instead for clarity.
otherwise I think the revised manuscript is acceptable for publication. It is an interesting and important contribution.

We agree that the terms “pure” and “mixed” are clearer and more appropriate for Xenium data than “singlet” and “doublet,” and we now primarily use “pure”/“mixed” throughout the manuscript. The doublet-focused analyses have been moved to Supplementary Note 1, which largely eliminates the use of “singlet”/“doublet” terminology from the main text and improves overall clarity. Limited use is retained only where necessary to describe RCTD’s native output and introduce transcript spillover.

We applied RCTD in doublet mode to annotate Xenium data, modeling each cell as a mixture of a primary and secondary cell type with weights w_1 and w_2, and classifying cells as singlets or doublets (Supplementary Fig. 3a; see Methods). Importantly, RCTD “doublets” in Xenium rarely represent true biological doublets but instead reflect signal mixing (Supplementary Note 1), therefore we refer to cells with evidence secondary as “contaminated” and those without as “pure.”

We have also added another clarification regarding “doublets” into the introduction:

Transcript misassignments can result in the formation of artificial transcriptomic doublets (or multiplets), where gene expression signatures from different cell types become mixed and ambiguous. As we demonstrate later in this study, these mixed profiles do not correspond to actual physical doublets as defined in the single-cell sequencing field, but instead arise from signal-level mixing within spatial data.

Reviewer #2 (Remarks on code availability):

The code worked and instructions were sufficient. It runs a bit slow, and the authors could include some text in the manuscript about run-time and memory requirements.

We thank the reviewer for testing the code and for the positive feedback. We continuously optimize performance, and recent versions run substantially faster. Because run time and

memory requirements depend on dataset size and computing environment and evolve with ongoing improvements, we report and maintain these details in the GitHub repository, where they can be kept up to date and reflect the current implementation.

Reviewer #3 (Remarks to the Author):

I co-reviewed this manuscript with one of the reviewers who provided the listed reports. This is part of the Nature Methods initiative to facilitate training in peer review and to provide appropriate recognition for Early Career Researchers who co-review manuscripts.

We thank the reviewer for their time and for their contribution to the peer-review process.

Reviewer #4 (Remarks to the Author):

In response to my earlier comments, the authors have added calculations of scores for biological conservation and batch integration. However, several concerns remain.

Supplementary Figure 4 presents seven different biological conservation metrics across platforms and experiments. The reported values span a wide range—from less than 1 to over 1000—yet the manuscript provides no guidance on how these scores should be interpreted or what the color scales represent. Similarly, the authors state that "batch integration metrics further confirm strong concordance of cell types across samples," but it is unclear how this conclusion is supported by the table of numerical values, especially given the inconsistent color coding and lack of explanation. The same issue applies to the batch integration metrics, where results across methods are not always concordant and their implications are not clearly articulated.

We thank the reviewer for their evaluation. As the reviewer correctly points out, these established metrics span very different numerical ranges. To facilitate interpretation and enable relative comparison across methods, we therefore use a consistent color coding in Supplementary Figure 4 (Extended Data Fig. 2 in the current version) to guide the reader in assessing the relative "goodness" of each metric and added the following clarification into the Methods section:

Biological conservation of cell types and batch effect strength were assessed using metrics proposed by Luecken et al[26], as implemented in the scib-metrics package (https://github.com/YosefLab/scib-metrics) and Calinski-Harabasz, Davies-Bouldin scores from scikit-learn[35]. Higher scores indicate improved biological conservation or batch integration; Extended Data Fig. 2 uses color coding to facilitate interpretation and comparison across methods. Malignant cells were excluded before computing these metrics because they are often sample specific. Datasets were downsampled to a maximum of 100 000 cells to speed up calculations.

And to the Figure's caption:

Color represents the goodness score, with darker shades indicating higher (i.e., better) values; color gradient is scaled per column.

Regarding the batch integration results, we acknowledge that the two metrics do not always yield concordant conclusions. This is well known in the literature (Tran et al., Genome Biol 2020) as iLISI has increased sensitivity. We have slightly revised the text to reflect this and to tone down the message accordingly:

This indicates that both Xenium and Chromium platforms introduce little technical variation. The discrepancy between the two metrics reflects their differing sensitivities – iLISI captures local neighborhood mixing and is more sensitive, whereas Silhouette Batch evaluates global separation – an expected and previously reported phenomenon[27].

More broadly, the paper suffers from an overabundance of analyses and metrics. Numerous claims are made hastily without adequate explanation or justification. Several figures are overcrowded with panels, many of which are difficult to read or interpret. The manuscript would benefit greatly from a substantial rewrite and tightening of the narrative, ideally with active involvement from the senior authors to ensure clarity and coherence.

We appreciate the reviewer's thorough evaluation of our study. We agree that, during the revision process, many additional analyses were added in response to reviewer requests to better support and validate our original findings. We made a strong effort to integrate these analyses logically into the manuscript. While this inevitably increased the overall amount of analysis in the manuscript, these additions are intentional and serve to strengthen and further confirm our main conclusions.
To improve clarity and readability, and in line with the editorial guidelines, we have streamlined the main text and relocated several detailed analyses to the Supplementary Notes, thereby maintaining a focused narrative in the main manuscript while preserving transparency and completeness.
